# Spirulina *(Arthrospira platensis)* Used as Functional Feed Supplement or Alternative Protein Source: A Review of the Effects of Different Dietary Inclusion Levels on Production Performance, Health Status, and Meat Quality of Broiler Chickens

**DOI:** 10.3390/life14121537

**Published:** 2024-11-23

**Authors:** Antonia Lestingi, Mahmoud Alagawany, Alessandro Di Cerbo, Giuseppe Crescenzo, Claudia Zizzadoro

**Affiliations:** 1 Department of Veterinary Medicine, University of Bari Aldo Moro, 70010 Valenzano, Italy; antonia.lestingi@uniba.it (A.L.); giuseppe.crescenzo@uniba.it (G.C.); claudia.zizzadoro@uniba.it (C.Z.); 2 Poultry Department, Agriculture Faculty, Zagazig University, Zagazig 44519, Egypt; dr.mahmoud.alagwany@gmail.com; 3 School of Biosciences and Veterinary Medicine, University of Camerino, 62024 Matelica, Italy

**Keywords:** microalgae, poultry, feed supplement, alternative protein source, growth performance, gut health, liver health, oxidative stress, immunity, animal food

## Abstract

The broiler industry is pivotal in meeting the growing global demand for highly nutritious animal protein foods. Hence, there is a continuous interest in identifying novel, alternative, and even unconventional feed resources that could help sustainably support chicken meat production and quality. In this view, the microalga Spirulina (*Arthrospira*, formerly *Spirulina*, *platensis*), due to its unique chemical composition and some ecological advantages offered by its cultivation over traditional agriculture, has attracted great attention in the poultry sector for potential application in broiler diets, either as a functional supplement or a replacer of conventional protein sources such as soybean meal. The studies conducted so far seem to have confirmed many of the initial expectations regarding the advantages that may derive from dietary Spirulina supplementation, documenting its capacity to positively influence the intestinal and general health status of broiler chickens, leading to improved or preserved productive performance (under normal or challenging conditions, respectively), as well as to increased disease resistance and survivability. Furthermore, dietary Spirulina supplementation has been shown to induce positive changes in some important traits of broiler meat quality. However, at present, the inclusion of Spirulina in broiler diet, especially but not solely in relation to the use as an alternative protein source, presents several technical and economic limitations. To increase the overall awareness around the actual usefulness and practical usability of Spirulina as a novel natural component of the broiler diet, this review paper seeks to provide a comprehensive and integrated presentation of what is currently known about this topic, highlighting critical issues that are still pending and would require further research efforts.

## 1. Introduction

Using novel natural feed supplements and ingredients efficiently is an emerging approach to health-promoting diets and improved production and sustainability indicators in the livestock production sector [1,2]. Moreover, including natural products in livestock diets is regarded as a tool to compensate for the current limitations imposed worldwide on traditional antimicrobials due to the emergence of antimicrobial-resistant strains of pathogenic bacteria and protozoa [2,3,4,5,6,7]. The specific nature-based nutritional novelties that are attracting increasing attention worldwide for use in livestock farming include a vast array of biological and, in part, unconventional materials, such as probiotics and prebiotics, herbs and phytocompounds, mushrooms and yeasts, insects, macro-algal seaweeds, and unicellular microalgae [8,9,10,11,12,13].

Microalgae appear to have an exceptionally high potential for application in farm animal nutrition [1,14]. Indeed, microalgae have been defined as a “superfood” due to the excellent nutritional value determined by their rich content of many different macro- and micro-nutrients, which are difficult to find all together in other familiar food sources [15,16]. Moreover, using microalgae as alternative feed may help mitigate the environmental footprint of the agri-food sector [1,2,17,18,19]. For example, microalgae cultivation, especially when performed in photobioreactors, allows for higher land use efficiency than traditional terrestrial plant crops while also making it possible for producers to control the factors that are essential for microalgae biomass productivity, chemical composition, and overall quality (e.g., light conditions, temperature, pH, pCO_2_, nutrient supply, exposure to pollutants) [17,18,20].

Among the microalgae species currently commercialized for applications in the food and feed industry, *Arthrospira* (formerly *Spirulina*) *platensis* (*Spirulina*) has by far the largest global production volumes [14,18,20], probably also thanks to characteristics that are technically favorable to the cultivation and harvesting process [21]. The bulk of this production is currently sold for human dietary consumption (as powders, liquid extracts, tablets, drinks, snacks, and pasta), with a relatively smaller market in animal feed [21].

As presented in Table 1, the filamentous prokaryotic microalga Spirulina has an extremely high protein content (usually > 60% of dry matter) with a complete list of essential amino acids; moreover, it contains significant levels of carbohydrates, lipids, crude fiber, and minerals [14,19,22,23,24,25,26,27,28,29]. It is also rich in vitamins, with particularly high levels of Vit B_3_, Vit B_6_, Vit B_12_, and Vit K [26,30]. In addition, Spirulina contains high amounts of several functional physiologically active substances [27,31], including among others various pigments, such as carotenoid pigments (β-carotene), chlorophylls and phycobiliprotein pigment–protein complexes (C-phycocyanin, allophycocyanin), tocopherol, various phenolic acids (salicylic, trans-cinnamic, synaptic, chlorogenic, quinic, and caffeic acids), flavonoids, and polyunsaturated fatty acids (the essential γ-linolenic acid included and forming up to 40% of total fatty acids) [22,23,24,25,26,30,32]. Many of these compounds are known for their antioxidant activity and contribute to the marked antioxidant properties of Spirulina by interacting with each other or with other micro-nutrients [2,3,30,33,34]. Other valuable biological activities reported for the bioactive components of Spirulina include anti-inflammatory, immunomodulatory, hypolipidemic, prebiotic, antibacterial, antiviral, antifungal, antiprotozoal, and anticancer activity [2,3,20,35,36,37].

Due to this unique chemical composition, Spirulina has emerged as a promising candidate for inclusion in farm animal feed, either as a functional supplement or replacement for conventional and less eco-friendly protein sources, such as soybean meal [1,29,38,39], fishmeal, and groundnut cake [33,40]. In relation to these two potential in-feed applications, several studies have been conducted to scientifically verify the actual usefulness and practical usability of this microalga in the nutrition of various aquatic and terrestrial species of zootechnical interest, including ruminants, pigs, rabbits, fish, and poultry [1,3,22,33,41].

Considering the critical role that chicken meat production plays in fulfilling the increasing global demand for high-quality animal proteins [1,29,39,42], we thought it could be helpful to outline the types of information that may help commercial broiler producers, nutritionists, and veterinarians to make informed decisions about adopting Spirulina-based nutritional strategies. In this view, the present review aims to provide a comprehensive and integrated presentation of what is known from the published scientific literature regarding the effects that the varying dietary inclusion levels of Spirulina required for the two different in-feed applications mentioned above (i.e., “functional supplement” and “alternative protein source”) can produce in fast-growing broiler chickens, with particular regard to productive performance (including growth and carcass performance), health status (both intestinal and extra-intestinal, the latter related to the systemic antioxidant status, liver health, and immune system health), disease and stress resistance, and meat quality (various nutritional, physicochemical and sensory traits). Advantages, limitations, and critical issues that are still pending will be highlighted and discussed.

## 2. Methods

The literature search and article selection processes undertaken for this review were carried out according to a methodology that was adapted from the Preferred Reporting Items for Systematic Reviews and Meta-Analyses (PRISMA) guidelines [43] and their extension for Scoping Reviews (PRISMA-ScR) [44]. In July 2024, three electronic databases, including PubMed, Scopus, and Web of Science, were searched for papers published in English between January 1994 and July 2024. The search was performed using the following terms/phrases (in relation to article title, abstract, and keywords): “Spirulina AND broiler”, “Spirulina AND broiler AND feed”, “Spirulina AND broiler AND supplement”, “Spirulina AND broiler AND protein”, “Spirulina AND broiler AND performance”, “Spirulina AND broiler AND intestinal”, “Spirulina AND broiler AND digestibility”, ”Spirulina AND broiler AND antioxidant”, “Spirulina AND broiler AND metabolism”, “Spirulina AND broiler AND liver”, “Spirulina AND broiler AND kidney”, “Spirulina AND broiler AND immunity”, and “Spirulina AND broiler AND meat”.

The duplicates from the different databases were eliminated among the identified publications. The relevant sources were selected by verification that the study under examination targeted the use of Spirulina as a functional supplement or alternative protein source in the diet of fast-growing broiler chickens and reported information specifically related to its effects on the chickens’ productive performance (growth and carcass), health status (particularly intestinal health, systemic antioxidant status, liver health, and immune health), disease and stress resistance, and/or meat quality. This verification was initially made by reading the articles’ titles and abstracts and then the whole text. Articles that did not address the review questions were excluded. The references listed in the articles resulting from this selection were also consulted to identify additional scientific articles that could be relevant, including studies published outside the selected time frame.

## 3. Results

**Figure 1:**
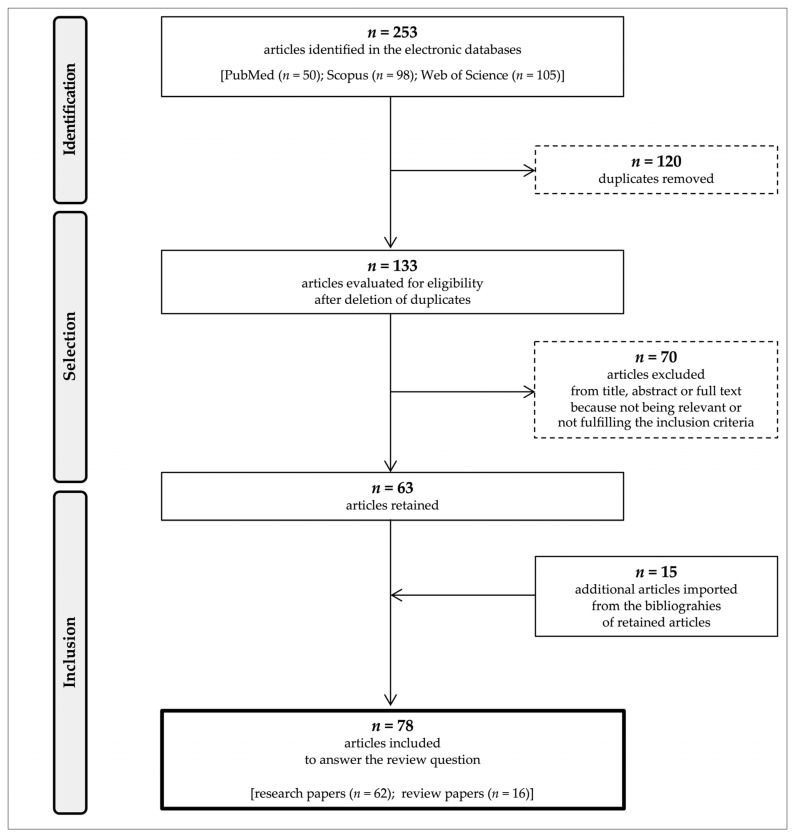
Flow diagram for article selection according to PRISMA guidelines.

The database query allowed the identification of 253 manuscripts (50 via PubMed, 98 via Scopus, and 105 via Web of Science). After removing 120 duplicates among the different searches, the remaining 133 articles were evaluated for eligibility. By sequentially evaluating the titles, abstracts, and full texts, 70 articles were excluded (because they were deemed not relevant or because of other reasons, such as the fact that the full text could not be found or was not in English or Spirulina was tested as the component of a mixture with other supplements). From the bibliographies associated with the retained articles, 15 additional relevant references were imported. Overall, 78 articles were selected for the present review, among which 60 research papers served as the basis for our discussion (Figure 1).

The main findings from these research papers are summarized in eleven tables (namely, Tables 2, 4–11, 13 and 14). Based on the review question, each table groups information about specific aspects of broiler chickens’ productive performance, health status, and/or meat quality on which the effects of dietary inclusion of Spirulina as either a supplement or a soybean replacer have been investigated (see the overview provided at the beginning of the Discussion section). Most of the tables group information collected under normal rearing conditions (namely, Tables 2, 4–8, 13 and 14), whereas some tables present information collected under challenging conditions (namely, Tables 9–11). Within each table, to facilitate comparisons between studies, the various Spirulina levels tested in the published literature are reported using the same measurement unit (i.e., final % of inclusion in feed or in drinking water), although in some cases this differs from that used in the original source publication. Whenever feasible, the selected research papers are listed within a table in increasing order of dietary Spirulina inclusion level and are mentioned and discussed in the main text according to the same sequence.

**Figure 3:**
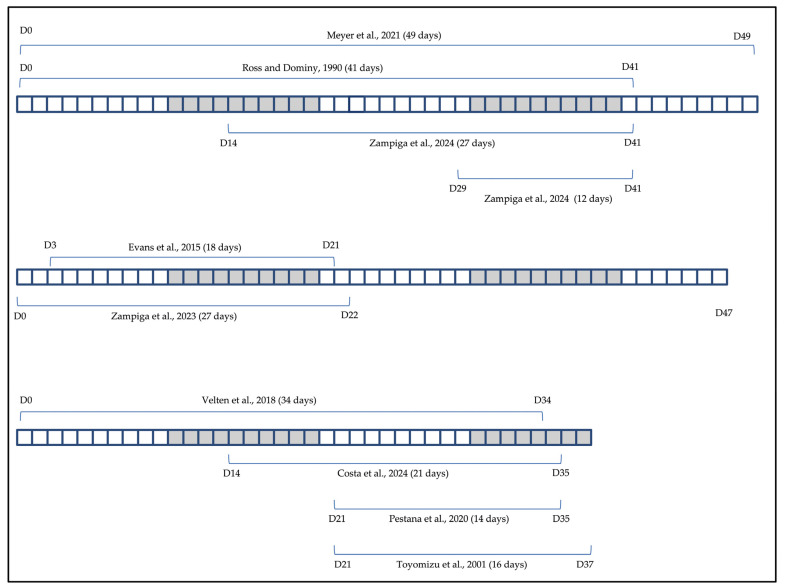
Overview of the studies evaluating the effect of feeding Spirulina-based (soybean-replaced) diets on the productive performance of broiler chickens, with a particular focus on the varying feeding regimens. The References analysed have been grouped on the basis of the overall duration of the rearing cycle [53,56,134,136,137,138,139,140,141]. Each square indicates a single day of the rearing cycle. Different colors have been used to facilitate the numbering of days (D = day).

**Figure 4:**
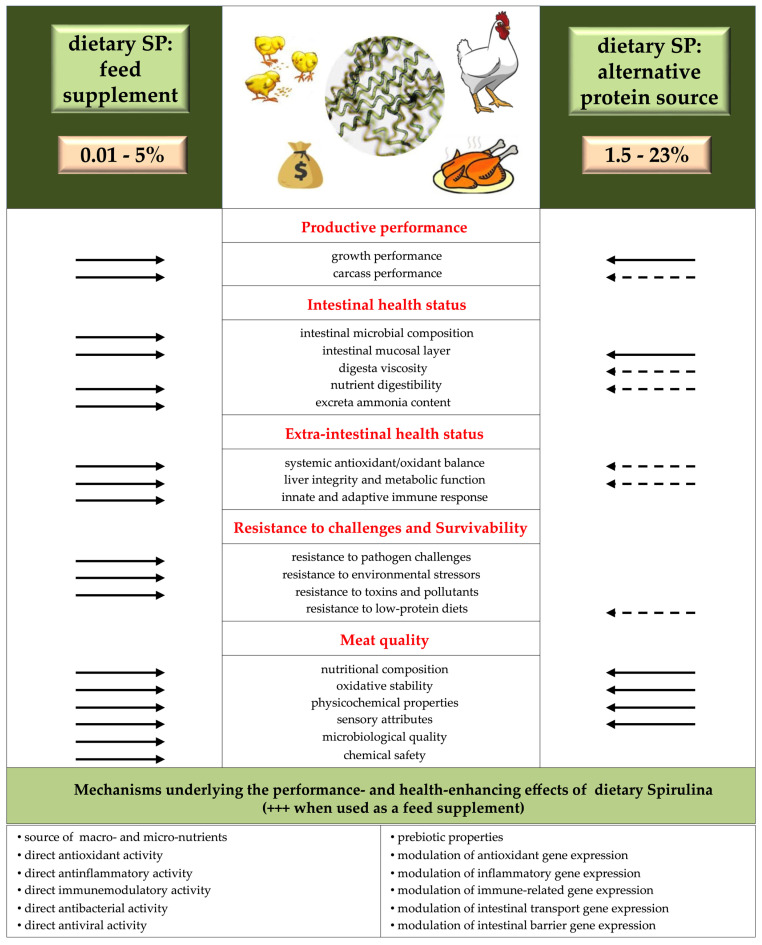
Aspects of broiler chickens’ physiology that Spirulina (SP) has been shown to influence when included in feed as either a supplement or a soybean replacer. Arrows indicate which aspects have been extensively (solid line) or partially (dotted line) studied in relation to the two different uses of dietary Spirulina.

Two additional tables (namely, Tables 3 and 12) and a figure (namely, Figure 3) were constructed to highlight peculiar aspects of the experimental conditions adopted in some of the studies examined for the present review that were deemed useful for the interpretation of some of the findings. Finally, a figure was created and placed in the last section of the discussion (namely, Figure 4) to provide an overview of all aspects of broiler chickens’ physiology that have actually been found to be responsive to dietary Spirulina when included in feed as either a supplement or a soybean replacer.

## 4. Discussion

**Figure 2:**
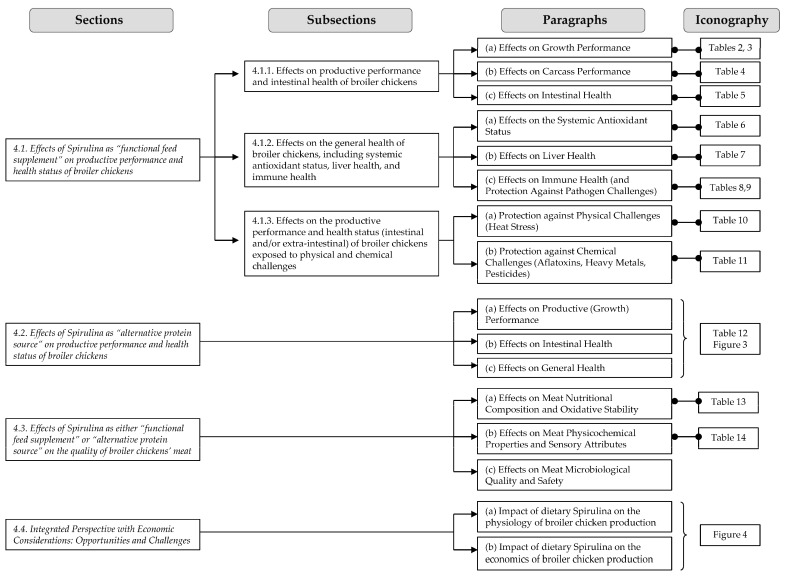
Overview of the various Sections, Subsections and paragraphs in which the Discussion is arranged, along with an indication of the Tables and/or Figures related to each part.

Below is an overview of the main points that will be addressed and developed to help the readers more quickly identify the sections and subsections of this discussion that deal with the information in which they are more interested (Figure 2).

### 4.1. Effects of Spirulina as “Functional Feed Supplement” on Productive Performance and Health Status of Broiler Chickens

#### 4.1.1. Effects on Productive Performance and Intestinal Health of Broiler Chickens

Several of the research papers included in the reviewed literature (25 out of 60) focused on the use of Spirulina as a feed supplement, evaluating its ability to positively influence the growth performance of broiler chickens reared under standard intensive conditions (Table 2). In many of these studies (13 out of 25), the impact of dietary Spirulina supplementation was also evaluated in terms of carcass performance (Table 3). Moreover, some research groups explored whether potential changes occurring in one or more aspects of the productive performance of broilers fed Spirulina-supplemented diets could be somehow related to concomitant changes in the intestinal health status of the animals (Table 3).

***(a) Effects on Growth Performance***

Based on the data available from the reviewed literature, broiler chickens fed Spirulina-supplemented diets may show either unchanged or improved growth performance parameters (Table 2). This different outcome, when reported by studies that tested the effects of dietary Spirulina across a range of graded supplementation levels [4,5,41,45,46], is in large part justifiable in light of the level of Spirulina inclusion in the broiler diet, with the lower levels tested being usually ineffective, or less effective than the higher ones at improving chickens’ growth performances. However, the impact of dietary Spirulina supplementation on the chickens’ growth performances can also be variable (i.e., able or unable to improve growth indices, and with different magnitudes of the possible improvements) when the same inclusion level of Spirulina is compared for its effectiveness across different studies [47,48,49,50]. This, more appropriately termed, inconsistency, is likely dependent on inter-study differences involving the several factors that have the potential to influence the animal response to a certain amount of Spirulina, and that includes the duration of the feeding period [2,6,51,52,53], the form of the Spirulina product administered (e.g., whole dried alga, powder, or liquid extract) [54], the mode of administration of the Spirulina product (via feed or via drinking water) and the method of the possible incorporation in feed [2,47,54,55], the chemical composition of the Spirulina product administered (which in turn can vary considerably in relation to the specific microalga strain, the microalga cultivation conditions, the post-harvesting processing methods, the storage conditions of the end-product) [1,2,6,19,23,26,31,56], the sex, age (growth phase) and genetics (specific broiler hybrid or strain) of the Spirulina-fed animals, the composition of the basal diet in which Spirulina is included, the housing and environmental conditions, and the type of production system [2,49].

From the perspective of inter-study comparisons, it is a fact that only a few of the experimental studies examined for the present review reported that dietary Spirulina supplementation was utterly unable to improve growth in broiler chickens (Table 2). More specifically, they are El-Bahr et al. (2020), who tested Spirulina at an inclusion level of 0.1% [47]; Qureshi et al. (1996), who tested four different dietary inclusion levels of Spirulina ranging from 0.001% to 1% (and including 0.01% and 0.1%) [57]; Bonos et al. (2016), who tested 0.5% and 1% Spirulina-supplemented diets [49]; and Mirzaie et al. (2018), who tested dietary Spirulina supplementation levels of 0.5%, 1%, and 2% [58]. By contrast, the majority of the studies showed that significant improvements in one or more growth performance indices can be obtained by supplementing the broiler diet with various appropriate levels of Spirulina, among which the levels labeled as “ineffective” by the abovementioned research groups are also included (Table 2).

**Table 2:** Overview of the studies evaluating the effects of dietary Spirulina supplementation on the growth performance of broiler chickens.

Spirulina Level (%)	Chicken Strain/Line	Feeding Period (Overall Duration)	Effect (Growth Indices Involved)	Notes	Reference
0.1	Cobb 500	From D4 to D36 (32 days)	No change (FBW, BWG, ADFI, FCR)	In this study, Spirulina was compared with two other microalgae species (*Chlorella vulgaris*; *Amphora coffeaformis*).	[47]
0.01–0.1–1	Arbor Acres	From D1 to D21 (21 days)	No change (FBW, FCR)	-	[57]
0.5–1	-	From D1 to D42 (42 days)	No change (FBW, FCR)	-	[49]
0.5–1–2	Cobb 500	From D17 to D38 (21 days)	No change (FBW, BWG, ADFI, FCR)	Starting from D38 (until D44), the chickens were exposed to heat stress to evaluate the performance protective potential of Spirulina.	[58]
0.03–0.05–0.07–0.09	Cobb	From D7 to D38 (31 days)	No change (FBW, BWG, ADFI, FCR) Improvement (↑ FBW, ↑ BWG, ↓ FCR)	The three effective levels were equieffective.	[4]
0.1	Hubbard	From D1 to D42 (42 days)	Improvement (↑ FBW)	-	[50]
0.1	Ross 308	From D1 to D42 (42 days)	Improvement (↑ BWG)	This study also evaluated the performance protective potential of Spirulina against exposure to AFB1.	[59]
0.1–0.2	Cobb	From D1 to D42 (42 days)	Improvement (↑ FBW, ↑ BWG, ↓ FCR)	The effect was dose-dependent; In this study, Spirulina was compared with another microalgae species (Chlorella vulgaris).	[60]
0.1–0.15–0.2	Arbor Acres	From D1 to D42 (42 days)	No change (BWG, ADFI, FCR) Improvement (↑ BWG, ↑ ADFI, ↓ FCR)	The lowest level (0.1%) only produced a slight increase in ADFI; The effect of the two higher levels (0.15 and 0.2%) was dose-dependent.	[45]
0.2	Cobb 500	From D1 to D42 (42 days)	Improvement (↑ FBW, ↑ BWG, ↑ ADFI, ↓ FCR)	In this study, Spirulina was compared with two other supplements (citric acid; cinnamon oil).	[61]
0.1–0.3–0.5	Cobb 500	From D1 to D35 (35 days)	No change (FBW, BWG, ADFI, FCR) Improvement (↑ FBW, ↑ BWG, ↓ FCR)	The effect of the two higher levels (0.3 and 0.5%) was dose-dependent.	[41]
0.2–0.4–0.8	Cobb 500	From D1 to D28 (28 days)	Improvement (↑ FBW, ↓ FCR)	The three effective levels were equieffective.	[62]
0.25–0.5–0.75–1	Ross 308	From D1 to D35 (35 days)	Improvement (↑ BWG, ↓ FCR, ↑ EPEI)	The effect linearly increased as the inclusion level increased.	[63]
0.5–1	Cobb	From D1 to D35 (35 days)	Improvement (↑ FBW, ↑ BWG, ↑ ADFI, ↓ FCR)	The lowest level (0.5%) only produced a decrease in FCR; In this study, Spirulina was compared with another microalgae species (Amphora coffeaformis).	[48]
0.25–0.5–1	Fayoumi	From D1 to D56 (56 days)	No change (FBW, BWG, ADFI, FCR) Improvement (↑ FBW, ↑ BWG, ↓ ADFI, ↓ FCR)	The lowest of the two effective levels (0.5%) only produced a decrease in FCR; In this study, the feeding behavior was also examined (decreased in response to 1% Spirulina).	[5]
1	Cobb 500	From D22 to D42 (20 days)	Improvement (↑ FBW, ↑ BWG, ↑ ADFI)	This study also evaluated the performance protective potential of Spirulina against Escherichia coli infection.	[64]
1	Cobb 500	From D1 to D35 (35 days)	Improvement (↑ FBW, ↑ BWG, ↑ ADFI, ↓ FCR)	In this study, Spirulina was compared with citric acid and their combination was also tested.	[65]
1–1.5	Cobb 500	From D1 to D35 (35 days)	No change (FBW, BWG, ADFI, FCR) Improvement (↑ FBW, ↑ BWG)	In this study, Spirulina was compared with the plant Moringa oleifera.	[46]
0.5–1–1.5	Vencobb	From D1 to D35 (35 days)	Improvement (↑ FBW)	The lowest and intermediate levels (0.5 and 1%) were equieffective, the highest level (1.5%) was the most effective.	[66]
2	Cobb	From D1 to D35 (35 days)	Improvement (↑ FBW, ↑ BWG, ↓ FCR)	This study also evaluated the performance protective potential of Spirulina against exposure to deltamethrin.	[67]
2	Ross 308	From D1 to D35 (35 days)	Improvement (↑ FBW, ↑ BWG, ↑ ADFI, ↓ FCR)	In this study, Spirulina was administered via drinking water; This study also evaluated the performance protective potential of Spirulina against Enterococcus faecalis infection, comparing it with that of prebiotics, probiotics, and oxytetracycline.	[68]
3	Cobb 500	From D1 to D35 (35 days)	Improvement (↑ FBW, ↑ BWG, ↑ ADFI)	This study also evaluated the effect of supplementing the Spirulina-containing diet with the carbohydrate active enzyme xylanase.	[69]
3	Cobb 500	From D1 to D21 (21 days)	Improvement (↑ FBW)	Starting from D22 (until D35) the chickens were exposed to heat stress to evaluate the performance protective potential of Spirulina.	[70]
1–2–3–4	Ross 308	From D1 to D42 (42 days)	Improvement (↑ FBW, ↑ BWG, ↓ FCR, ↑ EPEI)	All levels were equieffective at improving FBW, BWG, and EPEI; The improvement of FCR was recorded only with the highest level (4%).	[71]
1–2.5–5	Arbor Acres	From D1 to D38 (38 days)	Improvement (↓ FCR)	The effect produced by the highest level (5%) showed lower magnitude than that produced by the intermediate (2.5%) and lower (1%) levels, which were equieffective.	[72]

The “↑ ”or “↓ ”arrows indicate that an increase or a decrease, respectively, was observed in the parameter with the experimental diet (Spirulina-containing) compared with the standard broiler diet (control). Where two or more levels of Spirulina supplementation were tested, and only some proved effective, the effective ones appear underlined. **Abbreviations**: D = day; FBW = final body weight; BWG = body weight gain; ADFI = average daily feed intake; FCR = feed conversion ratio; EPEI = European production efficiency index.

**Table 3:** Percentage of total dietary soybean removed as an adjustment to broiler diets supplemented with varying levels of Spirulina.

Spirulina Inclusion Level							Reference
0.5%	1%	2%	2.5%	3%	4%	5%	
1.6	3.4	6.8	-	-	-	-	[58]
1.3 *–1.9 **^#^**	3.9 *–9.9 **^#^**	-	-	-	-	-	[48]
-	-	-	-	4.9 **–6.8 **^##^**	-	-	[69]
-	3.1	6.25	-	9.4	12.5	-	[71]
-	3.1	-	9.4	-	-	18.8	[72]

* Grower diet, **^#^** finisher diet; ** starter diet, **^##^** finisher diet.

The lowest level of dietary Spirulina supplementation that seems effective at improving chickens’ growth performances is 0.05%. These data derive from the study by Fathi et al. (2018) [4], who tested Spirulina at four different and relatively low dietary supplementation levels in a broiler diet (0.03, 0.05, 0.07, and 0.09%) [4]. No performance-related effect at all was observed with the lowest level (0.03%), whereas the other three levels (0.05%, 0.07%, and 0.09%) resulted equieffective in determining significantly improved live body weight (BW) (≈+6.5%), BW gain (BWG) (≈+7.1%), and feed conversion ratio (FCR) (≈−13.1%). Slightly more pronounced increases in BW (+8.7%) and BWG (+9.4%) were recorded at the 0.09% level, but the difference in the increases produced by the other levels was not statistically significant.

The growth-promoting efficacy reported by Fathi et al. (2018) for a broiler diet supplemented with 0.09% Spirulina appears consistent, at least in part, with the findings from two other studies that tested Spirulina at an inclusion level of 0.1% [50,59]. Notably, in one of these studies, the 0.1% Spirulina-supplemented diet resulted in increased final BW (+6%) compared with the control-fed chickens, though it did not cause any significant improvements in the BWG and FCR values [50]. On the other hand, in the other study, the same level of dietary Spirulina supplementation (0.1%) led to a slightly though significantly increased BWG (+2.8%), with no changes in the final BW and FCR values [59]. In closer agreement with the results obtained by Fathi et al. (2018), Abou-Zeid et al. (2015) reported that the dietary inclusion of Spirulina at 0.1% was effective at improving all performance indices of the chickens (final BW: +7.1%; BWG: +7.2%; and FCR: −9.8%) [4,60]. Moreover, in the same study, a two-fold higher level of dietary supplementation (0.2%) produced more pronounced improvements (final BW: +13.6%; BWG: +13.8%; and FCR: −14.4%).

Khan et al. (2020), instead, obtained disappointing results with 0.1% Spirulina-supplemented diets (only a slight increase in FI: +1.4%) but confirmed the growth-promoting efficacy of Spirulina at a 0.2% inclusion level, which resulted in improved BWG (+12.5%), FI (+8.95%), and FCR (−9.8%) in comparison with unsupplemented control birds [45]. Similar positive results regarding the growth-promoting efficacy of 0.2% Spirulina-supplemented diets were also reported by El-Sharnobey et al. (2023) (final BW: +11.2%; BWG: +15.2%; FI: +1.1%; FCR: −8.8%) [61]. In a recent study by Abdelfatah et al. (2024), a 0.1% dietary inclusion level of Spirulina proved again unable to exert any influence on the growth performance of broiler chickens; however, two higher levels of Spirulina supplementation, particularly 0.3% and 0.5%, were found effective at improving growth performance indices, with the higher level of supplementation (0.5%) performing better as a growth booster for broiler chicks than the lower one (0.3%), and leading, approximately, to a +5.6% increase in final BW, +5.7% increase in BWG, and −5.5% decrease in FCR [41].

A further two studies have substantially confirmed the overall growth-promoting efficacy of levels of Spirulina supplementation in the feed that is equal to 0.2–0.25%, or higher (0.4–0.5%), moreover also demonstrating this efficacy for inclusion levels up to 1% (0.75–0.8–1%) [62,63]. A difference between these two reports is that Jamil et al. (2015) (probably because of a relatively short feeding period of 28 days) found supplementation levels of 0.2%, 0.4%, and 0.8% to be equieffective at improving final BW (+6.7%) and FCR (−9.0%) of Spirulina-fed broiler chickens [62], whereas Park et al. (2018) demonstrated (after 35 days of feeding) that BWG, FCR, and the European production efficiency index (EPEI = BW/d × survival rate/FCR × 10) improved linearly (i.e., dose-dependently) as the dietary Spirulina inclusion level increased from 0.25 to 1.0%, with the most significant improvements (BWG: +4.2%; FCR: −2.9%; EPEI: +8.4%) being recorded with the highest level of dietary supplementation (1%) [63].

The superiority of 1% over lower levels of dietary supplementation with Spirulina, particularly 0.5%, was also reported by Alwaleed et al. (2021) and Hassan et al. (2022) [5,48]. In both of these studies, 0.5% Spirulina-containing diets were found to induce only a significant decrease in FCR (−8.9% and −9.4%, respectively), whereas dietary supplementation at 1% resulted in a significantly improved final BW (+25.9% and +12.6%, respectively), BWG (+12.1 and +13.2%, respectively), and FCR (−12.3% and −19.7%, respectively). It is interesting to note that while Alwaleed et al. (2021) found FI to be significantly increased with a 1% Spirulina-containing diet (+9.8%), Hassan et al. (2022) observed significantly decreased FI (−9.8%) along with a decrease in the feeding behavior, probably accounting for the considerable magnitude of the improvement in FCR values recorded in their study. In addition, it is worth noting that the significant chicken growth improvements reported by Hassan et al. (2022) in response to 1% dietary Spirulina were recorded only after 8 weeks (56 days) of feeding, that is, after a longer feeding time compared with the 35–42 days more commonly adopted in the feeding trials by other authors (Table 2) [5]. The fact that the experiment was conducted using a local chicken strain (Fayoumi) may account for the peculiar observations made by this group of investigators.

The efficacy of 1% Spirulina at improving broiler chicken growth performance when incorporated into the diet as a feed supplement was also reported by Alaqil and Abbas (2023) and Ismita et al. (2022) [64,65]. The first group of investigators recorded increased BW (+5.4%), increased BWG (+7.9%), and increased FI (+6%), with no change in FCR, in Cobb 500 chickens after 20 days of feeding (from day 22 to day 42 of age). Ismita et al. (2022), by subjecting Cobb 500 chickens to a more extended feeding trial of 35 days (starting from day of hatching), recorded a similar increase in FI (+5.9%) but more pronounced improvements in the other growth performance indices, with BW and BWG increased by +17.4% and +18.7%, respectively, and FCR showing significant decrease (−10.9%) relative to chickens fed the control diet. By contrast, Sharmin et al. (2020), although using the same chicken strain and duration of feeding period as Ismita et al. (2022), did not observe any significant growth improvements in response to 1% Spirulina-containing diets [46]. Some differences in the composition of the Spirulina products used in the two studies may have played a significant role in determining this different outcome, with the product used by Sharmin et al. (2020) probably containing fewer Spirulina-derived growth-promoting components than the product used by Ismita et al. (2022). Unfortunately, neither of the two studies (like many others in the literature reviewed) provided information regarding the chemical composition of the Spirulina product used, and this hampers the verification of this hypothesis. However, in partial corroboration of it, Sharmin et al. (2020) found that significant improvements in some growth performance parameters could be obtained (BW: +7.7%; BWG: +7.7%) when the broilers were fed a diet supplemented with a slightly higher number of Spirulina (1.5%). In the study by Khadanga et al. (2023), dietary supplementation of Spirulina proved able to improve chickens’ final BWs at either 0.5%, 1%, or 1.5%; however, the highest inclusion level of 1.5% was found to be the most effective (+13.4% versus +8.7 and +6.8%) [66].

In another study, evidence was provided that dietary Spirulina supplementation can also improve Cobb chickens’ growth performances at an inclusion level of 2% (BW: +10.3%; BWG: +11.1%; FCR: −9.4%) [67]. Interestingly, some authors obtained even more pronounced improvements in the growth performance indices by administering Spirulina at a 2% final concentration in the drinking water (BW: +12.5%; BWG: +19.3%; FI: +1.6%; FCR: −15.0%) [68]. The different modes of administering Spirulina and/or the different chicken strains (Ross 308) used by these authors are among the factors that may have contributed to the more positive outcome.

A positive influence of Spirulina as a feed supplement on the growth performance of Cobb chickens has also been reported for an inclusion level of 3% [69]. Notably, the authors of this study documented significantly and considerably increased BW (+50.0%), average daily BWG (+52.5%), and average daily FI (+42.4%) in the Spirulina-supplemented birds after 35 days of feeding, as compared with the control group; FCR was numerically reduced by 6.3%, but statistical significance was not achieved. In another report by the same research group [70], further confirmatory evidence was provided for the positive effects of a diet supplemented with Spirulina at 3% on broiler growth performance (increased BW), in this case, after a feeding period of a shorter duration (21 days).

Finally, based on the reviewed literature, the highest levels of Spirulina inclusion in broiler diets reported to be associated with improvements in one or more growth performance parameters were 4% [71] and 5% [72]. More specifically, in the study by Abbass et al. (2020), diets supplemented with Spirulina at 1%, 2%, 3%, and 4% were found to be equieffective at improving BW (+5.5%), BWG (+5.6%), and EPEI (+5.5%), with no changes in the FI; in addition, the 4% supplemented diet also resulted in a significantly improved FCR (−4.9%) [71]. In the study by Raach-Moujahed et al. (2021), all of the three dietary inclusion levels tested, namely 1%, 2.5%, and 5%, determined numerical increases in BW and BWG, and numerical decreases in FI resulted in significant reductions in the FCR value; however, with the diet containing 5% Spirulina, this effect showed lower magnitude than that obtained with the diet containing Spirulina at 2.5% (−7.7% and −15.4%, respectively) [72].

As we will also discuss later (see Section 4.2), it is worth noting that using Spirulina as a growth-promoting feed supplement may require some adjustments to keep the diet balanced [48,58,69,72]. Depending on [71] the nutritional quality of the Spirulina product used, some of the experimental studies examined reported adjustments to the diet even at supplementation levels as low as 0.5% [48,58]. These adjustments usually involved, among others, reducing some of the overall amounts of dietary soybeans [48,58,69,71,72]. For supplementation levels of up to 2–3% [58,69,71,72], the magnitude of this reduction did not exceed 10% of the total dietary soybean content, with 1.3–1.9% being the minimum amount of soybean removed in the formulation of 0.5% Spirulina-supplemented diets [48,58] (Table 3). However, when looking at studies in which Spirulina was added to the broiler diet at the higher supplementation levels of 4% [71] and 5% [72], the total amount of dietary soybean resulted to be reduced by 12.5% and 18.8%, respectively (Table 3). Therefore, in these cases, the boundary between using Spirulina as a “feed supplement” and using Spirulina as a “replacement of conventional protein sources” becomes less defined.

Staying on the subject of dietary Spirulina as a functional supplement with likely effectiveness at promoting the growth of broiler chickens, some additional considerations have to be made which are covered in the following two paragraphs.

***(b) Effects on Carcass Performance***

First, it should be emphasized that positive changes in the growth performance indices (particularly final BW) become more meaningful from the point of view of broiler meat production if they occur in association with a favorable carcass performance. In other terms, it is desirable that under the influence of Spirulina supplementation, the proportional contribution of the weights of the different parts of the animal body (i.e., bones, muscles, internal organs, abdominal fat, and so on) to an overall increased pre-slaughter BW varies in a way that an increase can be recorded in the relative weight of the whole eviscerated carcass (dressing percentage) and/or its “noble” edible components (meat yield). In contrast, a decrease should occur in the relative weights of waste internal components removed upon evisceration (e.g., the abdominal fat pad). The occurrence of no change in the carcass composition may be considered an acceptable (though nonoptimal) outcome. From this “carcass perspective” too, the effects of using Spirulina as a functional supplement in broiler diet seem generally positive (Table 4), as also commented by other authors [52]. Many authors consistently reported desirable improvements in one or more carcass-related parameters, who observed significant growth enhancement with Spirulina supplementation levels of up to 0.5% and included increased dressing percentage [41,45,50,60,61], increased relative weight of edible parts [41,60], and reduced relative weight of abdominal fat [60]. A noteworthy exception is the study of Fathi et al. (2018), where despite a favorably reduced relative weight of abdominal fat, a decrease in the relative weights of the half breast and half rear was recorded at all of the inclusion levels tested (0.03–0.05–0.07–0.09%) [4]. In studies testing higher levels of Spirulina supplementation, carcass-related parameters were reported to be either improved or unchanged. More specifically, Park et al. (2018) and Hassan et al. (2022), who obtained the most pronounced growth improvements with Spirulina inclusion levels of 1%, did not detect any significant changes in the dressing percentage [5], nor in the relative weights of breast muscle [63], abdominal fat [63], and internal non-immune organs [5,63]. Similarly, Sharmin et al. (2020), Ismita et al. (2022), and Mishra et al. (2023), who recorded significantly improved growth performance in response to 1.5%, 2%, and 3% Spirulina-supplemented diets, respectively, observed no changes in the dressing percentage [65], nor in the relative weights of breast muscle [69], abdominal fat [46], and internal non-immune organs [69]. Conversely, significantly (and equally) increased dressing percentage and breast cut percentage were reported by Abbass et al. (2020) in association with the growth-promoting effects of dietary Spirulina supplementation levels of 1%, 2%, 3%, and 4% [71]. Raach-Moujahed et al. (2021) also reported increased carcass relative weight in response to a growth-enhancing 2.5% Spirulina-supplemented diet, but no change was recorded in this carcass-related parameter with lower (1%) and higher (5%) growth levels of supplementation [72].

***(c) Effects on Intestinal Health***

Another critical point to be addressed in this discussion is how dietary Spirulina supplementation improves broiler chickens’ productive performances (growth ± carcass).

In all likelihood, a significant contribution derives from the excellent nutritional profile of this microalga [48,52]. Spirulina as a feed supplement, even when used in relatively small amounts, is widely recognized as a great source of highly digestible macro- and micro-nutrients necessary to fulfill the metabolic requirements of rapidly growing young chicks [71]. Among the most valuable nutrients supplied by Spirulina are high-quality proteins complete with all amino acids essential to support the development of the muscles and, hence, meat production [12,23,48,52,71]. Moreover, Spirulina is particularly rich in vitamins, minerals, and polyunsaturated fatty acids that, once absorbed, also play an essential role in supporting the chickens’ growth and all physiological functions [2,12,48].

In addition, the improved growth patterns observed in broilers fed Spirulina-supplemented diets may also be attributed to enhanced intestinal health, which in turn would lead to an overall improvement in the digestive function and improved absorption of the digested nutrients, as well as of dietary vitamins and minerals [2,23,65]. This possibility is suggested by the fact that increases in the chickens’ body masses (i.e., BWs) observed in response to dietary Spirulina supplementation occurred, although not consistently, in association with improvements in the efficiency of feed utilization (i.e., FCR) and unchanged levels of feed consumption (i.e., FI) [2,12,46,62].

Research specifically conducted to explore this possibility still needs to be completed. However, there is evidence in the reviewed literature that the inclusion of Spirulina as a supplement in broiler diets can exert beneficial effects on various aspects of the intestinal health of chickens (Table 5). Many of the chemical compounds abundantly contained in this microalga (as either structural components or bioactive metabolites) likely account for the production of these effects [65].

The influence of dietary Spirulina supplementation on the intestinal microbiota of broilers is the aspect most investigated. Various studies consistently reported that the growth-promoting effects of Spirulina-supplemented diets occur in association with an increased count of beneficial lactic acid bacteria (*Lactobacillus* spp.) [4,41,48,59,63,64,66,71]. It is of note that this desirable effect was detected with dietary inclusion levels of Spirulina as low as 0.05% [4], and it was found to increase linearly (i.e., dose-dependently) with increasing levels of Spirulina inclusion in a broiler diet ranging from 0.1 to 1% [41,63], as well as from 0.5 to 1.5% [66]. It has been suggested that the non-starch polysaccharides and chlorophyll in the microalga would provide substrates that facilitate the growth of lactic acid bacteria and act as prebiotics [23,48,63,73]. Less consistent findings regarding concomitant changes in the intestinal count of harmful bacteria like *Escherichia coli* have been reported. Indeed, some authors observed a desirable decrease in the number of Coliforms [4,48,59,64,66]. Other authors, however, reported no change in this parameter [63] or even a slight increase (at least with inclusion levels of 0.3 and 0.5%) [41], though always associated with improved growth performance. It has been proposed that the reduced intestinal count of *E. coli* could be the expression of the antimicrobial activity that Spirulina, likely by virtue of components such as phenolic compounds and fatty acids, among others [24,74,75], has been shown to exert, both in vitro and in vivo, against various Gram-negative and Gram-positive pathogenic bacteria, including *E. coli* [2,12,23,48,74,75,76]. However, the possibility also exists that the number of Coliforms in the intestine of Spirulina-supplemented chickens is reduced as a consequence of a lower pH of the intestinal environment (acidosis), actually documented by Alaqil and Abbas (2023) [64], which would result from the fermentations by the increased population of Lactobacilli and cause suppression of the growth of the harmful bacteria [60]. This scenario would more properly help explain the abovementioned inconsistency of the findings.

In the study by Abdelfatah et al. (2024), evidence was provided that, besides the overall positive modulating effects on gut microbiota composition (and partly as a plausible consequence of these) [41,50], dietary Spirulina supplementation in the range of 0.1–0.5% would also exert a beneficial influence on the structural and functional integrity of the intestinal mucosal layer [2,12]. More specifically, the authors reported that the jejunal mucosa of broilers fed Spirulina-supplemented diets, compared with that of birds fed control diets, was characterized by (i) increased gene expression of the fatty acid-binding protein 2 (FABP2), which suggests enhanced nutrient transportation and absorption [69]; (ii) increased activity of the endogenous antioxidant enzymes superoxide dismutase (SOD) and glutathione peroxidase (GPx), which suggests an increased capacity to fight against oxidative stress [69]; and (iii) decreased protein expression of the pro-inflammatory inducible nitric oxide synthase (iNOS) enzyme, which suggests reduced intestinal inflammation, likely contributing to improved utilization of the apparent metabolizable energy [2]. Further confirmatory evidence of reduced intestinal inflammation in the Spirulina-supplemented chickens was obtained by a histopathological evaluation of the mucosal tissue samples, which showed a less severe degree of enteritis (at least in response to the inclusion level of 0.3%) [41].

In the study of Khan et al. (2020), the growth improvement produced in broilers by a 0.2% Spirulina-supplemented diet resulted in significant improvements in the histomorphology of the small intestinal mucosa [45]. Particularly, the histomorphometric analysis revealed an (i) increased height of the villi, which is likely to result in an increased area for nutrient absorption [2], and an (ii) increased number of the mucin-producing goblet cells, which may translate into amelioration of the mucus layer that is crucial for the lubrication of feed, and physical protection against pathogens, toxins, and environmental irritants [77,78]. It must be pointed out that these morphological improvements were relative to a somewhat compromised control condition, where destruction of the epithelial lining and atrophy of goblet cells occurred in the intestinal villi [45] as a plausible consequence of the stressful influence exerted by intensive systems of poultry production [45,71]. Evidence of increased villi length and width in the small intestine of growth-improved broilers is also provided by Khadanga et al. (2023), following administration of diets supplemented with Spirulina levels ranging from 0.5 to 1.5% [66]. Other authors who observed similar intestinal morphological improvements in broiler chickens fed diets supplemented with other microalgae species indicated structural polysaccharides present in Spirulina as the microalga-derived compounds most likely responsible for these desirable effects [79]. The proposed mechanism is that they are fermented in the colon by commensal bacteria (including the abovementioned Lactobacilli), producing short-chain fatty acids (e.g., butyrate) [79,80,81,82,83,84,85]. The latter, besides exerting local anti-inflammatory activity, would play an important role (as a source of energy) in regulating epithelial cell proliferation, thereby supporting the development and differentiation of the intestinal epithelium in young chicks [2,86]. Moreover, short-chain fatty acids would stimulate mucus production and secretion [86]. Reduced inflammation and improved intestinal mucosa redox status may also account for the improved mucosal morphology [71]. In this respect, a “direct” contribution of dietary Spirulina also seems plausible through the supply of its antioxidant and/or anti-inflammatory components, such as phycocyanin, carotenoids, and polyunsaturated fatty acids. In corroboration of this hypothesis, a recent study by Omar et al. (2022) reported significant improvements in the histology and morphometric measures of broiler chicken small intestinal mucosa in response to dietary supplementation of Spirulina-derived phycocyanin [87].

It is noteworthy that in the study by Mishra et al. (2023), where feeding a 3% Spirulina-supplemented diet for 35 days resulted in a considerable increase in the chickens’ BWs but with no significant improvement in FCR, the histomorphometric analysis of the intestinal mucosa revealed only “numerical” improvements of the villus height and crypt depth as compared with the unsupplemented control group [69]. In any case, the same study provided evidence for significant and potentially favorable changes in the ileal relative expression of some genes that are related to (i) gut barrier function (mainly up-regulated expression of *Zonula occludens-1*—*ZO1* gene, which may result in reinforcement of the epithelial tight junctions); (ii) gut nutrient transport function (mainly up-regulated expression of the *Solute carrier family 7-member 7*—*SLC7A7* gene, which may result in enhanced transportation and hence absorption of L-amino acids); and (iii) gut immune homeostasis and developmental processes (mainly up-regulated expression of the *Cluster of differentiation 56*—*CD56* gene, which may result in more efficient immune surveillance and/or more appropriate intestinal tissue patterning during development) [69,88]. Taken together, these ileal gene expression findings suggest that, at this relatively high level of supplementation (3%), dietary Spirulina still has the potential to exert a beneficial impact on the structural and functional integrity of the intestinal mucosa of broiler chickens, even though it should be kept in mind that high gene expression does not necessarily translate into high protein expression, and hence does not necessarily equate to a significant effect on intestinal fitness.

A factual demonstration that, under the influence of dietary Spirulina supplementation, the healthier gut environment of broiler chickens (with its better-balanced microbiota and strengthened mucosa) can function more efficiently was provided by Park et al. (2018), who found feed digestion to be improved in broilers fed Spirulina-supplemented diets for 35 days [63]. More specifically, these authors observed that increasing the dietary inclusion levels of Spirulina from 0.25 to 1.0% resulted in a linear increase in the apparent total tract digestibility of dry matter and nitrogen [63]. Moreover, as a likely consequence of the improved digestion of dietary nitrogen (which allows less availability of nitrogenous compounds for degradation by bacteria in the large intestine), a dose-dependent decrease in excreta ammonia gas content of the supplemented broiler chickens was also recorded in this study [63]. This finding, similarly reported for growing pigs [22], may lead to potential ecological advantages [73].

Besides the exertion of a positive influence on gut health, and possibly also because of it [2,12,60], dietary Spirulina supplementation has been reported to improve broiler chickens’ general (extra-intestinal) health. These systemic health-enhancing effects of Spirulina, which will be discussed in more detail in the following subsection of this review (Section 4.1.2), may be another plausible reason for the overall improved productive performance of the chickens [2,71]. Indeed, a healthier animal should be prone to eat more (which would account for the reported findings of increased FI) and, most of all, should be capable of more efficient metabolic utilization of the energy and nutrients supplied by the digested feed (which would contribute to the reported favorable decreases in FCR values) [2].

#### 4.1.2. Effects on the General Health of Broiler Chickens, Including Systemic Antioxidant Status, Liver Health, and Immune Health

Various studies in the reviewed literature evaluated whether dietary Spirulina supplementation could exert any beneficial effects on broiler chickens’ general health similar to those reported for mammalian species [30,35,89]. In some of the studies, this evaluation was essentially performed in relation to the positive implications that an improved health status may have on the growth and carcass performance of the birds (as mentioned above). In some other studies, the health-related evaluations were mainly justified in light of the positive implications that an improved health status may have on the disease resistance of the birds and their overall survivability rate (which also are essential aspects to be considered in the economy of broiler meat production). In both cases, rather convincing evidence has been provided that including Spirulina as a functional supplement in broiler diets can improve the health of the chickens’ essential physiological systems and functions (besides the digestive one).

***(a) Effects on the Systemic Antioxidant Status***

The antioxidant system is one of the chickens’ physiological systems favorably influenced by dietary Spirulina supplementation. In this regard, it is worth recalling that broiler chickens, due to the high metabolic rate associated with their rapid growth and the stress related to intensive breeding, produce high amounts of free radicals and, hence, need an exceptionally robust arsenal of antioxidant defenses to protect their cells and tissues against oxidative stress and its damaging action [71,90,91]. The microalga Spirulina can be expected to enhance the antioxidant capacity of the animal body and thus help maintain a favorable oxidant/antioxidant balance [2,12]. This, in the first place, is because Spirulina is a rich source of antioxidant compounds (e.g., C-phycocyanin, β-carotene, and phenols) (Table 1) that can act directly as free radical scavengers in all of the body’s compartments to which they have kinetic access [30]. In the second place, there are data from animal models indicating that Spirulina can enhance the organism’s endogenous antioxidant defenses by increasing blood activity levels of antioxidant enzymes like SOD and GPx [35], and this, at least in part, is possible because some of the micromineral elements supplied by Spirulina (e.g., zinc) serve as cofactors for these enzymes [92,93,94,95].

Findings from the relatively few studies conducted so far to explore the effects of Spirulina-supplemented diets on the systemic antioxidant defenses of broiler chickens seem consistent with this knowledge (Table 6). More specifically, in the recent study by Abdelfatah et al. (2024) [41], supplemented chicks showed increased serum total antioxidant capacity (TAC) starting from the lowest level of supplementation tested (0.1%), as well as increased plasma activity of the antioxidant enzyme catalase (CAT) in response to the two higher supplementation levels tested (0.3 and 0.5%). In addition, in the earlier study by Park et al. (2018), a linear increase in the serum activity of the antioxidant enzymes SOD and GPx was recorded in response to increasing levels of dietary Spirulina supplementation (from 0.25 up to 1%) [63]. Further confirmatory evidence of the favorable influence exerted by dietary inclusion levels of Spirulina of 0.5% and 1% on the systemic antioxidant status of broiler chickens comes from the studies of Alaquil and Abbas (2023) and Mirzaie et al. (2018), who demonstrated that the level of oxidative stress in the supplemented chickens was reduced [58,64]. More specifically, in the study by Alaquil and Abbas (2023), chickens fed a 1% Spirulina-containing diet were found to have increased serum activity of the enzyme SOD, along with increased serum levels of the antioxidant non-enzymatic compound glutathione reduced (GSH) [64]. These changes were associated with decreased serum levels of the lipid oxidation marker malondialdehyde (MDA).

Similarly, Mirzaie et al. (2018) reported increased serum SOD activity and decreased serum levels of MDA in response to both 0.5% and 1% Spirulina-supplemented diets, as well as in response to a diet supplemented with Spirulina at 2%. At this higher level of dietary Spirulina supplementation (2%), the same authors also recorded increased serum GPx activity [58]. It is worth noting that in this latter study, the improvements in the systemic oxidant/antioxidant balance of the chickens produced by dietary Spirulina at the three different supplementation levels tested (0.5%, 1%, 2%) were not associated with any growth performance improvements (see Section 4.1.1). Similarly, in the abovementioned study by Abdelfatah et al. (2024), the lowest of the three supplementation levels tested (0.1%) proved able to improve the systemic antioxidant status of the chickens but not their productive performance [41]. So it is true, as stated by some authors, that the favorable effects of dietary Spirulina supplementation on the chickens’ antioxidant systems can be appreciated within the same range of dietary supplementation levels that can enhance growth performance [52], but it is also true that they can occur independently.

**Table 6:** Overview of the studies evaluating the effects of dietary Spirulina supplementation on the systemic antioxidant status of broiler chickens.

Spirulina Level (%)	Effects on Serum/Plasma Antioxidant Defenses	Effects on Serum/Plasma Markers of Oxidative Damage	Notes	Reference
0.1–0.3–0.5	↑ TAC ↑ CAT activity	-	The three levels were equieffective at increasing serum TAC; The lower level (0.1%) was not effective at increasing serum CAT activity; the two higher levels (0.3 and 0.5%) were equieffective at producing this effect.	[41]
0.25–0.5–0.75–1	↑ SOD activity ↑ GPx activity	-	The effects linearly increased as the inclusion level increased.	[63]
1	↑ SOD activity ↑ GSH levels	↓ MDA levels	-	[64]
0.5–1–2	↑ SOD activity ↑ GPx activity	↓ MDA levels	All of the three levels were effective at increasing serum SOD activity and decreasing MDA levels; Only the highest level (2%) was effective at increasing serum GPx activity.	[58]

The “↑ ”or “↓ ”arrows indicate that an increase or a decrease, respectively, was observed in the parameter with the experimental diet (Spirulina-containing) compared with the standard broiler diet (control). **Abbreviations**: TAC = total antioxidant capacity; CAT = catalase; SOD = superoxide dismutase; GPx = glutathione peroxidase; MDA = malondialdehyde.

***(b) Effects on Liver Health***

The reviewed literature suggests that supplementing broiler diets with Spirulina may also improve the chicken liver’s health status (Table 7). For instance, Abdelfatah et al. (2024) reported enhanced liver histomorphology in response to all Spirulina supplementation levels tested (0.1%, 0.3%, 0.5%), with reduced (or even absent) leukocyte infiltration being observed in all of the supplemented groups compared with the unsupplemented control [41]. Consistently with this finding, other investigators measured lower serum levels of the enzymes alanine aminotransferase (ALT) and/or aspartate aminotransferase (AST), i.e., increased hepatocellular integrity, in chickens fed diets supplemented with comparable (0.09, 0.1, 0.2, 0.4%) [4,59,62], as well as slightly lower (0.05–0.07%) [4] or higher (0.8%) [62] levels of Spirulina. Reasonably, it has been suggested that these results could be the outcome of the antioxidant and anti-inflammatory activities exerted by Spirulina-derived components transferred to the liver [62], with the antioxidant activity also having per se the potential to result in the mitigation of inflammation [95]. In corroboration of this hypothesis, Kasmani et al. (2023) found the liver of chickens fed a 0.1% Spirulina-supplemented diet to be characterized by improved oxidant/antioxidant balance (as indicated by decreased MDA levels and increased SOD, CAT, and GPx activities) and reduced inflammation (as indicated by reduced NO levels) [59]. However, it should be mentioned that Ibrahim et al. (2021), using a supplementation level of 2%, reported no changes in the values of the two major biochemical markers of hepatic damage (i.e., ALT and AST), as well as no changes in the liver oxidant/antioxidant balance (as assessed by measurement of tissue levels of MDA, SOD, and GSH) [67].

**Table 7:** Overview of the studies evaluating the effects of dietary Spirulina supplementation on the health status of the liver in broiler chickens.

Spirulina Level (%)	Effects on Liver Structural Integrity	Effects on Liver Oxidative/Inflammatory Status	Effects on Liver Metabolic Function	Notes	Reference
0.03–0.05–0.07–0.09	↓ Serum ALT activity ↓ Serum AST activity	-	↓ Serum total cholesterol ↓ Serum triglycerides ↔ Serum albumin ↑ Serum globulins ↔ Serum total proteins	The lowest level (0.03%) was not effective on any of the parameters. The three higher levels (0.03, 0.05, and 0.09%) were equieffective at reducing ALT, total cholesterol and triglycerides levels, as well as increasing globulin levels. Only the two higher levels (0.07 and 0.09%) were effective at reducing serum AST activity levels, and they were equieffective.	[4]
0.1	↓ Serum ALT activity ↔ Serum AST activity ↔ Serum GGT activity ↔ Serum ALP activity ↔ Serum LDH activity	↑ SOD, CAT, GPx activities ↓ MDA levels ↓ NO levels	-	-	[59]
0.1–0.3–0.5	Improved histomorphology: ↓ leukocyte infiltration	-	-	The three levels were equieffective at improving the liver histomorphology.	[41]
0.2–0.4–0.8	↓ Serum ALT activity ↓ Serum AST activity	-	-	The three levels were equieffective at reducing ALT and AST levels.	[62]
0.5–1	-	-	↑ Serum total cholesterol ↑ Serum LDL-cholesterol ↔ Serum triglycerides ↔ Serum albumin ↔ Serum globulins ↔ Serum total proteins	The higher level (1%) was less effective than the lower (0.5%) at increasing total cholesterol. The two levels were equieffective at increasing LDL-cholesterol.	[48]
0.25–0.5–1	-	-	↓ Serum total cholesterol ↔ Serum triglycerides ↔ Serum albumin ↑ Serum globulins ↑ Serum total proteins	The lowest level (0.25%) was not effective. The two higher levels (0.5 and 1%) were equieffective at producing the changes.	[5]
1	-	-	↓ Serum total cholesterol ↔ Serum albumin	-	[65]
2	↔ Serum ALT activity ↔ Serum AST activity	↔ SOD activity ↔ GSH levels ↔ MDA levels	-	-	[67]
0.5–1–2	-	-	↓ Serum total cholesterol ↓ Serum triglycerides ↓ Serum total lipids	All of the three levels were effective at reducing serum total cholesterol and total lipid levels; the effect was dose-dependent. Only the two higher levels (1 and 2%) were effective at decreasing serum triglycerides; the effect was dose-dependent.	[58]

The “↑ ”, “↓ ”, or “↔” arrows indicate that an increase, a decrease, or no change, respectively, was observed in the parameter with the experimental diet (Spirulina-containing) compared with the standard broiler diet (control). **Abbreviations**: ALT = alanine aminotransferase; AST = aspartate aminotransferase; GGT = gamma-glutamyl transferase; ALP = alkaline phosphatase; LDH = lactate dehydrogenase; LDL = low-density lipoprotein; SOD = superoxide dismutase; CAT = catalase, GPx = glutathione peroxidase; GSH = glutathione; MDA = malondialdehyde; NO = nitric oxide.

Besides structural integrity, the liver’s metabolic function also seems favorably influenced by using Spirulina as a feed supplement. In this respect, regulation of lipid metabolism is known as one of the main physiological roles of this organ, and dietary Spirulina supplementation has been quite consistently shown to exert a beneficial hypocholesterolemic effect in broiler chickens. This decrease in the serum concentrations of total cholesterol was reported for dietary Spirulina inclusion levels ranging from 0.05% to 2% [4,5,58,65], in association with either decreased [4,58] or unchanged [5] levels of triglycerides and decreased levels of total lipids [58]. The only exception in the reviewed literature is represented by the study of Alwaleed et al. (2021), who measured increased levels of cholesterol and/or increased levels of the harmful LDL-cholesterol (again with no change in the levels of triglycerides) in chickens fed 0.5% and 1% Spirulina-supplemented diets compared with the control birds [48]. Lower confidence can be placed in the latter finding, considering that dietary Spirulina exerts a well-documented hypolipidemic activity in other animal species [96,97]. This beneficial effect has been mainly attributed to the antioxidant pigment–protein complex C-phycocyanin contained in the microalga (Table 1), which would be able to inhibit the activity of pancreatic lipase enzyme, leading to reduced fat absorption in the intestinal tract [96]. Some authors have also proposed a contribution by the Spirulina-derived polyphenolic compounds [98]. The major role of C-phycocyanin in mediating the hypolipidemic effects of Spirulina in chickens seems confirmed by two recent studies, in which the direct supplementation of broiler diet with Spirulina-derived C-phycocyanin resulted in the occurrence of a distinct hypolipidemic effect [87,99]. It is worth noting that the positive influence that Spirulina-supplemented diets seem to exert on broiler chickens’ liver lipid metabolism and related blood lipid profile has the potential to translate into desirable changes in the carcass and meat composition [12,52]. Indeed, the hypocholesterolemic effect reported by Fathi et al. (2018) was associated with a decrease in the relative weight of the abdominal fat pad (see Section 4.1.1) [4].

The liver also plays a key role in synthesizing plasma proteins, including albumin, α and β globulins, and fibrinogen. With respect to this physiological function, some authors argued that an enhancing influence by dietary Spirulina might be expected in light of the high protein content of this microalga [95]. However, the same authors have acknowledged that the relationship between the innate nutritional composition of Spirulina (as of any other ingested feed ingredients or supplements) and metabolic outcomes is not always straightforward [95]. This, possibly, may at least in part explain the substantially non-confirmatory findings of the studies that have explored the effects of dietary Spirulina supplementation (at inclusion levels overall ranging from 0.03% to 2%) on the chickens’ serum levels of total proteins and/or their single fractions. In this regard, all of the studies seem to agree on the lack of changes in the serum levels of albumin [4,5,48,67]. In one of the studies, this finding was found to be similarly associated with no change in the serum levels of total globulins and, consistently, with no changes in the serum levels of total proteins [48]. In two other studies, an increase in the serum levels of total globulins was recorded [4,5]; however, only in the study by Hassan et al. (2022) was this increase in serum globulins significant enough also to determine a parallel rise in the serum total proteins [5]. Based on what will be discussed here below, this result may not only reflect increased production of α- and β-globulins by the chickens’ liver, attributable to a modulatory influence of dietary Spirulina on the metabolic activity of this organ, but also, and most likely, it may reflect an increased production of B-cell derived γ-globulins (immunoglobulins or Ig) consequent to the enhancing influence of dietary Spirulina on chicken immune function. The latter point will be discussed in the following dedicated paragraph.

***(c) Effects on Immune Health (and Protection Against Pathogen Challenges)***

The reviewed literature provides evidence that the beneficial health effects of supplementing broiler diets with Spirulina also extend to the chickens’ immune systems [2,3,12,23,73,76]. Zinc, polysaccharides, braun-type lipoproteins (immulina), *n*-3 polyunsaturated fatty acids, heptadecane (a biogenic volatile hydrocarbon), and C-phycocyanin are the functional components of Spirulina that have been indicated as responsible, at least in part and to different extents, for these beneficial immune-related effects of the microalga [23,35,87,92,93,100,101,102].

A first group of reports that can be cited in this regard documents the existence of an association between dietary intake of Spirulina (at supplementation levels ranging, on the whole, from 0.01 to 2%) and occurrence in broiler chickens of potentially favorable changes in various general immunological markers (Table 8).

For instance, as mentioned above, Hassan et al. (2022) recorded increased serum levels of globulins in chickens fed 0.5% and 1% Spirulina-supplemented diets compared with the unsupplemented chicks, and chicks receiving the lower supplementation level of 0.25% [5]. Moreover, in the same study, birds fed diets containing 0.5% Spirulina had a higher number of circulating leukocytes, and all groups of supplemented chickens (0.25, 0.5, and 1%) showed a higher percentage of lymphocytes than birds in the unsupplemented control group. Consistent with these latter findings, an increased relative weight of the lymphoid organ thymus (indicative of enhanced organ development) was also recorded. Similarly, increases in the relative weights of three major immune organs (thymus, bursa of Fabricius, and spleen), as well as improvements in the serum levels of globulins (increased), in the numbers of total circulating leukocytes and lymphocytes (increased), and in the value of the heterophil to lymphocyte (H/L) ratio (decreased) were reported by Fathi et al. (2018) for chickens receiving the relatively low levels of dietary Spirulina supplementation of 0.07 and 0.09% [4]. Other authors provided further confirmatory evidence for the presence of a higher number of leukocytes in chickens fed diets supplemented with levels of Spirulina ranging on the whole from 0.2% to 1% [62,64]. In contrast, Abdelfatah et al. (2024) provided further confirmatory evidence that the chickens receiving diets supplemented with 0.5% Spirulina had a greater bursa relative weight [41].

Moreover, Abdelfatah et al. (2024) found that all three major immune organs (bursa, thymus, and spleen) had a better histological structure in the birds that received Spirulina than the unsupplemented ones [41]. In the study by Khadanga et al. (2023), 0.5% dietary Spirulina proved unable to improve the development of lymphoid organs [66]. However, increased relative weights of the thymus, bursa, and spleen were documented for levels of dietary Spirulina supplementation of 1% and 1.5%. In the study by Qureshi et al. (1996), broilers receiving diets supplemented with levels of Spirulina ranging from a minimum of 0.01% to a maximum of 1% were found to have enhanced macrophage phagocytic function [57]. Al-Batshan et al. (2001) confirmed this finding for levels of dietary Spirulina supplementation of 0.5, 1, and 2.0%, observing dose-dependency in producing this effect [103].

**Table 8:** Overview of the studies evaluating the effects of dietary Spirulina supplementation on the health of the immune system of broiler chickens as assessed by general immunological markers.

Spirulina Level (%)	General Immunological Markers Undergoing Changes in Response to Spirulina				Notes	Reference
	Serum Immune-Related Proteins	Blood Immune-Related Cells	Immune Organs	Humoral and Cellular Functional Responses to Stimulation		
0.03–0.05–0.07–0.09	↑ Serum globulins	↑ WBC count ↑ Lymphocyte count ↓ Heterophil count ↓ H/L ratio	↑ r.w. of thymus, bursa, spleen	-	The lowest level (0.03%) and the two highest levels (0.07 and 0.09%) were equieffective at decreasing the heterophil count. The three higher levels (0.05, 0.07, and 0.09%) were equieffective at increasing serum globulins. Only the two higher levels (0.07 and 0.09%) were effective at increasing WBC count, lymphocyte count, and thymus and spleen r.w., as well as at decreasing H/L ratio, and were equieffective. Only the highest level (0.09%) was effective at increasing bursa r.w.	[4]
0.1–0.3–0.5	-	-	↑ r.w. of bursa Improved histomorphology of thymus, bursa, spleen	-	Only the highest level (0.5%) was effective at increasing the bursa r.w. None of the levels caused changes in the spleen r.w. The three levels were equieffective at improving the immune organ histomorphology.	[41]
0.2–0.4–0.8	-	↑ WBC count	-	-	The three levels were equieffective at increasing WBC count.	[62]
0.25–0.5–1	↑ Serum globulins	↑ WBC count ↑ Lymphocyte %	↑ r.w. of thymus	-	The three levels were equieffective at increasing the lymphocyte %. Only the intermediate level (0. 5%) was effective at increasing the WBC count and thymus r.w. The two higher levels (0.5 and 1%) were equieffective in producing the increase in serum globulins.	[5]
0.01–0.1–1	-	-	-	↑ Macrophage phagocytic function ↑ Ab titers against SRBC antigens	None of the levels caused changes in spleen and bursa r.w. None of the levels proved effective at increasing the lymphoproliferative response to PHA-P. The three levels were equieffective at enhancing the macrophage phagocytic function. Only the highest level (1%) was effective at boosting the humoral response to SRBC antigens.	[57]
0.01–0.1–1	↑ Serum IgG levels	-	-	↑ Monocyte phagocytic function ↑ Ab titers against SRBC antigens ↑ Ab titers against BA antigens	None of the levels proved effective at increasing serum IgA levels. The three levels were equieffective at enhancing the monocyte phagocytic function. The two higher levels (0.1 and 1%) were equieffective at increasing serum IgG levels at 5 weeks of age; only the 0.1% level was effective at increasing serum IgG levels at 7 weeks of age. Only the 0.1% level proved effective at increasing the Ab response to SRBC antigens (only at 6 weeks of age). Only the 0.01% level proved effective at increasing the Ab response to BA antigens (only at 6 weeks of age). Heteroscedasticity occurred in the group receiving the highest level (1%).	[104]
1	-	↑ WBC count	-	↑ Ab titers against SRBC antigens ↑ Lymphoproliferative response to Con-A (T cells) ↑ Lymphoproliferative response to LPS (B cells)	No change occurred in the H/L ratio.	[64]
0.5–1–1.5	-	-	↑ r.w. of thymus, bursa, spleen	↑ Lymphoproliferative response to PHA-P	The lowest level (0.5%) was not effective. The two higher levels (1 and 1.5%) were equieffective at increasing the r.w. of spleen, whereas 1.5% was more effective than 1% at increasing the r.w. of thymus Only the highest level (1.5%) was effective at increasing the r.w. of bursa and cellular response to PHA-P.	[66]
0.5–1–2	-	-	-	↑ Macrophage phagocytic function	The effect was dose-dependent.	[103]
0.5–1–2	-	-	-	↑ Ab titers against SRBC antigens	Only the lowest level (0.5%) was effective at producing the effect.	[58]

The “↑ ”or “↓ ”arrows indicate that an increase or a decrease, respectively, was observed in the parameter with the experimental diet (Spirulina-containing) compared with the standard broiler diet (control). Where two or more levels of Spirulina supplementation were tested, and only some proved effective, the effective ones appear underlined. **Abbreviations**: WBC = white blood cell; H/L = heterophil to lymphocyte ratio; r.w. = relative weight (% of the pre-slaughter weight); Ab = antibodies; Ig = immunoglobulin; SRBC = sheep red blood cell; BA = Brucella abortus; Con-A = concanavalin-A; LPS = lipopolysaccharide; PHA-P = phytoheamagglutinin-P.

Similarly, Katayama et al. (2016) documented increased phagocytic capacity of blood monocytes in broilers fed diets supplemented with 1% Spirulina, as well as in broilers receiving 0.1% and 0.01% Spirulina-supplemented diets [104]. At the two higher supplementation levels (0.1 and 1%), these authors also observed significantly increased systemic capacity of antibody (Ab) production, as indicated by the measurement of higher serum levels of IgG. Moreover, in the same study, broilers receiving 0.1% Spirulina also showed significantly higher Ab titers against sheep red blood cell (SRBC) antigens. In contrast, significantly higher Ab titers against *Brucella abortus* antigens were measured in broilers receiving Spirulina at an inclusion level as low as 0.01%. Other studies provided further evidence of enhanced humoral response to SRBC antigens [57,58,64]. Moreover, broiler chickens fed Spirulina-supplemented diets have been shown to have higher lymphoproliferative responses to specific mitogens (e.g., phytohemagglutinin-P, concanavalin-A) or other suitable stimuli (e.g., bacterial lipopolysaccharide) [64,66].

Together, these findings suggest the ability of feed supplementation with this microalga to promote more efficient innate and adaptive immune responses in broiler chickens. This immune-enhancing activity, in turn, can increase the birds’ disease resistance potential [57,103] and reduce mortality rates and medication use. Some studies can be found in the reviewed literature to confirm the actual achievement of this positive outcome. For instance, Kaoud (2015) reported decreased mortality in broilers fed a 0.1% Spirulina-supplemented diet [50]. In addition, there are the studies by Lokapirnasari et al. (2016) and Kumari et al. (2019) that document the ability of dietary Spirulina supplementation to aid the immune system of the chickens in coping with infections caused by either field or vaccine viruses [105,106]. More specifically, in the study by Lokapirnasari et al. (2016), the administration of Spirulina to broiler chickens (added at 20% concentration in freshwater as a liquid supplement from day 7 to day 32 of age) during an experimental challenge with the H5N1 avian influenza (AI) virus was found to significantly increase the total number of leukocytes (compared with the non-supplemented birds), and this was associated with significantly decreased mortality [105]. In the study by Kumari et al. (2019), Spirulina supplementation at 1% in feed (from 10 to 20 days of age) was evaluated for its efficacy in counteracting the immunosuppressive effect of live commercially available hot strains of vaccines for infectious bursal disease (IBD), using the concentration of total proteins measured in serum as a marker of the immune system health [106]. The results showed that the unsupplemented IBD-vaccinated chickens had significantly lower serum total proteins than the control non-vaccinated birds. In contrast, Spirulina-fed IBD-vaccinated chickens showed serum total protein concentrations almost equal to those measured in control non-vaccinated chickens. Therefore, dietary Spirulina supplementation at the level of 1% was suggested as a valid strategy, in combination with the live IBD vaccine, to reduce the negative immunosuppressive effect of the latter [106].

It is also worth mentioning the study by Alaqil and Abbas (2023), who reported that broilers fed 1% Spirulina-supplemented diets were more resistant than the unsupplemented ones to an experimentally induced *E. coli* infection, showing partial to complete mitigation (depending on the parameter examined) of the adverse effects that the bacterial challenge had on the birds’ growth performances, gut health statuses, oxidant/antioxidant balances, and immune functions [64]. Further evidence of Spirulina-induced increase in the chickens’ resistance to bacterial infections comes from the study of Atiyah and Hamood (2021) [68]. Interestingly, in this case, Spirulina was mixed with the drinking water at a final concentration of 2% and proved more effective than oxytetracycline (similarly administered via the drinking water) at ensuring mitigation of the harmful changes produced by *Enterococcus faecalis* infection in the values of most growth performance parameters (final BW, BWG, and FI) [68]. With particular regard to FCR, which was not affected by the infectious condition, an improvement was recorded under the influence of Spirulina supplementation compared with the value calculated for the healthy unsupplemented broilers, whereas this did not occur under the influence of oxytetracycline [68].

However, the interaction between the intake of Spirulina as a feed supplement and the functionality of the immune system of the chickens is more complex and less predictable than it would appear from these data. Such complexity emerges from the abovementioned study of Katayama et al. (2016) [104] (Table 8). Indeed, these authors found that, unlike IgG production, the production of IgA was not influenced by dietary Spirulina supplementation, and this was suggested to be because dietary Spirulina would interact only with the regulatory mechanisms of IgG production and not with those involved in IgA production. In addition, the same authors observed that among the broilers receiving the higher dietary supplementation level of Spirulina (1%), there was a large proportion of individuals in which some, at least, of the positive immune-related effects (increased IgG production and specific antibody responses to the selected test antigens), did not occur at all. One proposed explanation for this lack of response was the concomitant activation of immunosuppressive mechanisms, such as those involving regulatory T-cells. So, according to this intricate scenario, supplementing the broiler diet with Spirulina would positively influence only some aspects of chickens’ immunological responses, while others would remain unaffected, or may even be negatively affected. Furthermore, for the positive immune-related effects of Spirulina to be obtained, appropriate supplementation levels are required (potentially different depending on the specific immune function to be stimulated), with too high dosages potentially leading to effect saturation or even inhibiting the occurrence of the desired effect [52].

Further elements of complexity emerge from the analysis of a second group of reports identified in the reviewed literature. These reports explored whether dietary Spirulina supplementation, by virtue of its documented immune-enhancing properties, could be used as a natural immunostimulant to increase the broiler chickens’ responses to vaccination against field-relevant pathogens and help them achieve the highest possible level of protection against superinfections potentially occurring after vaccination (Table 9). The first study that can be mentioned in this respect is the one conducted by Awad et al. (2023), who investigated the efficacy of dietary Spirulina supplementation (at 0.1, 0.3, and 0.5%) in improving the specific humoral immune response of broiler chickens to three distinct vaccines (the vaccine against the H5N1 AI virus, the vaccine against infectious bronchitis—IB virus, and the vaccine against the Newcastle disease—ND virus), as well as at protecting ND-vaccinated birds against the negative impact of an infectious challenge with a heterologous virulent ND virus (genotype VII) [107]. In line with the intricate scenario depicted above, the addition of Spirulina to broilers’ diets was able to increase the response to the vaccine against the H5N1 AI virus (though significantly higher specific Ab titers than in vaccinated non-supplemented birds were measured only in birds supplemented with the 0.3% dose); however, this immune-stimulating effect was not exerted on the response to the other two vaccines. Instead, all doses of Spirulina resulted in lower Ab titers against the IB virus than those measured in vaccinated non-supplemented birds (with the most pronounced inhibitory effect on this specific immune response being exerted by Spirulina at the lowest supplementation level of 0.1%); moreover, the highest dose of Spirulina (0.5%) was associated with significantly or numerically lower Ab titers against the ND virus (at 20 and 29 days of age, respectively) compared with vaccinated unsupplemented birds. Spirulina supplementation also failed to boost the humoral immune response to the ND virus when the ND-vaccinated birds were exposed to the virulent challenge. Indeed, these animals showed post-challenge Ab titers that were not different from those measured in the unsupplemented vaccinated and challenged birds. Nevertheless, dietary Spirulina supplementation was found to provide adequate additional protection against the negative impact of the virulent challenge on chickens’ health, leading—in comparison with the vaccine alone—to a further decrease in morbidity and mortality rates (regardless of the inclusion level), as well as to further mitigation of pathological lesions (regardless of the inclusion level). Interestingly, these protective effects were also associated with a greater decrease in viral shedding titers (with the maximum inhibitory effect on virus replication being recorded with 0.5% of Spirulina), as reported in an earlier study by other authors [108].

These findings led the authors to hypothesize that the mechanisms implicated in this protection by Spirulina could include enhancement of cellular (rather than humoral) immunity (e.g., through improved proliferation of specific T-lymphocyte populations, up-regulated production of specific cytokines in mononuclear cells, enhanced activity of natural killer cells and macrophages), along with exertion of anti-inflammatory activity and direct antiviral activity against the challenging ND virus, with the latter activity being most likely attributable to the polysaccharide calcium spirulan contained in Spirulina [3,37]. Based on their findings, Awad et al. (2023) suggested that, in addition to the vaccination program, Spirulina supplementation (particularly at a level of 0.3% in diet) could improve the clinical protection against ND outbreaks and decrease further viral transmission into the field [107]. Moreover, although not highlighted by the authors, this nutritional strategy might increase the protective potential of the vaccination against the H5N1 AI virus but also have potentially negative implications for the protective potential of the vaccination against the IB virus, which would require further evaluation.

In partial agreement with Awad et al. (2023), other authors reported no positive influence of Spirulina on the ND virus Ab titers in broiler chickens [4]. However, neither of these same authors observed significant effects of Spirulina on Ab titers against the AI virus. Considering that both studies used Cobb chickens, Awad et al. (2023) identified as a potential explanation for this discrepancy the lower supplementation levels of Spirulina tested by Fathi et al., 2018, which were below 0.1% (0.03–0.09%), and probably too low in relation to the sensitivity of the parameters examined [4,107]. The influence of other differences in the experimental conditions adopted in the two studies (including the vaccine type and/or vaccination program) cannot be ruled out. In contrast with Awad et al. (2023), Khan et al. (2020) and Abotaleb et al. (2020) reported that feed supplementation with 0.1, 0.15, and 0.2% of Spirulina significantly improved the chickens’ responses to the vaccine against the ND virus, resulting in higher Ab titers than in birds of the unsupplemented group [45,107,108].

Moreover, in the study by Kasmani et al. (2023), dietary supplementation with 0.1% Spirulina increased humoral responses to the vaccinal ND virus and the vaccinal IB and AI viruses [59]. Finally, in the study by Khadanga et al. (2023), a positive influence of dietary Spirulina on the chickens’ responses to the anti-ND virus vaccine was documented for supplementation levels of 1% and 1.5% [66]. For all these studies, a possible contribution to obtaining an outcome different from that reported by Awad et al. (2023) may also derive from the use of different bird strains (Table 9) [107].

Adopting an experimental design similar to that of Awad et al. (2023) (i.e., vaccination followed by viral challenge) but focusing on a different virus (H9N2 AI virus), Yehia et al. (2024) provided further evidence in support of the particular usefulness of dietary Spirulina supplementation in increasing the protective potential of vaccination (or, at least, of some vaccinations) in broiler chickens, as well as evidence for the complexity of the interaction between Spirulina and chicken immunity [109]. More specifically, the authors observed that birds receiving feed supplemented with an in-house prepared Spirulina extract at low (200 mg/kg feed) and high (400 mg/kg feed) concentrations produced significantly higher Ab titers against the H9N2 AI vaccine in comparison with unsupplemented controls, and this effect was dose-dependent. Moreover, supplemented vaccinated birds were found to also dose-dependently produce higher specific Ab titers and develop less marked histopathological changes after a challenge with a circulating strain of the H9N2 AI virus. These findings accounted for the authors’ recommendation to use 400 mg of Spirulina extract/kg feed in combination with the vaccine against the H9N2 AI virus to ensure the maximum possible protection.

Besides the specific humoral immune response to the selected pathogen (as assessed through Ab titers), various other aspects of immune-related functions were analyzed in this study and were dose-dependently enhanced by the intake of the Spirulina extract. In particular, a greatly enhanced phagocytic activity of peripheral blood monocytes, along with increased serum lysozyme levels, were recorded in the supplemented chicks compared with the unsupplemented ones [109]. Both these effects were detectable during the post-vaccination and post-challenge periods and were indicative of an improved innate immune response [110], plausibly aided in counteracting the well-known immune-suppressive effects of AI virus infection [109]. Moreover, besides demonstrating the occurrence of the immunostimulatory effects just described, the study by Yehia et al. (2024) revealed that feed supplementation with Spirulina extract at both concentrations tested (200 and 400 mg/kg of feed), was able to also protect the chickens against the inflammation associated with H9N2 AI virus infection [109]. This anti-inflammatory activity was indicated by the measurement of lower serum levels of the pro-inflammatory mediator nitric oxide in the supplemented chickens, compared with the unsupplemented ones, after the viral challenge.

Moreover, lymphoid organs, such as the spleen and bursa, documented decreased inflammation and overall mitigation of histopathological alterations. The decreased nitric oxide levels were interpreted as the likely consequence of down-regulated expression of the iNOS enzyme, given the presence, among the bioactive Spirulina components, of substances like heptadecane and C-phycocyanin that are capable of inhibiting iNOS gene expression via inhibitory modulation of the nuclear factor kappa B (NF-kB) transcription factor pathway [111,112,113,114,115]. C-phycocyanin has been reported to also act as direct inhibitor of the activity of iNOs, as well as of the inducible pro-inflammatory enzyme cyclooxygenase 2 [2]. However, the participation of this mechanism in the anti-inflammatory effects of dietary Spirulina supplementation in broiler chickens has not yet been explored. On the other hand, from research specifically conducted in broiler chickens, evidence has been provided that C-phycocyanin, administered as a feed supplement, can reduce the immunoexpression of the pro-inflammatory cytokine tumor necrosis factor α [87], and this event also may contribute to the overall anti-inflammatory activity of Spirulina-supplemented diets.

In light of the multifaceted immune-related events that authors like Katayama et al. (2016), Awad et al. (2023), and Yehia et al. (2024) documented (or hypothesized) to occur in broiler chickens fed Spirulina-supplemented diets [104,107,109], the beneficial influence that Spirulina seems to exert on the immune system of these birds would be more appropriately definable as “immunomodulatory” (or “immunoregulatory”), rather than merely “immunostimulatory” [2]. By activating some pathways of the innate and adaptive immunity and concomitant inhibition of others, Spirulina as a feed supplement would contribute to maintaining balanced and, hence, efficient inflammatory and immune responses. This favorable immunomodulatory activity, combined with concomitant exertion of direct antiviral and antibacterial activities [2] (which are still under-explored in vivo), makes the dietary supplementation of Spirulina a rather promising nutritional strategy to aid in the protection of broiler chickens against infectious diseases, but more in-field research is needed to prove more robustly that these advantages can be achieved.

#### 4.1.3. Effects on the Productive Performance and Health Status (Intestinal and/or Extra-Intestinal) of Broiler Chickens Exposed to Physical and Chemical Challenges

Besides its potential to offer protection against pathogen challenges, dietary Spirulina supplementation has also been tested for its ability to protect broiler chickens against challenges of physical and chemical natures.

***(a) Protection against Physical Challenges (Heat Stress)***

Many studies in the reviewed literature consistently report that supplementing broiler diets with Spirulina may be a valid nutritional strategy to support the productive performance and health of the chickens under heat-stress conditions (Table 10). It is of note that the general agreement in the formulation of this conclusion comes despite apparent heterogeneity in how the feeding trials have been designed and conducted by the different authors and even by the same groups of investigators [54,91,116], with inter-study differences involving, among others, type and composition of the Spirulina product used as a supplement, mode of administration of the supplement, time frame and duration of the feeding period, composition of the basal diet supplemented with Spirulina, protocol of heat-stress induction, genetics, and age of the animals subjected to heat-stress and receiving dietary Spirulina supplementation.

It is well known that rearing broiler chickens under high environmental temperature induces oxidative stress and triggers inflammatory processes (partly consequent to the oxidative damage of cells, tissues, and organs) [117,118]. This, in turn, causes alterations in the structural and functional integrity of many physiological systems, including the immune system, the liver, the kidneys, and the intestine, ultimately leading to increased susceptibility to infectious diseases and compromised growth performance as compared with broilers reared under thermoneutral conditions [90,116,117,118,119,120,121]. The well-documented antioxidant, anti-inflammatory, immunomodulatory, hypolipidemic, and prebiotic properties of specific components of the microalga Spirulina (C-phycocyanin, *n*-3 polyunsaturated fatty acids, phenols, polysaccharides, and others) seem particularly relevant to this challenging context, and likely account for the positive outcomes reported in the literature [35,96,122].

Two of the most valuable studies that can be cited in this regard are those from Attia et al. (2023) [117] and Moustafa et al. (2021) [118]. In the former study, a diet supplemented with 0.1% Spirulina was found effective at mitigating all of the adverse effects that a 2-week exposure to cyclic heat stress (35 ± 1 °C for 9 h/day, from day 22 to day 35 of age) had on various indices related to growth performance (final BW, FCR, EPEI), immune function (lymphoid organ relative weights; IgM and IgY levels; Ab titers against vaccine viruses; leukocyte count, percentage of heterophils, percentage of lymphocytes, and H/L ratio), intestinal health (counts of Lactobacilli and Coliforms; villus and crypt morphometry), liver health (ALT and AST serum activities, serum levels of total cholesterol, LDL-cholesterol and triglycerides), kidney function (serum levels of creatinine and uric acid), and systemic antioxidant/oxidant balance (serum levels of the lipid peroxidation marker MDA and serum total antioxidant capacity—TAC) (Table 10) [117]. Depending on the specific parameter examined, the protection exerted by the Spirulina-supplemented diet in heat-stressed (HS) broilers was found to be either partial (i.e., leading to values that were improved when compared with the HS-positive control group fed an unsupplemented diet, but still impaired when compared with the non-HS negative control group reared under thermoneutral conditions and fed a basal unsupplemented diet), or complete (i.e., leading to values not different from those recorded in the non-HS and unsupplemented negative control group). Moreover, improvements over the negative control were reported for some of the parameters affected by heat stress (particularly, cecal Lactobacilli count and serum uric acid levels). It is worth noting that, consistent with these effects, Attia et al. (2023) also recorded a decrease in the mortality rate (i.e., substantial mitigation of the heat-stress-induced increase in mortality rate), as well as in the stress indices (mainly, complete mitigation of the heat-stress-induced increase in serum corticosterone levels) for the HS chickens fed 0.1% Spirulina-supplemented diet [117]. Another interesting finding of this study was that simultaneous diet supplementation with Spirulina and garlic powder resulted in synergistic effects, leading to more effective protection against the detrimental impact of heat stress.

As for the study by Moustafa et al. (2021), these authors obtained almost complete protection against the adverse effects of a similar condition of cyclic heat stress (34 ± 1 °C for 8 h/day, from day 21 to day 42 of age) by supplementing the chickens’ diets with higher levels of Spirulina [118]. More specifically, three supplementation levels were tested in this study, namely 0.5%, 1%, and 1.5%. Of these, 1% was the most effective at counteracting the heat-stress-induced impairment of growth performance (final BW, BWG, FCR, European broiler index) and some carcass parameters (dressing percentage and breast yield). For most of the other indices affected by heat stress, the three supplementation levels proved equieffective, or the two higher levels (1 and 1.5%) more effective than the lower (0.5%) at ensuring their maintenance at almost normal values (i.e., not different from or very close to negative control values). This mainly happened for fat relative weight, serum TAC, MDA, GSH, and AST activity levels, total cholesterol, triglycerides, and creatinine. For some other heat-affected parameters related to the carcass (intestine relative weight), systemic antioxidant/oxidant balance (serum SOD activity), and liver and kidney health (LDL-cholesterol and urea), the three supplementation levels produced an improvement over the negative control [118].

Another group of investigators tested the protective potential of dietary Spirulina supplementation against the detrimental impact of experimentally induced heat stress (32–33 °C for 10 h/day, from day 22 to day 35 of age) at an inclusion level as high as 3% [70]. At the end of the challenging period, many of the growth performance indices that were negatively affected by heat stress (BW, average daily BWG, and FI) resulted in improvements in Spirulina-supplemented HS broilers relative to the non-supplemented HS broilers (positive control), with some indices (BW) reaching values not different from those recorded in the non-HS animals (negative control). Interestingly, in this case, improvement over the negative control was recorded only for those performance parameters unaffected by heat stress (FCR). A similar response pattern was also documented for the effects of dietary Spirulina supplementation on the other classes of parameters examined in the HS broilers, mostly related to gut health. More specifically, for ileal histomorphometry, partial mitigation (i.e., “relative” improvement) of the adverse effects of heat stress on ileal crypt depth and villus height to crypt depth ratio, and “absolute” improvement of the heat-unaffected intestinal villus height compared with non-HS birds were found. As for intestinal (ileal) gene expression, the Spirulina-supplemented HS broilers showed complete mitigation of the heat-stress-induced up-regulated expression of the heat shock *HSF2* gene and the immune-related *IL12* gene while showing increased expressions of the heat-unaffected *GPX3* (antioxidant related), *IL4* (immune-related), and *CLDN2* (tight-junction related) genes as compared with the non-HS broilers. Spirulina supplementation was found to also mitigate the negative impact of heat stress on the cecal microbiota. Indeed, microbial alpha and beta diversities were higher in the supplemented HS group than in the unsupplemented HS group. In particular, an increase in volatile fatty acid-producing bacteria from genera *Ruminococcus*, *Ocillospira*, *Lactobacillus*, *Oscillobacter*, *Flavonifractor,* and *Colidextribacter* was recorded in the Spirulina-supplemented HS birds.

The coexistence of “relative” and “absolute” improvements in HS broilers fed Spirulina-supplemented diets was also documented in the study by Mirzaie et al. (2018) [58]. In this case, the chickens were subjected to an acute heat challenge (36 for 6 h/day, from day 38 to day 44 of age) while receiving diets supplemented with 0.5%, 1%, or 2% of Spirulina. Heat stress significantly impacted the systemic antioxidant/oxidant balance of broiler chickens (increased serum levels of MDA, decreased serum activities of SOD and GPx). Dietary Spirulina ensured partial to complete mitigation of this effect (depending on the parameter considered) when used at the highest inclusion level of 2%. Heat stress also negatively affected some chickens’ lipid metabolism indices, leading to increased total lipids and triglycerides. In this respect, the lowest level of dietary Spirulina (0.5%) ensured complete mitigation of the lipemic boost (as indicated by a serum lipid profile not different from that of the negative control birds).

In contrast, both of the two higher levels of dietary Spirulina (1% and 2%) improved the serum lipid profile of the HS birds in comparison with the non-HS (negative control) birds, and also with respect to lipid indices that had not been altered by heat stress (total cholesterol). Similarly, heat stress resulted in an unfavorable increase in the H/L ratio (likely reflecting the lymphopenia induced by increased corticosterone levels). This effect was mitigated by 1% Spirulina, while 0.5% and 2% Spirulina resulted in H/L ratio values lower than those measured in the non-HS birds. The chickens’ growth performances and other immune-related parameters (particularly the humoral response to SRBC antigens) were not affected significantly by the heat challenge in this study (possibly because of its short duration). In this case, dietary Spirulina supplementation did not cause any “absolute” improvement in the heat-stress unaffected growth indices (just like it did not improve the growth performance during the pre-challenge phase, from day 17 to day 38 of age; see Section 4.1.1). However, it caused “absolute” improvement of the humoral immune response, as indicated by the higher Ab titers measured in the HS chickens fed the 1% or 2% Spirulina-supplemented diet compared with the non-HS unsupplemented birds (negative control).

Overall, these studies indicate that the growth- and health-enhancing effects of dietary Spirulina supplementation that are usually recorded in healthy (non-HS) broiler chickens might also occur in stressed (HS) ones. Possibly depending, at least in part, on how the specific supplementation level combines with the specific heat-stress condition, these Spirulina-induced “absolute” improvements may involve not only the physiological parameters that are not affected by heat stress but also the affected ones.

Further evidence in support of the ability of dietary Spirulina supplementation to counteract the detrimental impact of heat stress on broiler chickens’ growth performances, carcass performances, systemic antioxidant/oxidant balances, humoral immunities, lipid metabolisms, and intestinal microbiota compositions is provided by the studies performed by another group of investigators [54,91,116] (Table 10). In these studies, the improvements associated with feeding Spirulina-supplemented diets were recorded at levels of supplementation ranging, on the whole, from 0.1% [54] to 1% [54,91,116]. Unfortunately, none of the studies included a negative control group in their experimental design, which does not allow us to assess if the observed “improvements” were expressions of “partial” or complete” mitigation nor to identify the possible occurrence of “absolute” improvements. However, two interesting findings in these reports are worthy of mention. First, the research published by Abdel-Moneim et al. (2022) showed that Spirulina, in exerting its positive mitigating effects, would synergize with selenium nanoparticles so that combining the two dietary supplements in the diet of HS broilers might be recommendable to obtain even better results (especially on growth performance parameters) [91,116]. Secondly, the research published by Elbaz et al. (2022) included a valuable comparison between two different forms and modes of administration of Spirulina as a supplement [54]. More specifically, Spirulina in the dried powder form was added to the feed at the final level of 0.1% or 0.2%, as compared with Spirulina in the form of an aqueous extract, added to the drinking water at the final concentration of 0.1% or 0.2%. The study revealed that the powder form of Spirulina incorporated in the feed was superior to the aqueous extract administered via drinking water, which improved the parameters examined in the HS chickens. The lower ameliorating efficacy of the liquid extract was attributed to the fact that, during the extraction process, some of the beneficial components of the microalga Spirulina were lost, leading to lower contents of total phenols and flavonoids and a lower total antioxidant capacity in the extract compared with the dried powder.

Nevertheless, based on the findings of Kolluri et al. (2022), drinking water might remain a valid option for administering Spirulina to HS broilers and obtaining satisfactory outcomes [55]. More specifically, these authors prepared a uniform suspension of Spirulina dried powder in the drinking water at final concentrations of 0.5%, 1%, 1.5%, and 2% and administered it daily to the broilers (for 6 h in the morning) during the whole of the hot, humid summer season (mid-April to May, i.e., under natural conditions of high environmental temperature). Under these conditions, “relative” improvements of some growth performance indices (final BW and BWG) were recorded only in HS chickens that received the lower levels of Spirulina supplementation (i.e., 0.5% and 1%, with 0.5% resulting in better improvements than 1%). At higher levels of Spirulina supplementation (1.5% and 2%), growth performance remained unchanged in comparison with the unsupplemented HS birds, whereas “relative” improvements of health-related parameters were observed (particularly, serum biochemical indices of hepatic function and integrity, and immunological indices) and appeared maximized when compared with improvements produced in the same parameters by 1% Spirulina, while 0.5% Spirulina did not exert any detectable positive influence on the chickens’ health. Based on these results, the authors indicated supplementation of drinking water with Spirulina powder at a 1.5% or 2% final concentration as a recommendable strategy to improve health-related indices of broilers reared under high environmental temperature conditions, with no improvement or compromise of productive performance. Once again, the study design lacked a negative control group, so it is not possible to assess whether the observed improvements were “just” the expression of partial or complete mitigation of the negative influence exerted by heat stress or could also have been appreciated in comparison with unsupplemented birds reared under thermoneutral conditions.

***(b) Protection Against Chemical Challenges (Aflatoxins, Heavy Metals, Pesticides)***

Less numerous studies in the reviewed literature explored the protective potential of dietary Spirulina supplementation against harmful chemical contaminants to which broiler chickens may be exposed (Table 11). In this context, too, the results, although limited, seem rather encouraging.

The first group of reports that can be cited in this regard tested the feed supplement Spirulina for its efficacy in mitigating the deleterious effects of aflatoxin B_1_ (AFB_1_) in broiler chickens [59,123,124]. In the feeding trial by Raju et al. (2005), which was conducted from day 8 to day 42 of age, it was found that including Spirulina in broiler diets at 0.05% was able to alleviate only part of the adverse effects of feed contamination by 300 ppb of AFB_1_ [123]. Notably, the AFB_1_-induced decreases in BWG and relative weights of the immune organs thymus and spleen were mitigated, whereas all of the other alterations caused by AFB_1_ (including decreased FI, increased liver and kidney relative weights, decreased serum total proteins, alterations in the serum lipid profile) were not counteracted by this relatively low level of feed supplementation with Spirulina. However, in the more recent study by Kasmani et al. (2023), a two-fold higher level of Spirulina (0.1%) in the diet proved effective at ensuring complete mitigation of all of the adverse effects induced by a pretty high feed contaminating level of AFB_1_ (2.5 ppm), leading to growth performance, liver health status, and humoral immune response efficiency to vaccines that were not different from those recorded in the control chickens (i.e., in chickens fed unsupplemented and non-contaminated diets) (Table 11) [59]. Moreover, always compared with the latter, the chickens receiving the Spirulina-supplemented AFB_1_-contaminated diet even showed improvements in the composition of the intestinal microbial population (with higher counts of Lactobacilli and lower counts of Coliforms) [59]. Similar positive outcomes were reported by Feshanghchi et al. (2022), who administered a diet contaminated by 600 ppb of AFB_1_ and supplemented with 1% Spirulina (1%) to broiler chickens from day 1 to day 42 of age [124]. In this case, no improvements in the healthy control were observed. Still, all of the adverse effects of AFB_1_ on growth indices, carcass traits, liver health, immune function, and intestinal microbiota composition were mitigated entirely, except for the decrease in BWG, which was substantially but not wholly counteracted (Table 11).

In interpreting this variability in the extent to which different dietary supplementation levels of Spirulina succeed in protecting chickens against the toxicity of various levels of AFB_1_, it must be considered that just like the chickens’ responses to a certain amount of Spirulina, also the chickens’ responses to a specific dose of the mycotoxin is under the influence of factors that may increase or reduce its intensity, including, among others, the source of aflatoxin and the composition of the basal diet [125].

As for the mechanisms that may explain the protective efficacy of Spirulina against the toxic effects induced by AFB_1_ in chickens, a significant contribution likely derives from the multiple biological properties of the microalga (antioxidant, antiapoptotic, immunomodulatory, hepatoprotective, prebiotic) [126]. Moreover, Spirulina has been found to act as an aflatoxin-binder, reducing mycotoxin absorption in the gastrointestinal tract [126]. In addition, as highlighted by some authors, the vitamins contained in Spirulina play an important detoxifying role. Indeed, it has been reported that different forms of vitamin A would inhibit the formation of the AFB_1_–DNA adduct (8-hydroxydeoxy-guanosine) through the regulation of AFB_1_ metabolism by the cytochrome P450 (CYP450) enzyme system [124,127]. Ascorbic acid, of which Spirulina is also rich, would protect the organism from the acute toxicity of AFB_1_ by activating AFB_1_-epoxide hydroxylase, aldehyde reductase, and enterocyte CYP3A enzymes [127]. The pigments in Spirulina, particularly chlorophylls, may provide further protection, as they have been shown to protect against AFB_1_ carcinogenesis in the liver and colon of rats [128].

Other studies suggest that supplementing broiler diets with Spirulina may be a valid strategy to protect broiler chickens from the toxicity of heavy metals (Table 11). In this regard, very low levels of dietary Spirulina supplementation (0.005–0.02%) have been reported to exert dose-dependent mitigation of the adverse effects induced by arsenic on chickens’ growth performances and liver integrity [129]. In addition, Bharavi et al. (2021) demonstrated that the inclusion of Spirulina at the rate of 0.1% in cadmium-contaminated feed was able to mitigate the metal-induced alterations in the antioxidant/oxidant balance of the liver and kidneys, as well as in the serum markers of liver and kidney function, ensuring almost normal growth performance [130]. In all likelihood, the antioxidant and anti-inflammatory properties of Spirulina chemical components (Table 1) played a crucial role in determining the protective efficacy of dietary Spirulina against the hepatorenal damage and dysfunction caused by cadmium toxicity (which is known to be in large part due to oxidative stress) [30,35]. Furthermore, the authors suggested the contribution of another possible mechanism to hepatorenal tissue protection by Spirulina. Notably, it was hypothesized that some Spirulina components, besides acting as free radical scavengers, also acted as chelators of cadmium, reducing its progressive accumulation in chickens’ livers and kidneys and facilitating its elimination [130]. The metal-chelating ability of some antioxidant components of Spirulina (flavonoids, phycocyanin) is well documented [131,132], and the hypothesis formulated by Bharavi et al. (2021) was supported by the finding of lower cadmium concentrations in the organs of the Spirulina-supplemented intoxicated chickens, compared with the concentrations measured in the organs of unsupplemented intoxicated birds [130].

**Table 11:** Overview of the studies evaluating the effects of dietary Spirulina supplementation on the productive performance and health status of broiler chickens exposed to chemical challenges.

Spirulina Level (%)	Chemical Challenge	Challenge-Affected Parameters Showing Improvement Relative to the Positive Control ^(1)^			Notes	Reference
		Partial Mitigation of	Substantial (=Almost Complete) Mitigation of	Complete Mitigation of		
0.05	AFB_1_ (300 ppb)	Impaired growth performance (↓ BWG) Impaired immune organ development (↓ thymus r.w., ↓ spleen r.w.)	-	-	No improvement was recorded for any of the other AFB_1_-affected parameters related to growth performance (FI), carcass performance, or liver health (liver r.w., serum total proteins, serum lipid profile), kidney health (kidney r.w.).	[123]
0.1	AFB_1_ (2.5 ppm)	-	-	Impaired growth performance (↓ BWG, ↓ FI, ↑ FCR) Altered intestinal microbiota composition (↓ Lactobacilli count; ↑ Coliform count) Impaired liver health (↑ serum ALT, AST, ALP, GGT, LDH activities; ↑ liver MDA levels, ↑ liver NO levels, ↓ liver SOD, CAT, and GPx activities) Impaired humoral immune response to vaccines (↓ anti- NDV, IBDV and AIV Ab titers)	The intestinal microbiota composition also showed improvement over the negative control **^(2)^**.	[59]
1	AFB_1_ (600 ppb)	-	Impaired growth performance (↓ BWG)	Impaired growth performance (↓ FI, ↑ FCR) Impaired carcass performance (↑ abdominal fat r.w.) Altered intestinal microbiota composition (↑ Coliform count) Impaired liver health (↑ serum ALT, AST activity; ↑ liver r.w.) Impaired immune function (↓ cellular response to PHA-P at 24 h; ↓ Ab response to SRBC antigens at day 28)	In this study, the protective efficacy of Spirulina was compared with that of milk thistle and a toxin binder. The overall duration of exposure to AFB_1_ was 42 days.	[124]
0.005–0.01–0.02	Arsenic (100 mg/kg)	Impaired liver health (↑ serum ALT, AST activity)	Impaired growth performance (↓ FBW)	-	The highest level (0.02%) was the most effective at exerting the protective effect. The WBC count was unaffected by arsenic and showed improvement over the negative control under the influence of dietary Spirulina.	[129]
0.1	Cadmium (100 ppm)	Impaired liver health (↑ serum ALT activity; ↓ liver GSH levels, ↑ liver TBARS levels) Impaired kidney health (↑ serum urea levels, ↑ serum creatinine levels, ↓ kidney GSH levels, ↑ kidney TBARS levels)	Impaired growth performance (↓ BWG)	-	The use of Spirulina was found to be associated with reduced liver and kidney levels of cadmium. In this study, the protective efficacy of Spirulina was compared with that of various herbal adaptogens (*Panax ginseng* and others).	[130]
2	Deltamethrin (300 mg/kg)	Impaired growth performance (↓ FBW, ↓ BWG, ↑ FCR) Impaired liver health (↑ serum ALT activity, ↑ serum AST activity; ↑ liver MDA levels, ↓ liver GSH levels, ↓ liver SOD activity; histopathological alterations) Impaired kidney health (↑ serum urea levels, ↑ serum creatinine levels; ↑ kidney MDA levels, ↓ kidney GSH levels, ↓ kidney SOD activity; histopathological alterations)	Impaired intestinal health (histopathological alterations)	-	Exposure to deltamethrin was found to induce increased FI, and dietary Spirulina counteracted this effect almost completely. The use of Spirulina was found to be associated with reduced deltamethrin residues in meat and liver.	[67]

The “↑ ”or “↓ ”arrows indicate that an increase or a decrease, respectively, was observed in the parameter with the experimental diet (Spirulina-containing) compared with the standard broiler diet (control). **^(1)^** Positive control: challenged chickens fed an unsupplemented diet; **^(2)^** negative control: non-challenged chickens fed an unsupplemented diet. **Abbreviations**: AFB_1_ = aflatoxin B_1_; FBW = final body weight; BWG = body weight gain; FI = feed intake; FCR = feed conversion ratio; r.w. = relative weight (% of the pre-slaughter weight); ALT = alanine aminotransferase; AST = aspartate aminotransferase; GGT = gamma-glutamyl transferase; ALP = alkaline phosphatase; LDH = lactate dehydrogenase; MDA = malondialdehyde; GSH = glutathione; SOD = superoxide dismutase; CAT = catalase; GPx = glutathione peroxidase; TBARS = thiobarbituric acid reactive substances; NO = nitric oxide; NDV = Newcastle disease virus; IBDV = infectious bursal disease virus; AIV = avian influenza virus; Ab = antibodies; WBC = white blood cell; PHA-P = phytoheamagglutinin-P; SRBC = sheep red blood cell.

Finally, it is worth mentioning the study by Ibrahim et al. (2021), who provided evidence for the ability of dietary Spirulina supplementation at an inclusion level of 2% to protect the chickens from the growth impairment and hepatorenal damage and dysfunction caused by the insecticide deltamethrin [67]. The exertion of this protection was indicated by the findings of improved growth indices, improved liver and kidney histopathological characters, and improved values of serum biochemical markers of hepatic and renal health in the supplemented intoxicated birds compared with the unsupplemented intoxicated ones. Considering that oxidative stress also plays a crucial role in the toxicity of deltamethrin [133], the finding that dietary Spirulina supplementation was also associated with improved liver and kidney tissue levels of oxidation and antioxidant markers (MDA, GSH, and SOD) [67] confirmed the mechanistic involvement of Spirulina’s antioxidant properties (Table 1) in the production of these protective effects [30,35].

### 4.2. Effects of Spirulina as “Alternative Protein Source” on Productive Performance and Health Status of Broiler Chickens

Compared with the use as feed supplement, the use of Spirulina as a partial or complete replacement for conventional protein-rich raw materials in the formulation of broiler diets has been less studied in the reviewed literature. Hence, the available information regarding the impact of this specific in-feed application on production indices of broiler chickens appears somewhat limited and not always consistent, probably also due to the heterogeneity of the experimental conditions under which it has been obtained.

In approaching the interpretation of the data from the published research, it is important to keep in mind that, in this context, the “growth-supporting” substances that are present in Spirulina (proteins in particular, but also others) have to compensate for the decreased presence (or absence) in the diet of the “growth-supporting” substances normally supplied by the “replaced” conventional protein source. Therefore, the “minimum positive outcome” to be expected in relation to the use of “Spirulina-based” diets is the maintenance of levels of productive performance (as well as of meat quality and animal health) that do not differ from (i.e., that are not inferior to) those achieved by the use of the conventional diets. Any improvements, where present, are welcome but not required, and where growth depression is recorded, this, in all likelihood, reflects the occurrence of some nutritional deficiencies and/or metabolic disturbances.

Soybean meal is undoubtedly the major conventional protein source for broiler feed, and most studies have focused on the use of Spirulina as a partial soybean replacer [1,39,53,73,134]. The search for alternatives to soybean meal for use in animal feeding is motivated by sustainability problems of modern broiler farming. Indeed, as the global population grows, there will be an increase in the consumption of chicken meat [29]. To keep up with the increasing worldwide demand for this high-quality animal food, global chicken meat production is projected to increase by 32% by 2030 and 59% by 2050 (from production in 2012), and this in turn will increase the demand for feedstuffs in general, but particularly for protein feeds [39]. To meet the increased protein needs of the growing broiler production sector, the soybean production would need to double by 2050 [29]. However, with the current crop yield gains (0.9–1.6% per year) [29] and the direct competition with human consumption [135], the availability of soybeans is anticipated to fall short [29], and this will also affect the market price of this protein source, which indeed is linearly rising [39]. In this view, identifying sustainable alternative protein sources that can be incorporated in broiler diets is one of the strategies proposed to fill this protein-gap, and thus alleviate the demand for soybean meal, and other soy-based feed materials, by commercial broiler (and livestock in general) producers [39].

Spirulina has received special attention as a replacement for soybean meal due to its high nutritional value (Table 1), which is comparable or even superior to that of the conventional vegetable protein feed [3,22]. Indeed, Spirulina, only except for crude fiber (which is lower), shows greater values of crude protein, as well as of lipids, gross energy, and mineral matter (including calcium and phosphorus) than soybean meal (which, for instance, contains “only” about 40% of crude protein) [22,23,28,29]. The rich protein content of Spirulina, which moreover can be produced with more efficient land use than the protein obtained from soy crops [22,28], also appears of high quality, given its satisfactory metabolizable coefficients (>50%) [28], and its well-balanced amino acid composition, which includes all the essential amino acids (Table 1). Most amino acids are present in Spirulina in greater quantity than in soybean meal; some others are detected in similar amount in the two protein sources [22,28,29]. Among the former there is methionine, and among the latter there is lysine, with both these amino acids playing a crucial role in supporting the quantity and quality of broiler meat production [14]. However, it is important to consider that for some amino acids (namely, glutamate, histidine, and proline), the content of Spirulina is lower than that of soybean [22,28,29]. In addition, it should be noticed that the calculation of crude protein provides an overestimation of the actual protein content of Spirulina, because of a relatively high amount (nearly 10%) of non-protein nitrogen (represented by nucleic acids, glucosamine and other amines, and nitrogenous substances in the microalga cell wall) that cannot be utilized by the chickens [23,28]. In light of the not complete overlap in the chemical composition of Spirulina and soybean meal, the suitability of Spirulina to meet the nutritional requirements of the fast-growing chickens (with satisfactory production outcomes) is still controversial, and the optimal levels for using this microalga as a substitute source of protein in a broiler diet have yet to be defined.

Regarding the latter point, the levels of dietary Spirulina inclusion that have been tested for this specific in-feed application in the reviewed literature range from a minimum of 1.5% [136] to a maximum of 21% [56]. However, another important aspect to be considered is that similar inclusion levels of Spirulina do not necessarily translate into similar replacement levels of soybean (Table 12), possibly also because of variations in the nutritional quality of the Spirulina products (and soy-based feed ingredients) used by the different authors. In light of this, we deemed it more appropriate and meaningful to arrange the present discussion by grouping the reviewed papers based on similar “soybean replacement levels” rather than on similar “Spirulina inclusion levels” (Table 12 and Figure 3).

***(a) Effects on Productive (Growth) Performance***

Looking at the studies that, by using Spirulina, realized soybean replacement levels ≤ 20%, there seems to exist general agreement regarding the fact that this feeding strategy would not affect the production indices of broiler chickens, leading, therefore, to a “positive outcome” [53,56,136,137]. More specifically, in the study by Ross and Dominy (1990), the replacement of 6.9% and 13.8% of total soybean by 1.5% and 3% of Spirulina, respectively, resulted in final BW and FCR values comparable to those recorded in the chickens fed the conventional soybean-based diet [136]. This study fed the test diets for the whole duration of a 41-day rearing cycle (i.e., from day of hatching to slaughter age) (Figure 3). Similar findings were reported more recently by Meyer et al. (2021), who used 2.5% Spirulina to replace about 10% of total dietary soybean over a 49-day rearing cycle (Figure 3) and found no performance effects in the broilers receiving this diet [137]. It is worth noting that both these prolonged feeding regimens, combined with the relatively low levels of Spirulina inclusion used and the limited amount of soybean replaced, may also be compatible with an application of Spirulina as a “feed supplement” (see Section 4.1.1) (Table 3). However, from this perspective, the dietary Spirulina “supplementation” realized by Ross and Dominy (1990) (1.5 and 3%) caused only numerical improvements in the growth performance parameters, which did not achieve statistical significance [136].

On the other hand, the dietary Spirulina “supplementation” realized by Meyer et al. (2021) (2.5%) proved able to produce some growth advantages (BW: +10%; BWG: 11.5%; and FI: +12.7%), even though these were detectable only by the end of the grower period (i.e., by day 28), and were lost during the subsequent weeks of the rearing cycle [137]. The fact that the two studies used different chicken genetic lines (Hubbard and Ross 708, respectively) might have contributed to this different response pattern to dietary Spirulina, with Hubbard chickens appearing overall more resistant than Ross 708 to the potential performance benefits offered by the microalga at these levels of dietary inclusion. The study by Evans et al. (2015) evaluated the effects of a diet in which 17.5% of soybean was replaced by 6% of Spirulina in Hubbard x Cobb 500 chicks [56]. In this case, no significant change in the growth performance indices of the birds was recorded. However, this study focused on the possibility of incorporating Spirulina as an alternative protein source in a starter diet, so the feeding period lasted only 18 days (starting from day 3 of age) (Figure 3). On the other hand, Zampiga et al. (2024), using Ross 308 chickens, evaluated the possibility of partially replacing soybean with Spirulina during later phases of a 41-day rearing cycle, particularly in both the grower and finisher diets (for a 27-day feeding period, till slaughter age), or only in the finisher diet (for a 12-day feeding period, till slaughter age) (Figure 3) [53]. The replacement of 20.2% soybean with 3% Spirulina in the finisher diet proved suitable for maintaining levels of growth performance equal to those recorded in the control chickens. The replacement of 16.7% soybean with 3% Spirulina in the grower–finisher diet (hence for a longer time) worked similarly well, except for a numerically higher FCR value.

As for the growth effects of using Spirulina to realize more field-relevant soybean replacement levels of 25–40%, the results reported by the same three groups of investigators appear conflicting. On the one hand, there are the studies by Ross and Dominy (1990) [136] and Evans et al. (2015) [56]. The first demonstrated that Hubbard broiler chickens could be fed a 6% Spirulina-containing diet in which soybean was reduced by 27.6% for the whole rearing cycle (41 days) without any changes in their growth performance [136]. The second, although adopting a feeding period of a shorter duration (18 days), limited to the starter phase, substantially confirmed this conclusion, documenting no changes in the growth-related parameters of Hubbard × Cobb 500 broiler chicks receiving a diet in which 34.8% of soybean was replaced by 11% of Spirulina [56]. Similar findings were also reported by Toyomizu et al. (2001), who fed Arbor Acres chickens on a diet containing 4% Spirulina (as a replacement for 27.1% soybean meal) for the last 16 days of a 37-day rearing cycle (Figure 3), and observed no variation in the growth performance, nor the carcass parameters [138]. On the other hand, Zampiga et al. (2023) reported that Ross 308 broilers fed a diet with 5% Spirulina as a replacement of about 27.3% of soybean for the first 22 days of their rearing cycle (starter + grower phase) (Figure 3) had significantly impaired growth performance (particularly BW: −8.7%; BWG: −10.7%; FCR: +7.6%) [139]. The negative impact of this diet, however, was found to be entirely reversed by feeding a conventional soybean-based diet for the subsequent 24 days, with achievement, by the slaughter age of 47 days, of growth performance indices similar to those of the chickens fed a conventional diet in all feeding phases. In another study by the same group, significant impairment of the growth performance of Ross 308 chicks (BW: −5.1%) was demonstrated to occur at the end of a 41-day rearing cycle, if 6% Spirulina replaced 40.4% of soybean in the finisher diet (i.e., for the last 12 days of the rearing cycle); the growth impairment was more pronounced (BW: −6.2%; BWG: −6.0%; FCR: +8.35%) if 6% Spirulina replaced 33.3% soybean in the grower–finisher diet (i.e., for the last 27 days of the rearing cycle) [53]. The different outcome of the study by Zampiga et al. (2023) [139] compared with that of the studies by Ross and Dominy (1990) [136], Evans et al. (2015) [56], and Toyomizu et al. (2001) [138] suggests that Ross 308, Hubbard (its hybrids included), and Arbor Acres chicks might have different tolerances towards the dietary replacement of soybean by Spirulina, seemingly lower in Ross 308 than in Hubbard and Arbor Acres chicks.

Similarly, conflicting outcomes were reported at 50–55% soybean replacement levels. In this regard, Evans et al. (2015) observed no growth impairment in Hubbard x Cobb 500 chicks after 18 days of feeding on a diet in which 52% of soybean was replaced by 16% of Spirulina [56]. The same lack of changes in the growth, as well as in the carcass performance, was reported by Toyomizu et al. (2001) for Arbor Acres chickens fed a diet containing 8% Spirulina as a replacement of 55.4% soybean meal for 16 days [138]. Slightly less positive results were reported in the study by Ross and Dominy, 1990 [136]. In this study, Hubbard chicks were fed a 55.2% soybean-replaced, 12% Spirulina-containing diet for a longer time (41 days), and the final BW of the birds, though not different from that of the control group, was found to be significantly lower than that of the chicks fed diets containing lower levels of Spirulina (1.5–6%), and hence lower levels of soy replacement (6.9–27.6%). Velten et al. (2018) obtained frankly negative results in terms of growth performance (BW: −51.1%; BWG: −52.4%; FCR: +40.0%; FI: −33.7%) by feeding Ross 308 chickens for the whole of a 34-day rearing period (Figure 3) on diets (starter and grower) in which 50% of soybean was replaced by about 10.75% of Spirulina [134]. Depressing effects on the growth performance of Ross 308 chickens (BW: −17.2%; BWG: −21.1%; FCR: +16.5%) were similarly reported by another group of investigators, who tested a 49.6% soybean-replaced, 15% Spirulina-containing diet for the last 21 days of a 35-day rearing period (Figure 3) [95,140].

Finally, when Spirulina replaces more than 55–60% of total dietary soybeans, the reviewed literature agrees on this feeding strategy’s detrimental influence on broiler chickens’ growth performance indices. In this respect, the less adverse outcomes were reported by Evans et al. (2015), who found that BW and FI were reduced by 2.9% and 5.8%, respectively, in Hubbard × Cobb 500 chicks fed for 18 days on a starter diet in which 69.8% of soybean was replaced by 21% of Spirulina [56]. Much more pronounced impairments of growth performance were reported for Ross 308 chickens by Zampiga et al. (2023) after 22 days of feeding on starter and grower diets containing 10% Spirulina as a replacement of 57.9% of soybean (BW: −24.2%; BWG: −25.3%; FCR: +15.8%; FI: −13.4%) [139]. The same authors recorded an even worse outcome after 22 days of feeding on starter and grower diets containing 15% Spirulina as a replacement of 82.7% soybean (BW: −50.4%; BWG: −53.1%; FCR: +50.2%; FI: −29.5%). Moreover, this negative impact was found to be mitigated, but not entirely reversed by re-feeding a conventional soybean-based diet during the subsequent 24 days, up to the slaughter age of 47 days (10% Spirulina/−57.9% soybean group: BW: −6.8%, BWG: −7.4%, FI: −9.9%; 15% Spirulina/−82.7% soybean group: BW: −18.9%, BWG: −19.7%, FI: −20.3%). Significant, though slightly less severe, impairment of some growth performance parameters was also observed in Ross 308 broiler chickens by Pestana et al. (2020) [141], in this case, when feeding the birds for the last 14 days of a 35-day rearing cycle (Figure 3) on a diet in which 60.9% of soybean was replaced by 15% of Spirulina (BWG: −11.5%, FCR: +10.1%, BW: numerically decreased).

Based on the information just discussed, and making generalizations with all due caution, it may be stated that (i) the dietary inclusion of Spirulina for modest soybean replacement levels (i.e., ≤20%) is tolerated by broiler chickens of different strains, during either specific feeding phases or the whole feeding cycle, leading to growth performance indices comparable to those of the birds fed a conventional soybean-based diet, and with some chance to even observe some improvements of these parameters [48,69,71,72,137] (see Section 4.1.1); (ii) the dietary inclusion of Spirulina for high soybean replacement levels (i.e., ≥56%) is not tolerated by broiler chickens of different strains, under different feeding regimens, leading to varying degrees of growth performance impairment (more or less pronounced depending on the specific chicken strain and feeding regimen); and (iii) the dietary inclusion of Spirulina for intermediate soybean replacement levels (i.e., >20% and ≤55%) can be successfully tolerated by broiler chickens or not, depending on the chicken strain and feeding regimen, with Ross 308 broilers appearing much less prone to tolerance than Arbor Acres and Hubbard chicks (and their hybrids). The explanations proposed for the negative influence exerted by Spirulina-rich diets on broiler chickens’ growth parameters are discussed in the following two paragraphs.

***(b) Effects on Intestinal Health***

Additional data from the studies conducted by some of the abovementioned research groups help provide a more in-depth understanding of the reasons why Ross 308 broiler chicks show reduced growth performance in response to diets in which about 50–60% of soybean is replaced by 15% of Spirulina [134,140,141,142]. More specifically, Pestana et al. (2020) found that the reduced growth performance of chickens fed this Spirulina-based diet was associated with increased digesta viscosity, and consistent with this, greater length of some intestinal compartments (duodenum and jejunum) was also observed in comparison with the control (conventionally fed) birds [141]. Interestingly, their study also evaluated the effect of supplementing the Spirulina-based diet with different exogenous carbohydrate-active enzymes (namely lysozyme on the one hand and a mixture of xylanases and β-glucanases on the other hand). The hypothesis was that these enzymes would have facilitated the disruption of the microalga cell wall (similar in structure and composition to that of Gram-negative bacteria), thereby enhancing the release of the microalgal nutrients, proteins included, for their subsequent digestion and utilization by the animal. However, despite the initial expectations, the broilers receiving the enzyme-supplemented Spirulina-based diet showed similar growth performance impairment to broilers fed the unsupplemented Spirulina diet. Moreover, in the chickens fed the Spirulina diet supplemented with lysozyme, the increase in the digesta viscosity resulted to be even more pronounced (two-fold increase compared with the control group).

Based on these findings, the authors hypothesized that the greater digesta viscosity could be a major determinant of the impaired growth performance of the birds, as the more viscous digesta would limit the access of the endogenous digestive enzymes to their target substrates, thereby reducing the overall digestibility of the feed and the rate of nutrient absorption. In addition, the authors argued that the increase in digesta viscosity observed in chickens fed the Spirulina-rich diet could have been due to the gelation of a probably large fraction of Spirulina-derived proteins that exceeded the digestive capacity of endogenous proteolytic enzymes. The use of lysozyme, by breaking the microalgal cell wall (much more efficiently than the mixture of xylanases and β-glucanases), would have further increased the extra-cellular release of the Spirulina proteins, leading to the occurrence of more gelation and then a more pronounced increase in digesta viscosity. In light of this scenario, the authors suggested that supplementing the Spirulina-based diet with a combination of lysozyme and exogenous specific peptidases could have been an effective strategy to improve both the release of the microalgal proteins and the efficiency of their enzymatic digestion, with consequent prevention of the detrimental protein gelation and its negative impact on the chickens’ growth performances [141].

In more recent studies by the same group, this hypothesis was verified but not confirmed [95,140]. More specifically, consistent with the previous findings, broilers fed a diet in which 15% of Spirulina replaced about 50% of total soybean were found to have increased viscosity of their intestinal content in association with impaired growth performance [140]. The supplementation of the Spirulina-based diet with a high dose (super-dosing) of a multi-enzyme mixture (consisting of the carbohydrase lysozyme and the peptidase pancreatin) showed actual efficacy at reducing the high digesta viscosity. However, it did not successfully mitigate the adverse effects of the diet on growth performance. On the other hand, in the same study, the impairment of broiler chickens’ growth performances was found to be in large part mitigated (with only the FCR value remaining higher than in control birds) by subjecting Spirulina to extrusion before incorporation in the broiler diet. This pre-treatment, however, did not prevent the gelation of the microalga proteins and subsequent increase in digesta viscosity.

Based on these results, the authors of these studies revised the scenario initially proposed, hypothesizing that the major determinant of the reduced growth performance could be the poor digestibility of some “valuable” proteins derived from Spirulina that would be somehow resistant to the proteolytic attack by endogenous, as well as exogenous (supplemented) proteolytic enzymes. The phycobiliprotein phycocyanin has been indicated as one of these “poorly digestible” Spirulina proteins [95,140]. This pigment protein is associated with the intracellular photosynthesizing thylakoid membrane of the microalga by stable bonds, and in this form, it could not be hydrolyzed by the digestive enzymes (peptidases). Due to this digestibility issue, the important amino acids contained within this protein would not be available for utilization by the chicken, and the resulting nutritional deficiency would account for the reduced performance. The extrusion pre-treatment would help break the protein–membrane chemical bonds and/or possibly induce a certain degree of denaturation of the protein structure, thereby exposing some otherwise inaccessible hydrolysis sites that are targets for digestive enzyme activity. In other terms, following extrusion, the bioaccessibility of complex Spirulina proteins like phycocyanin and their susceptibility to enzymatic attack would increase, leading to improved digestibility of these proteins and increased bioavailability of their amino acid components. In this context, the increased digesta viscosity, determined by the gelation of the excess amount of the (highly digestible) Spirulina-derived proteins, would “only” exert a worsening influence. In light of the so proposed scenario, the authors suggested that the use of pre-extruded Spirulina in combination with exogenous peptidases supplementation could be a suitable strategy to fully exploit the nutritional potential of the microalga when included in the broiler diet as a major protein source, alternative to soybean [95,140].

While waiting for the results of the ongoing research focused on this topic, other studies can be cited that seem to support the idea that the nutritional strategy based on the use of (non-extruded) Spirulina to replace large amounts of soybean meal may not be able to meet the amino acid requirements for the optimal growth and development of broiler chickens [33].

For instance, Velten et al. (2018) reported that Spirulina can be successfully used as a soybean replacer in the diet of Ross 308 broilers only under conditions of an extended level of dietary amino acid supplementation [134,142]. More specifically, these authors observed that replacing 50% soybean meal with (about 10%) Spirulina resulted in a significant depression of growth performance and feed efficiency data if the amino acid supplementation of the experimental diet was conducted at a basic level (i.e., adding amino acids equal to the control diet). In this respect, it is interesting to note that this impaired growth performance was found to occur in association with histomorphometric alterations of the intestinal wall that seem consistent with the increased digesta viscosity mentioned above (i.e., decreased thickness of the small intestinal *Tunica muscularis* layer, considered to be indicative of reduced gut motility). By adopting an extended level of amino acid supplementation of the diet, the growth response of the Ross 308 chickens fed the Spirulina-based ration for the whole duration of a 34-day rearing cycle was fully comparable to that recorded in birds fed the conventional control diet [134].

In the study by Zampiga et al. (2024), evidence was provided that the growth impairment detected in Ross 308 chickens fed a 33.3% soybean-replaced,6% Spirulina-containing diet for the last 27 days of their 41-day rearing cycle occurred in association with significantly decreased plasma levels of some metabolically critical amino acids (histidine and arginine) and amino acid-derived compounds (creatine, carnosine) [53]. Moreover, in the same study, the apparent ileal digestibility of some essential and non-essential amino acids was found to be slightly reduced (by 1.72–6.39%) even in the birds that showed a normal growth pattern in response to a 20% soybean-replaced, 3% Spirulina-containing diet, fed for the last 12 days of the rearing cycle (finisher diet).

Interestingly, Evans et al. (2015) documented the occurrence of decreased apparent ileal digestibility of various amino acids (aspartic and glutamic acid, proline, valine) in the growth-depressed Hubbard x Cobb 500 chickens fed on a 70% soybean replaced, 21% Spirulina-containing diet. By contrast, the 52% soybean-replaced, 16% Spirulina-containing diet, which was well tolerated by these chicks, resulted in values of amino acid digestibility similar to, or even greater than (depending on the specific amino acid) those measured for the control diet, and all other diets containing lower Spirulina levels (6 and 11%) [56].

***(c) Effects on General Health***

Although associated with reduced performance, digestibility issues, and some nutritional deficiencies, replacing 50–60% soybeans with 15% Spirulina in the diet seems not to seriously impact the chickens’ general health statuses. Various studies consistently reported that the birds remained healthy during the feeding trial, with no significant increase in the mortality rate being recorded [56,134,136,139]. One of the abovementioned groups of investigators also evaluated the animals’ health statuses by analyzing hematochemical parameters [95,141]. In this regard, Spinola et al. (2024) found no significant changes in the blood cell counts and hemoglobin levels, suggesting maintained hematological homeostasis [95]. Moreover, the serum biochemistry indicated that the Spirulina-based diet did not compromise the normal function of the liver and kidneys [95,141]. However, other findings were reported that would suggest that Spirulina, under this use condition, is no longer able to produce all of the beneficial influences that are typically observed when the microalga is used as a feed supplement, in all likelihood because of the abovementioned “compensatory function”. For instance, the systemic antioxidant defenses appeared not to increase, as indicated by the finding of unchanged serum total antioxidant capacity [141]. Also, no increase in serum total protein levels was recorded, unless the Spirulina-based diet was supplemented with the mixture of carbohydrate-active enzymes [141]. Moreover, and more importantly, no hypolipidemic effect could be observed. Instead, an increase was consistently recorded in the serum lipid parameters (total lipids, total cholesterol, LDL-cholesterol, and triglycerides) [95,141]. Interestingly, this lipemic boost, attributed by the authors to enhanced fat absorption in the intestinal tract [95], was found to be almost wholly counteracted (leading to values not different from those measured in the control chickens) when the Spirulina-based diet was supplemented with a combination of lysozyme and pancreatin [95,140].

Spinola et al. (2024) also demonstrated a diet effect on the liver’s chemical composition [95]. However, in this case, most of the changes were overall beneficial and included reduced total lipid content, improved fatty acid profile (increased content of *n*-3 polyunsaturated fatty acids, leading to favorably decreased *n*-6/*n*-3 ratio), and increased content of antioxidant pigments (carotenoids and chlorophylls), which plausibly helped protect the hepatic tissue from oxidative stress. Opposite to these positive effects, a significant decrease in the liver content of α-tocopherol and a significant increase in the liver content of cholesterol were found to consistently occur in association with the Spirulina-based diet (regardless of extrusion or exogenous enzyme supplementation).

Of course, data from only two studies conducted by the same group of investigators are too scarce for any generalizations. Moreover, it should be pointed out that these data relate to an experimental feeding period of relatively short duration (21 days) that begins after two weeks of feeding on a conventional soybean-based diet (Figure 3). Another aspect to consider is that the experimental conditions adopted in these studies do not allow a distinction between effects attributable to the dietary inclusion of Spirulina and effects determined by the removal of dietary soybeans. Therefore, as also remarked by other authors [53,61,62], further research is needed to explore the health-related effects of using relatively large amounts of Spirulina to replace relatively large quantities of dietary soybean over extended feeding periods so as to assess the safety of this nutritional strategy for broiler chickens.

Staying on the theme of the health-related effects of Spirulina used as a partial soybean meal replacer, another perspective can be discussed: low-protein diets. Besides incorporating alternative protein sources into diets, the formulation of broiler diets with a reduced content of total crude protein (CP) has also been proposed as a nutritional strategy to alleviate the global demand for soybean meal and other soy-based feedstuffs [29,39]. However, lowering dietary CP has been shown to compromise broiler chickens’ health and performances. More specifically, broilers fed a diet in which CP was allowed to decrease to 17% (compared with the 20% CP of the standard control diet) by reducing the overall amount of dietary soybean meal from 31% to 22% were found to have increased liver bacterial translocation (likely reflecting increased intestinal permeability and leakage) and increased markers of systemic inflammation (namely, increased proportions of basophils within the total circulating leukocytes and up-regulated expression of pro-inflammatory cytokines in blood leukocytes) [39]. These health-related alterations occurred in association with (and probably partly accounted for) decreased growth performance and feed efficiency and reduced carcass performance (decreased carcass yields and increased fat deposition) [29].

Replacing 50% of soybean meal in the low-protein diet with 10% Spirulina proved to reduce liver bacterial translocation to levels that were even lower than those measured in chickens fed the standard CP control diet, suggesting a probable enhancement of intestinal barrier integrity [39]. Moreover, and in partial agreement with this finding, the partial replacement of soybean meal with Spirulina was found to almost completely mitigate the signs of systemic inflammation, probably also thanks to the direct anti-inflammatory and the antibacterial activities of substances contained in Spirulina (e.g., C-phycocyanin). Nevertheless, the negative impact on the productive performance indices was not mitigated [29].

Based on these findings, using Spirulina as a partial soybean replacer in formulating low-protein diets for broilers may offer some health-related advantages but seems unlikely to represent a solution to all of the issues related to this soybean-sparing nutritional approach.

### 4.3. Effects of Spirulina as Either a “Functional Feed Supplement” or an “Alternative Protein Source“ on the Quality of Broiler Chickens’ Meat

As stated above, Spirulina is a rich source of high-quality proteins with a well-balanced amino acid profile, as well as of vitamins, pigments, minerals, antioxidants, and polyunsaturated fatty acids (PUFAs), particularly γ-linolenic acid (GLA) (Table 1). Hence, it is no surprise that its use as a “feed supplement” has been reported to also positively influence diverse aspects of broiler meat quality [51,73].

Changes in broiler meat quality have also been documented in association with using Spirulina as a “replacement of conventional protein sources”, such as soybean meal. However, in this case, only some changes resemble those in the meat from Spirulina-supplemented broilers. Other changes occur in the opposite direction of the latter, and some others seem only (or mainly) detectable in the meat from broilers fed “soybean-replaced” diets, with outcomes that may not always meet consumer expectations [51,73].

The partially different modifications occurring in the quality of the “soybean-replaced” meat, as compared to the “supplemented” meat, may somehow be related to the different (and plausibly higher) amount of Spirulina-derived substances that are transferred to the muscle tissue after digestion of the more abundant quantity of ingested Spirulina [51]. These substances, at higher concentrations, may produce effects that are different from those produced at lower concentrations, or effects that at lower concentrations are not produced. In addition, a causal contribution may derive from specific nutritional deficiencies. The latter, as already discussed in relation to the adverse effects of high Spirulina intake levels on broiler growth performance (see Section 4.2), may result from the inability of Spirulina to adequately compensate for the partial or complete removal of soybean-based products due (at least in part) to the incomplete bioaccessibility and/or bioavailability of some of its valuable nutrients (particularly thylakoid membrane-associated proteins, and their constitutive amino acids) [51,53].

It is also worth noting that some of the meat quality changes associated with Spirulina-based diets seem to occur less consistently (i.e., more variably, and hence less predictably) than meat quality changes associated with Spirulina-supplemented diets, and this happens even when findings from the same group of investigators are examined. As some authors have partly suggested, this may signify that, under the circumstance of a relatively high Spirulina intake (as usually required for partial or complete replacement of conventional soybean protein), other influencing factors (possibly including the abovementioned digestibility-related issues) can variably intervene, and thus variably modulate the response of certain meat quality traits to dietary Spirulina [51].

At any rate, it must be acknowledged that the amount of research specifically focusing on the impact of dietary Spirulina on broiler meat quality is still limited and has been conducted only by a few groups of investigators. Therefore, any generalizations from this paucity of data should be approached with caution.

***(a) Effects on meat nutritional composition and oxidative stability***

One of the meat quality parameters that dietary Spirulina supplementation has been shown to influence positively is the fatty acid composition of the lipid fraction, with consequent enhancement of the nutritional value of this food product (Table 13). Notably, in the study by Bonos et al. (2016), a significant enrichment of certain beneficial *n*-3 PUFAs (mainly eicosapentaenoic acid—EPA and docosahexaenoic acid—DHA) was reported to occur in the broiler thigh muscle following administration of 0.5 and 1% Spirulina-supplemented diets [49]. Two more recent studies obtained similar results using slightly lower or higher levels, respectively, of dietary Spirulina supplementation [46,47]. More specifically, El-Bahr et al. (2020) measured greater contents of total PUFAs, total *n*-3 PUFAs (including EPA and DHA), and arachidonic acid (*n*-6 PUFA) in the breast muscle from broiler chickens fed 0.1% Spirulina-supplemented diet for 32 days, than in the meat from the control (unsupplemented) birds [47]. Sharmin et al. (2020) found that including 1.5% (but not 1%) Spirulina in a broiler diet for 5 weeks was functional to obtain increased levels of *n*-3 PUFAs (including DHA and linolenic acid) in breast and thigh meat [46].

Increased muscle lipid oxidation may be a drawback of an increased content of the oxidation susceptible *n*-3 PUFAs (causing the development of off-flavors and reducing meat color stability) [1,46,49,51]. Nevertheless, none of the abovementioned studies documented increased meat lipid oxidation in association with dietary Spirulina supplementation. Instead, in one of the studies, lipid oxidation in the meat from Spirulina-supplemented broilers was found to remain unchanged as compared with control meat (notably, no change was detected in the levels of thiobarbituric acid reactive substances—TBARS) [49]. In the other two studies, dietary Spirulina supplementation was associated with reduced meat lipid oxidation, as indicated by the measurement of lower levels of MDA [47] and lower levels of TBARS [46] compared with the control unsupplemented diet. Moreover, El-Bahr et al. (2020) also reported lower protein carbonyl values (indicative of reduced oxidative protein damage), as well as increased muscle SOD activity, suggesting that reduced levels of oxidation could be due to the concomitantly increased antioxidant capacity of the meat [47]. In all likelihood, a direct contribution to the overall improved oxidative stability of the broiler meat could also be provided by the antioxidant components of Spirulina (e.g., phycocyanin, carotenoids) (Table 1), transferred to the muscle tissue after absorption from the intestine [47,51,73].

Always in terms of fatty acid composition and oxidative stability of broiler meat, partially different results were reported by authors who incorporated Spirulina in the chickens’ diets as a soybean replacer (Table 13). In particular, in the study by Pestana et al. (2020), a diet in which about 60% of soybean was replaced by 15% of Spirulina also proved able to modify the fatty acid composition of the meat in comparison with the control group (either in presence or absence of exogenous carbohydrate-active enzymes) [141]. However, in this case, greater values of saturated fatty acids (SFAs), lower levels of *n*-3 PUFAs, and bidirectional variations in the levels of some specific *n*-6 PUFAs (including a two-fold increase in GLA, and a 12% decrease in linoleic acid) were recorded.

In a similar, more recent study by the same group, a higher content of SFAs was confirmed to occur in the “soybean-replaced” meat (predominantly of palmitic acid, which is known to have potentially harmful health implications for humans by increasing the cardiovascular disease risk) [140]. Moreover, lower levels of total PUFAs were measured in both breast and thigh meat. However, unlike previous findings, among PUFAs, only *n*-6 PUFAs were found to be decreased (with GLA and linoleic acid being both decreased by about 30%), whereas an increase occurred in the *n*-3 PUFAs (especially EPA and DHA). As a consequence of the changes detected in the PUFA profile, Costa et al. (2024) found the *n*-6/*n*-3 PUFA ratio of the “soybean-replaced” meat to be favorably decreased compared with that of the meat obtained from chickens fed the control diet (and interestingly, this decrease resulted to be more marked when extruded Spirulina was used as the soybean replacer). However, despite this decrease, the *n*-6/*n*-3 PUFA ratio values calculated for the “soybean-replaced” meat (ranging from 17.5 to 23.2) continued to be much higher than the ideal value recommended for healthy food (less than 4) [73,143]. In addition, as a consequence of the whole of the changes occurred in the fatty acid profile, the meat obtained from broilers fed the Spirulina-based diet also showed (i) significantly decreased PUFA/SFA ratio values (that, although remaining higher than the recommended minimum of 0.49, were more distant from the optimal values of 1.4–2.4 than the values calculated for the control meat) and (ii) significantly increased values of the health-related lipid indices “atherogenic index” and “thrombogenic index” (again with slight, but significant deviation from values recommended for a healthy diet) [140]. So, as concluded by the authors, although the high level of Spirulina intake was associated with a potentially beneficial increase in *n*-3 PUFAs, the total amount of these fatty acids remained too small and was not enough to counterbalance the increase in SFAs and have a major favorable impact on the athero/thrombogenic potential of the broiler meat.

**Table 13:** Overview of the studies evaluating the effects of dietary Spirulina (used as either a feed supplement or a soybean replacer) on the nutritional composition and oxidative stability of broiler chickens’ meat.

SP Inclusion Level (%)	Soybean Replacement Level (%)	FA Profile	Oxidative Stability		Other Components	Notes	Reference
			Oxidation Markers	Antioxidant Defenses			
0.1	-	Breast: ↑ total PUFAs, total *n*-3 PUFAs, EPA, DHA, AA	↓ MDA ↓ PC	↑ SOD activity	↑ Essential amino acids (lysine, methionine, tryptophan, histidine) ↑ Non-essential amino acids (aspartic acid)	-	[47]
0.5–1	-	↑ EPA, DHA	↔ TBARS	-	-	-	[49]
1–1.5	-	Breast and thigh: ↑ total *n*-3 PUFAs, DHA, linoleic acid	Breast and thigh: ↓ TBARS	-	Breast: ↑crude fat	Only the higher supplementation level (1.5%) was effective at producing the change.	[46]
2	-	-	-	-	↑ Protein ↓ Total lipids, cholesterol, triglycerides	In this study, this supplementation level of Spirulina: - Ensured mitigation of the negative influence exerted by exposure to deltamethrin (300 mg/kg) on these parameters; - Resulted in lower residues of deltamethrin in the meat.	[67]
15	60.9	Breast and thigh: ↑ SFAs ↓ *n*-3 PUFAs ↑GLA ↓ Linoleic acid ↓ PUFA/SFA ratio ↑ *n*-6/*n*-3 PUFA ratio	↔ TBARS	↑ Total carotenoids, total chlorophylls, and chlorophylls plus carotenoids ↓ α- and γ- tocopherol	↔ Total lipids, cholesterol	In this study, the effects of dietary Spirulina were also evaluated in combination with carbohydrate-active enzymes (lysozyme or a mixture of xylanase and β-glucanase).	[141]
15	49.6	Breast and thigh: ↑ SFAs (palmitic acid) ↓ Total PUFAs ↑ *n*-3 PUFA (EPA, DHA) ↓ *n*-6 PUFA (GLA, linoleic acid) ↓ *n*-6/*n*-3 PUFA ratio ↓ PUFA/SFA ratio ↑ AI, TI	↔ TBARS	↑ Total carotenoids, total chlorophylls and carotenoids ↓ α-tocopherol, γ-tocopherol + β-tocopherol	↓ Total lipids ↔ cholesterol ↓ Zn	In this study, the effects of dietary Spirulina were also evaluated after extrusion processing of Spirulina or in combination with a mixture of lysozyme and pancreatin.	[140]
11.8 (s)–9.7 (g)	50	-	↔ TBARS	-	Breast: ↔ protein, intramuscular fat, moisture	This study was performed in Ross 308 broiler chickens; The feeding trial lasted 35 days and was arranged in two feeding phases (starter and grower).	[144]
22.1 (s)	75	↑ MUFA ↓ Total PUFAs, ↓ *n*-6 PUFA, ↓ Linoleic acid, ↓ *n*-3 PUFA	↑ TBARS	-	-	This study was performed in Ross 308 broiler chickens; The feeding trial lasted 35 days and was arranged in two feeding phases (starter and grower); the soybean replacement level was 75% and 50% in the starter and grower diets, respectively. The changes in TBARS were recorded in breast meat packaged in a highly oxygenated modified atmosphere.	[11]
12.5 (g)	50						
23 (s)–20 (g)	100	-	-	-	↓ Bioactive compounds (anserine, creatine, carnosine)	This study was performed in Ross 308 broiler chickens; The feeding trial lasted 35 days and was arranged in two feeding phases (starter and grower).	[145]

The “↑ ”, “↓ ”, or “↔” arrows indicate that the parameter increased, decreased, or did not change, respectively, with the experimental diet (containing Spirulina) compared with the standard broiler diet (control). **Abbreviations**: PUFAs: polyunsaturated fatty acids; SFAs = saturated fatty acids; MUFAs = monounsaturated fatty acids; EPA = eicosapentaenoic acid; DHA = docosahexaenoic acid; AA = arachidonic acid; GLA = γ-linolenic acid; MDA = malondialdehyde; PC = protein carbonyl; SOD = superoxide dismutase; TBARs = thiobarbituric acid-reactive substances; AI = atherogenic index; TI = thrombogenic index.

Regarding the antioxidant capacity and oxidative stability of the “soybean-replaced” meat, the studies by Pestana et al. (2020) [141] and Costa et al. (2024) [140] consistently reported bidirectional variations in the meat contents of some antioxidant compounds. More specifically, greater values of total carotenoids and reduced levels of α-tocopherol were measured. The authors explained the latter finding as a consequence of the fact that the Spirulina product used for their study (probably due to some particular growth conditions used for the microalga production) had lower content of this vitamin than reported in the consulted literature (50–190 mg/kg dry weight) [25,140]. However, other authors have reported that concentrations of tocopherol in the raw microalga material are generally low (ranging from 0.011 to 0.014 mg tocopherol/g dried Spirulina) [146], and this warrants attention in the formulation of a diet for broilers that contains high proportions of Spirulina. Despite the diminished tocopherol content, the “soybean-replaced” meat did not show changes in its oxidative stability. Indeed, the lipid oxidation level of breast and thigh meat (as measured by TBARS) was found to not be modified by the Spirulina-based diet compared with the control diet [140,141]. In all likelihood, the increased total meat content of Spirulina-derived carotenoids played a compensating and more critical role in ensuring the maintenance of the oxidative stability of the meat.

Similar results in terms of oxidative stability (i.e., no change in TBARS) were reported by Altmann et al. (2018) for the meat obtained from broilers fed a diet in which Spirulina powder replaced 50% of soybean meal (at a final inclusion level of 11.8% and 9.7% in the starter and grower diets, respectively) (Table 13) [144]. However, it is of note that in a subsequent packaging trial conducted by the same authors, the meat obtained from chickens fed Spirulina (this time used as a 75% and 50% soybean replacement in the starter and grower diets, respectively, at final inclusion levels of 22.1% and 12.5%) showed higher lipid oxidation levels (as measured by TBARS) than the control meat, after storage under conditions of highly oxygenated (80%) modified atmosphere packaging (HiOxMAP) [11]. This would suggest that meat from broilers fed Spirulina as a soybean replacer might have an inherently higher susceptibility to oxygen, which could be exacerbated by certain packaging conditions used in the meat industry [11,73].

Besides changes in fatty acid and antioxidant content and profile, other modifications in the nutritional composition of broiler meat have been reported to occur in response to the dietary administration of Spirulina, both in terms of macro- and micro-nutrients. The data available in this regard are more limited but again suggest a rather favorable influence exerted on the meat nutritional quality by the relatively low Spirulina inclusion levels used for “feed supplementation” and a less desirable influence exerted instead by the higher Spirulina inclusion levels used for “soybean replacement” (Table 13).

In the study by El-Bahr et al. (2020), broilers receiving a diet supplemented with 0.1% Spirulina had breast muscle with increased levels of the essential amino acids lysine, methionine, tryptophan, and histidine, as well as of the non-essential amino acid aspartic acid, compared with birds that received no supplementation [47]. With the higher levels of dietary Spirulina supplementation (1.5%) used by Sharmin et al. (2020), an increase in the crude fat content of broiler breast meat was recorded [46]. However, in response to the 2% Spirulina-supplemented diet used by Ibrahim et al. (2021), the broiler meat showed a significant increase in protein content and a significant decrease in fat content, along with decreased levels of harmful cholesterol and triglycerides (important contributors to cardiovascular diseases), compared with the meat of the control unsupplemented group [67]. Interestingly, in this same study, it was found that Spirulina, at this level of supplementation, was able to mitigate the negative impact that deltamethrin toxicity had on these specific biochemical components of broiler meat (and that consisted in a decrease in protein, and increase in fat, cholesterol, and triglycerides).

Based on the study performed by Costa et al. (2024), a decrease in total lipid content would also be appreciable in the meat (breast muscle) obtained from broilers fed diets incorporating 15% Spirulina as a replacement for about 50% soybean [140]. However, in a previous study by the same research group [141], no change occurred in the total lipids of the meat in response to a similar Spirulina-based diet. As for the cholesterol levels of this meat, the two studies were consistent in reporting no significant influence exerted by the 15% Spirulina-containing diet on this parameter [140,141].

Costa et al. (2024) also found that breast and thigh meat from broilers fed the 50% soybean-replaced and 15% Spirulina-containing diet had reduced zinc content (by about 30%) compared with the control meat [140]. This observation raises further concern about the nutritional completeness of the “soybean-replaced” broiler meat. Moreover, considering that Spirulina is consistently reported to be a rich source of this essential mineral element [5,12,19,62,76,95], this finding by Costa et al. (2024) [140] seems to confirm that the relationship between the innate nutritional composition of this microalga (as of any other ingested feed ingredients) and body composition is not always straightforward [95] (see Section 4.1.2).

Worthy of mention is also the study by Gkarane et al. (2020), who reported that breast meat from chickens fed diets in which 100% soybean meal was replaced by Spirulina (at the final level of 23% and 20% in the starter and grower diets, respectively) had reduced levels of the endogenous bioactive compounds anserine, creatine, and carnosine (3.3 vs. 4.1 mg/g, 0.15 vs. 0.72 mg/g, and 1.49 vs. 2.49 mg/g, respectively) compared with meat from birds fed the control diet (Table 13) [145]. This also poses a nutritional concern, as low creatine levels may negatively influence neurodegenerative disorders and athletic performance of the human consumers [147,148]. Moreover, low contents of carnosine (and other histidine-containing peptides) could be associated with reduced scavenging capacity and reduced protective potential against cardiovascular diseases [149]. According to the authors, the reduced carnosine content measured in the broiler meat was at least in part attributable to the smaller amount of histidine that they detected in the soybean-replaced, Spirulina-containing diet (as compared with the conventional control diet) [147], which would be consistent with the lower content of histidine that has been reported for the protein derived from Spirulina as compared with the protein derived from soybean meal [22,28,29]. As already mentioned in Section 4.2 (see above), a recent study by Zampiga et al. (2024) proved that lower-than-normal circulating levels of the amino acid histidine and dipeptide carnosine could be documented in broiler chickens fed Spirulina-based soybean-replaced diets, even though this metabolic alteration was observed in association with diets in which only a modest amount of soybean meal was replaced by relatively low levels of Spirulina (3–6%) (Table 12; Figure 3), and the responsibility for its occurrence was attributed to a concomitant decrease in the intestinal digestibility of the amino acid [53].

***(b) Effects on Meat Physicochemical Properties and Sensory Attributes***

Among the physicochemical properties of broiler meat, color shows clear susceptibility to the influence by dietary Spirulina (Table 14). This seems particularly true when Spirulina is included in the broiler diet as an “alternative protein source” [1].

Indeed, in the studies by Pestana et al. (2020) and Costa et al. (2024), greater values of yellowness (b*) (up to three times) were observed in the breast muscle and greater values of both redness (a*) and yellowness (b*) were observed in the thigh muscle of chickens fed diets containing 15% Spirulina as a replacer of 50–60% of soybean [140,141]. This finding was suggested to reflect the increased content of total carotenoids by which this “soybean-replaced” meat was also characterized (see above and Table 13), given that carotenoids are not only antioxidants but also pigments [76].

Similarly, Altmann et al. (2018, 2020) reported that the meat obtained from chickens fed Spirulina-based diets (particularly, diets containing Spirulina either at 11.8% and 9.7% to replace 50% soybean meal in both starter and grower diets or at 22.1% and 12.5% to replace 75% and 50% soy protein in starter and grower diets, respectively) had higher values of redness (a*) and higher values of yellowness (b*), resulting in a dark reddish-yellowish color [11,144]. As specified by the investigators, the distinctive meat color not only resulted from the instrumental analytical measurement but was also perceptible to the naked eye and still appreciable by the end of the storage period under conditions of HiOxMA packaging.

The color enhancement effect associated with the use of Spirulina as an alternative protein source was also documented in an earlier study by Venkataraman et al. (1994), who detected more intense red and yellow hues in the meat obtained from chickens fed diets containing 140 g/kg (14%) and 170 g/kg (17%) of Spirulina as a replacement, in this case, of either fishmeal or groundnut cake, respectively [40].

Interestingly, the enhancing effect of Spirulina on chicken meat color was also clearly observed at lower levels of dietary protein replacement by Spirulina. More specifically, Toyomizu et al. (2001) found that both yellow and red indices of chicken meat were increased in response to dietary inclusion of Spirulina at 4% and 8% [138]. The yellowness increased in a sub-linear fashion with the increase in dietary Spirulina and showed a correlation with the meat content of the Spirulina-derived carotenoid zeaxanthin (yellow pigment). By contrast, the rise in meat redness was already maximal in the chicks fed the 4% Spirulina diet [138], and according to some authors, it would be related to an increment of myoglobin levels induced by the high iron and mineral content of the microalga [150]. In accordance with Toyomizu et al. (2001), Zampiga et al. (2024) reported enhanced redness and yellowness of breast meat in broilers fed 3% and 6% Spirulina-containing diets (either during the finisher phase or during both the grower and finisher phases) (also see Table 12, Figure 3), with the intensity of the yellow pigmentation increasing as the dosage and duration of Spirulina administration increased [53,138].

However, it is apparent from the literature that, with Spirulina levels in feed equal to or lower than 5% (including levels more properly indicative of dietary supplementation), the impact on broiler meat color becomes less pronounced and less consistent (Table 14). Indeed, Raach-Moujahed et al. (2011), who tested Spirulina as a growth-promoting supplement at levels of 1%, 2.5%, and 5.0% (with partial proportional replacement of soybean meal), reported no change in meat color, even though higher values of yellowness (b*) were recorded with 2.5 and 5.0% dietary Spirulina [72]. Park et al. (2018) also tested dietary Spirulina at 1% and lower levels (0.25, 0.5, 0.75%) and observed no changes in breast meat color [63].

According to some authors, the whole of these findings suggests that the color enhancement effect of Spirulina on broiler meat would increase gradually with increasing dietary inclusion levels of Spirulina; as long as these levels are relatively low, then it would reach a saturation point, beyond which further increases in the levels of Spirulina intake would not further enhance the broiler meat color [51]. The same authors presented this saturation effect as a “critical observation”. However, a more important point is whether the Spirulina-enhanced meat color (although indicative of an enriched meat content of antioxidant carotenoids) can enhance consumer appeal, given that color is one of the most important food quality parameters consumers perceive [33,140]. In this regard, it has been argued that a darker meat color could be advantageous in consumer acceptability [38,141,144]. However, the intense orange color particularly and consistently observed in the meat obtained from chicken-fed diets in which relatively high levels of Spirulina are used to partially or wholly replace conventional protein sources may not be universally accepted by meat consumers. Indeed, at least concerning chicken skin, in countries like Mexico and, to a varying degree, the United States, consumer preferences currently are for a yellow-orange color, which is culturally associated with a good health status of the bird; by contrast, in Europe, a pale skin color is generally preferred [140]. At any rate, it is important to also consider the observations reported by Pestana et al. (2020), who noticed that when the “soybean-replaced” chicken breast meat was cooked, the trained sensory panel did not distinguish a color difference between samples from Spirulina-fed chickens and control chickens [141].

Besides color, dietary Spirulina seems able to influence other physicochemical properties of broiler meat, as well as some sensory attributes, though data available in this regard are again limited and somewhat variable (Table 14). With particular regard to the use of Spirulina as a “feed supplement”, El-Bahr et al. (2020) found that a supplementation level of Spirulina as low as 0.1% was able to reduce meat cooking loss, with no effect on thawing loss [47]. Park et al. (2018), using slightly higher Spirulina supplementation levels (0.25, 0.5, 0.75, and 1.0%), detected no changes in cooking loss; however, drip loss after 7-day refrigerated storage was found to decrease linearly with increasing Spirulina supplementation [63]. Taken together, these findings may mark an improved water-holding capacity of the broiler meat [151], and this, as partly suggested by the authors [63,73], seems consistent with the improved oxidative stability documented for this meat (see above) [47]. Indeed, reduced lipid and protein oxidation would translate into delayed loss of cell membrane and protein integrity, with consequent enhanced capability of muscle fibers to retain water [63,151].

Despite the lack of any appreciable changes in its oxidative stability (see above and Table 13), the meat obtained from broilers fed a diet in which 50% of soybean meal was replaced with Spirulina (in both the starter and grower diets, with final Spirulina inclusion levels of 11.8% and 9.7%, respectively) was also found to show greater water-holding capacity during storage and cooking [146]. Similarly, Zampiga et al. (2024) reported reduced meat drip loss (with unchanged cooking loss) in response to a Spirulina-based diet (6% Spirulina replacing 33.3% soybean) fed during both the grower and finisher phases and attributed this outcome to the antioxidant activity exerted by the carotenoid pigments [53].

As for the meat pH, Park et al. (2018), at the tested levels of dietary Spirulina supplementation (0.25, 0.5, 0.75, and 1.0%), detected no changes in the value of this parameter in breast meat [63]. Raach-Moujahed et al. (2011) found no changes in pH values of chicken meat at dietary Spirulina inclusion rates ranging from 1.0 to 5.0% (and corresponding soybean replacement levels ranging from 3.1 to 18.8%) [72]. The latter authors also reported no changes in sensory attributes such as tenderness, juiciness, odor, and flavor. No apparent influence on meat pH was also reported by Zampiga et al. (2024) with diets in which about 18.5% and 36.9% of soybean meal was replaced by 3% and 6% of Spirulina, respectively [53].

With higher Spirulina inclusion levels to replace higher amounts of soy-based protein in the broiler diet, the information regarding the impact on broiler meat pH and sensory attributes becomes less consistent (Table 14). Indeed, in the studies by Pestana et al. (2020) and Costa et al. (2024), the pH value of the breast meat obtained from chickens fed a 50–60% soybean-replaced, 15% Spirulina-containing diet was found to be either unchanged or decreased, respectively [140,141]. Moreover, Pestana et al. (2020) reported that sensory attributes such as tenderness, flavor, off-flavors, and overall acceptability did not vary for this “soybean-replaced” meat compared with the control meat [141]. Only juiciness was found to be reduced [141]. By contrast, appreciable (and overall, potentially positive) changes in the abovementioned aspects of meat quality were reported by other authors who used Spirulina to replace 50–100% of soybean meal in a broiler diet. More specifically, in the study published by Altmann et al. in 2018, it was found that replacing 50% of soybean meal with Spirulina (with final Spirulina inclusion levels of 11.8% and 9.7% in the starter and grower diets, respectively) resulted in more tender breast filets, with higher pH, and reduced unpleasant (metallic) off-flavor [144]. In a subsequent study, the same authors reported that feeding starter and grower diets in which 22.1% or 12.5% of Spirulina replaced 75% or 50% soybean in the diet, respectively, resulted in a slightly increased umami and, therefore, chicken flavor, which made the meat taste all-the-more like chicken [11]. In two further studies by the same group, the latter finding was explored more in-depth, and evidence was collected that replacing 50–100% of soybean with Spirulina induced significant changes in the profile of several compounds that play key roles in the development of the typical aroma of chicken meat [145,152]. A particularly interesting finding was the presence in the “soybean-replaced” broiler meat of increased levels of the flavor-related compound inosine-5′-monophosphate, which was suggested to reflect the high purine nucleotide content of the Spirulina-containing diet [145].

***(c) Effects on Meat Microbiological Quality and Safety***

There are two more and partially interrelated aspects of broiler meat quality on which dietary inclusion of Spirulina has been reported to exert an influence.

Firstly, the overall microbiological quality of the meat. In this regard, El-Bahr et al. (2020) reported that, after 1 day and after 5 days of chilling, the breast meat from broilers receiving a Spirulina-supplemented diet had a lower aerobic plate count compared with the meat from the unsupplemented control birds [47]. Although the Spirulina intake level used in that study was relatively low (0.1%), the authors explained this positive effect in terms of shelf-life in light of the high Spirulina content of substances endowed with antimicrobial activity and likely transferred to the meat after intestinal absorption.

Secondly, the safety of the meat for the human consumer. Food safety is a prerequisite for food quality, and safety perception, like quality perception, is a central issue in today’s food economics because it can influence consumers’ choice and demand for food [153].

In this view, evidence suggests that dietary Spirulina supplementation may offer potential advantages in terms of improved broiler meat’s chemical safety. More specifically, Ibrahim et al. (2021) reported that the inclusion of 2% Spirulina in broiler diet is effective in lowering the chemical contamination by residues of deltamethrin in chicken meat, as well as in the skin and liver [67]. Deltamethrin is a synthetic pyrethroid commonly used in veterinary medicine as an ectoparasiticide and in agriculture as a pesticide, with known adverse effects on human health (skin, respiratory, gastrointestinal, and neurological symptoms). The mechanisms underlying dietary Spirulina’s “decontaminating” effect have not been defined. A possible explanation might be the occurrence of a direct chemical interaction, within the intestinal environment or systemic circulation, between the ingested deltamethrin and dietary Spirulina components that would act like chemical binders or chelators, leading to reduced bioavailability and/or facilitated elimination of the contaminating agent. A similar mechanism has been proposed to account for the documented ability of Spirulina (through its content of flavonoids and phycocyanin) to reduce the accumulation of heavy metals in broiler tissues as part of its more general protective efficacy against heavy-metal-induced toxicity [130,131] (see Section 4.1.3). Alternatively (or additionally), the residue reduction effect might be the indirect consequence (i) of the protection exerted by Spirulina on chickens’ livers and kidneys against deltamethrin-induced toxicity (as a preserved hepato-renal function would allow appropriate deltamethrin elimination from the body) [67,133], and/or (ii) of the Spirulina influence on the lipid content of the meat (as a decreased lipid content would reduce the accumulation of the pyrethroid in the tissue) [67].

Staying on the subject of the effects of dietary Spirulina on the safety of broiler meat, it must be mentioned that some studies reported that the undesirable presence of potentially harmful levels of chemical contaminants, such as cyanotoxins (e.g., microcystins), heavy metals, pesticides, and polycyclic aromatic hydrocarbons, has been detected in various commercially available dietary supplements containing Spirulina [26,154]. Although these data refer to Spirulina products for human consumption, the same problem may also be expected to occur with Spirulina products for use in animal feed. Indeed, the microalga Spirulina can accumulate pollutants from the environment [18,20,26,27]. Moreover, although Spirulina is a non-toxic cyanobacterium, toxin-producing cyanobacterial species in the natural environment may coexist within the water used for culturing the microalga [154]. As a consequence, contamination of Spirulina biomass can easily take place under non-rigorous cultivation conditions [18,27], and the regular intake of contaminated Spirulina biomass by chickens through the feed during the whole period of rearing may pose significant health risks to the animals [23], as well as to the humans consuming their contaminated meat [51,52].

So, although Spirulina has the “generally recognized as safe” (GRAS) status [17,18], it is important to keep in mind that this safety only applies to Spirulina products with contaminant levels that do not exceed the tolerable values. In this view, it appears justified that some countries (like the European Union) with the priority to ensure consumer and animal protection pay particular attention to the quality of the algae-based products that enter their markets, striving to impose strict controls of their chemical composition and proper risk assessment of their production process [18,19,20].

### 4.4. Integrated Perspective with Economic Considerations: Opportunities and Challenges

***(a) Impact of dietary Spirulina on the physiology of broiler chicken production***

From the reviewed literature, it emerges that dietary inclusion of Spirulina can actually influence a large variety of parameters related to broiler productive performance, health status, and meat quality, either positively or negatively, depending mainly, though not exclusively, on the inclusion level. An overview of the various production-related aspects of broiler chickens’ physiology that Spirulina has been shown to modify when used as a feed supplement or a soybean replacer is provided in Figure 4, along with an indication of the mechanistic events that have been implicated in the chickens’ responses to dietary Spirulina.

Looking at the use of Spirulina as a feed functional supplement (i.e., at relatively low inclusion levels), there is a rather significant body of evidence that seems to confirm its ability to either promote or support the intestinal and extra-intestinal health status of broiler chickens, leading to improved or preserved productive performance of the birds (under normal or challenging conditions, respectively), as well as to increased disease resistance and survivability. A recently published meta-analysis of dietary Spirulina’s effects on broiler chickens’ growth performances corroborates part of this conclusion [155].

Moreover, at the relatively low intake levels that are used for feed supplementation, Spirulina seems able to also enhance the quality of the broiler meat, particularly in terms of nutritional value (enrichment with beneficial *n*-3 PUFAs, increased content of protein and essential amino acids, decreased content of fat, cholesterol, and triglycerides), oxidative stability, and microbiological quality, with potential (not yet fully explored) to yield a healthier food product with an extended shelf-life. At the highest levels of feed supplementation (>1%), the color of the broiler meat also might appear enhanced, however, to an extent that seems unlikely to impact consumer acceptability of the product significantly.

Based on the studies examined herein, the levels of Spirulina supplementation in the broiler diet that have successfully produced desirable performance-, health- and/or meat quality-related effects roughly range from 0.01 to 3–5%. Within this range, however, as a demonstration of the complexity of the relationship between Spirulina and changes induced in chicken physiology [51,52], it is possible to identify (i) supplementation levels that seem effective at producing some desirable changes but not others; (ii) supplementation levels that would produce certain desirable changes, of a certain desirable magnitude, in some circumstances, but not in others; and (iii) supplementation levels that, while producing a certain desirable change, of a certain desirable magnitude, also would exert a negative influence on other physiological parameters, potentially leading to undesirable effects that may limit their usability.

In this view, there is an essential need to identify the optimal level (or level range) of Spirulina supplementation of the broiler diet that would allow for obtaining the maximum positive influence on the largest possible number of performance-, health- and meat quality-related parameters. Furthermore, it is necessary to establish the most appropriate form (e.g., the whole dried alga, powder, or liquid extract), mode (via feed or drinking water), time (i.e., rearing phase), and duration of Spirulina administration. The identification of these core components of any effective nutritional (and pharmacological) manipulations of animal physiology would represent a key advance towards field application.

Future studies addressing this issue would benefit from adopting more standardized experimental conditions, given the considerable influence that factors such as chicken strain, basal diet composition, and chemical composition of the Spirulina product tested may have on the overall animal response to the microalgal supplement. With particular regard to the latter factor, it is hoped and strongly recommended that future published scientific papers provide a comprehensive analysis not only of the nutritional profile, but also of the main functional bioactive components and possible contaminants of the Spirulina product incorporated in the broiler diet. In addition, the process of optimization of Spirulina’s use as a functional feed supplement for broilers would benefit from more insights into the mechanisms underlying the desirable effects of Spirulina on chicken physiology, as well as into the identity of the specific bioactive compounds that play a most important role in producing these effects. This would also contribute to tailoring the use of this dietary supplement to the particular goal that has to be achieved and/or to the specific group of animals that has to be fed. Last but not least, more comparative studies are needed to assess how the efficacy of dietary Spirulina supplementation at improving/preserving the productive performance, health status, and/or meat quality of broiler chickens stands compared to other supplements with similar beneficial properties available in the market.

Regarding the use of Spirulina as an alternative protein source in the formulation of broiler diets, particularly as a partial or complete replacement of soybean-based feed ingredients, the relatively few studies conducted so far have considerably tempered the initial hopes by documenting the potential occurrence of negative impacts on the chickens’ growth performances starting from soybean replacement levels higher than 20–25% (with sensitivity to this effect seemingly varying among different chicken strains), and a consistently impaired growth performance when Spirulina replaces more than 55% of dietary soybean protein. The latter condition seems associated with important digestibility issues, and some strategies have been explored to overcome this limitation. Among them, extrusion processing of Spirulina, though not completely solving the problem, seems promising, and it seems reasonable to believe that its efficacy might be increased by concurrent supplementation of the extruded Spirulina-based diet with exogenous peptidase enzymes. The possibility of a “simpler” strategy, based on fortification of the diet with an extended level of amino acid supplementation for balancing the probably poor bioaccessibility of some valuable Spirulina proteins, also deserves consideration.

At the moderate to high dietary inclusion levels of Spirulina used for soybean protein replacement (≥3%; i.e., starting from soybean replacement levels > 10%), significant changes also seem to occur in broiler meat quality. Some of the reported changes may appear positive from a nutritional point of view, particularly the enrichment with the potent antioxidant carotenoids. However, it remains controversial how this impacts the oxidative stability of the meat, and also, it is unclear how the high intake levels of Spirulina modify the fatty acid composition of this meat. Moreover, with carotenoids also being pigments, their increased presence in the meat translates into an appreciable enhancement of meat color, which becomes particularly pronounced and describable as “dark reddish-yellowish” at the higher levels of Spirulina inclusion (≥10%) used to replace dietary soybean by 50% or more. In addition, at these higher levels, other modifications have been reported to involve meat sensory traits, particularly an increased chicken flavor. These color and flavor enhancement effects might not align with universal consumer preferences and might, therefore, limit the marketability of the meat obtained from broiler fed “soy-replaced” diets.

In light of this knowledge, further research is currently needed to determine the maximum level of soybean protein in broiler diets that may be replaced by Spirulina successfully, namely with no negative impact not only on the productive performance but also on consumer acceptability of the meat and, not less importantly, on chickens’ health. Moreover, it will be necessary to define the most appropriate rearing phase during which the substitution could be applied (starter, grower, finisher phase, or the whole rearing period, from day of hatching to slaughter age) and the most suitable strategy of dietary Spirulina inclusion for this specific in-feed application.

***(b) Impact of dietary Spirulina on the economics of broiler chicken production***

Even assuming that all of the critical “technical” issues highlighted above are entirely solved, the fact remains that the microalga Spirulina—at present—is relatively expensive, which is a limiting factor for its application in feed for broilers (and livestock in general) [3,18,20,29,33]. The high cost-price of Spirulina is mainly because of the high production costs, determined in turn by costs for construction and installation of the cultivation system (fixed capital expenditures) and the cost of all electricity consumed to keep the algae water circulating [1,14,17,18,19,20]. In addition, there are costs (in large part energy costs) related to the microalga post-harvest processing that is necessary to obtain a product suitable for inclusion in animal feed (e.g., drying, grinding, pelleting) [19]. Finally, additional costs to consider are those for exporting to ultimate end-user countries, given that the current production of Spirulina for the food and feed markets is quite concentrated on a relatively small number of non-European global producers (with China producing about two-thirds of the total Spirulina biomass produced worldwide) [17,21]. This latter aspect, combined with the limited volumetric productivity of open basins (the cultivation system most commonly used for commercial microalga production) [18], translates into still small global production volumes [3], making the final selling price particularly high [17,20]. Based on the online sale market, importing Spirulina for animal use in Europe would cost a minimum of EUR 11.80/kg for a minimum purchase quantity of 25 kg (the price includes value-added tax and is related to organic Spirulina powder, i.e., Spirulina of very high quality and grown under controlled conditions). The current market price for imported soybean meal, instead, is about EUR 0.42/kg, as determined by consultation (July 2024) of the Bologna Commodity Exchange (the price does not include value-added tax, and is related to wholemeal, foreign soybean meal, with 44% crude protein).

Therefore, under present circumstances, Spirulina cannot compete with conventional protein sources for livestock, and its possible use as a replacement for soybean meal in the formulation of broiler diet is far from being cost-effective (even the more so in consideration of the additional cost of the extrusion processing, exogenous enzyme supplementation and/or amino acid fortification necessarily required for this specific in feed application, as explained above) [14,18,20,29,53].

The Spirulina’s current cost-price is also relatively high compared with traditional growth-, health- and/or meat quality-improving feed additives for livestock [18,45,61]. However, in relation to this specific application, the higher cost of the microalga could be counterbalanced by the fact that only small quantities are used. This also explains why most of the global production volume of Spirulina for in-feed and in-food applications is currently sold as supplements [17,18].

With particular regard to the poultry farm context, a few of the studies reviewed herein have performed an economic analysis of the impact of including Spirulina as a functional supplement in the broiler diet (at levels ranging, on the whole, from 0.03% to 1%) [4,5,45,60,61]. Quite predictably, all of these studies reported an increase in the feed costs (and hence in the total cost of meat production) caused by the cost of the Spirulina supplement added to the ration, which was fairly proportional to the level of Spirulina supplementation realized. Nevertheless, all studies concluded that using Spirulina as a dietary supplement in a broiler diet (at the selected doses) can be cost-effective and recommendable, at least to the extent that the improvements in productive performance obtained through this supplementation translate into a corresponding increase in a gross return that is sufficient to achieve high enough net return increases.

It is of note, however, that based on the results of one of the abovementioned studies, the best economic efficiency is not necessarily recorded with the Spirulina supplementation levels that maximize the improvements in performance parameters (and that are usually the highest) [5]. Moreover, it should be pointed out that the calculations performed by the authors of all those studies did not consider the economic impact of the advantages that dietary Spirulina supplementation may produce in terms of reduced mortality rates and reduced medication use through its positive effects on chickens’ resistance to disease and stress. Finally, by exploiting the reported synergistic effects of Spirulina and other dietary supplements (e.g., garlic powder, selenium nanoparticles) [91,116,117], reducing the amount of Spirulina used and its associated costs may be possible.

In light of these economic considerations, it would be opportune that all future research efforts towards the identification of an “optimal” level (or level range) of Spirulina supplementation in broiler diets do not overlook the profitability of broiler farming, conducting a cost–benefit analysis of the data collected more regularly.

## 5. Concluding Remarks

In summary, the practical applicability of the microalga Spirulina as a component of broiler diets, at least for now, remains an open question, especially when considering its use as an alternative feed protein raw material for soybean replacement. This application still poses significant technical challenges and is economically unfeasible at current prices.

The future of Spirulina as a functional supplement seems more promising, or at least less uncertain. However, before this novel supplement can be included in the feed for broilers on a large scale, a large-scale commercial Spirulina production for the feed sector must occur [3,33]. In other terms, the global production volume of Spirulina (which is still too small) should increase to the point that it ceases to be a bottleneck [17]. This will require more applied research to bring innovation and further technological developments in microalgae cultivation systems, making them more productive and less energy-consuming [12,14,18,20,33].

The production up-scaling is also expected to reduce production costs substantially, as in microalgae production economies of scale play a significant role due to the large fixed capital expenditures [17]. This, in turn, will allow the selling prices of Spirulina to become more competitive with those of traditional feed additives and ingredients already present on the markets, making all potential applications of Spirulina in broiler feed (including soybean replacement) more affordable.

Meanwhile, as further scientific evidence is collected regarding the possibility of safely obtaining advantageous positive effects by dietary Spirulina supplementation on the productive performance, health status, and meat quality of broiler chickens, the overall perception of Spirulina as a nutritional asset will increase. In turn, this increased reputation will help increase the economic value of the microalga, as well as the interest and confidence of the broiler producer in its use, eventually leading to an increase in the global demand level for this product.

Scientists, therefore, play key role in guiding the further development of this market sector (as of many others). It is the responsibility of the whole scientific community to ensure that possible Spirulina transitioning into broiler production is based on reliable evidence collected through well-conducted research.

## Figures and Tables

**Table 1:** Nutritional and chemical composition of *Arthrospira* (*Spirulina*) *platensis*.

Item	Content ^(^*^)^	Unit of Measurement	Reference
**Energy**	1327–1603	kJ/100 g	[22,26]
**Dry matter**	88.1–96.0	% wet weight	[22,28,29]
**Carbohydrates (total)**	7.1–22.6	g/100 g dry matter	[14,22,24,25,26]
**Crude fiber**	0.5–7.0	g/100 g dry matter	[14,22,24,25,26,28,29]
**Crude protein**	35.4–70.0	g/100 g dry matter	[14,22,23,24,25,26,28,29]
**Amino Acid Composition**			[14,22,24,25,28,29]
***Essential amino acids***			
Arginine	3.8–4.9	g/100 g dry matter	
Histidine	0.9–2.7		
Isoleucine	3.1–4.4		
Leucine	4.8–8.4		
Lysine	2.6–4.6		
Methionine	1.2–2.8		
Phenylalanine	2.5–5.8		
Threonine	2.8–4.9		
Tryptophane	0.1–2.5		
Valine	3.3–4.7		
**Non-essential amino acids**			
Alanine	4.5–6.5	g/100 g dry matter	
Aspartate	2.4–9.2		
Cystine	0.4–0.7		
Glutamate	5.7–10.7		
Glycine	1.8–5.2		
Proline	2.0–4.0		
Serine	2.8–4.3		
Tyrosine	2.6–3.4		
**Crude fat**	0.6–16.0	g/100 g dry matter	[14,22,23,24,25,26,29]
**Fatty Acid Composition**			
Saturated fatty acids	49.2	% of total fatty acids	[22]
Monounsaturated fatty acids	8.9		
Polyunsaturated fatty acids	41.9		
n-6 fatty acids	40.4		
n-3 fatty acids	0.4		
n-6/n-3 fatty acids	101		
Myristic acid (14:0)	0.2–1.0		
Palmitic acid (16:0)	25.8–47.6		[22,24,32]
Palmitoleic acid (16:1c9)	2.3–13.5		[22,24,32]
Stearic acid (18:0)	0.5–3.4		[22,24,32]
Oleic acid (18:1c9)	1.0–16.6		[22,24,32]
Vaccenic (18:1c11)	0.21–0.73		[22]
Linoleic acid (18:2 n-6)	11.1–31.5		[22,24,32]
γ-linoleic acid (18:3 n-6)	12.9–40.1		[22,24,32]
Linolenic acid (18:3 n-3)	0.4		[22]
Arachidonic acid (20:0)	0.2		
Eicosapentaenoic acid (20:5 n-3)	<2.5	g/kg	[25]
Docosahexaenoic acid (22:6 n-3)	<3.0		
**Ash**	3.0–34.8	g/100 g dry matter	[14,19,22,23,25,29]
**Minerals**			
**Macrominerals**			
Calcium	0.23–14.0	g/kg	[19,22,24,25]
Magnesium	0.77–4.0		[19,22,24,25]
Phosphorous	1.2–22		[19,22,24,25]
Potassium	6.4–29.1		[19,22,24,25]
Sodium	4.5–96.2		[19,22,24,25]
**Microminerals**			
Copper	0.4–18.7	mg/kg	[19]
Iron	106.0–1800.0		[19,25]
Manganese	13.0–550.0		[19,25]
Zinc	0.4–40.0		[19,25]
**Vitamins, Pigments, Other Compounds**			
B1 (thiamine)	5–50	mg/kg	[14,22,25]
B2 (riboflavin)	30–46		[22,24]
B3 (niacin)	130–150		[22,24]
B6	4–50		[14,22,24,25]
B9 (folic acid)	0.3–99.2		[14,22,24,25,26]
B12	0.06–3.1		[22,24,26,30]
Vitamin K	22.0		[30]
α-tocopherol (vitamin E)	24.6–750		[14,22,25,26]
Carotenoids (total)	0.3–26	g/kg dry matter	[22,24,30]
β-carotene	0.02–2.3		[22,26,30]
Chlorophylls (total)	1.2	g/kg dry matter	[22]
Chlorophyll a	1.2–10.8		[22,30]
Chlorophyll b	0.04		[22]
C-phycocyanin	94.9–251.2	g/kg dry matter	[24,30]
Allophycocyanin	23.0		[24]
Phenols (total)	2.0–17.3	g/kg dry matter	
	1.3–6.4	µmol GAE/g (in carotenoids extracts)	[30]
Flavonoids (total)	1.0–9.0	g/kg dry matter	[24]
	12.9–26.6	µmol QE/g (in carotenoids extracts)	[30]
Polysaccharides	2.0–125.0	g/kg dry matter	[24]

^(^*^)^ The range reflects variations occurring in the amount of each single component that are determined by differences in the microalga strain and/or in the cultivation conditions (e.g., temperature, geographical location, availability of sunlight) [14]. **Abbreviations**: GAE = gallic acid equivalent; QE = quercetin equivalent.

**Table 4:** Overview of the studies evaluating the effects of dietary Spirulina supplementation on the carcass performance of broiler chickens [with particular regard to the relative weight (r.w.) of the whole eviscerated or non-eviscerated carcass, meat cuts, abdominal fat pad, and internal non-immune organs].

Spirulina Level (%)	Carcass (Eviscerated or Not)	Muscles	Abdominal Fat	Internal Non-Immune Organs	Notes	Reference
0.03–0.05–0.07–0.09	-	↓ r.w. of half breast and half rear	↓ r.w. of abdominal fat	↔ r.w. of total giblets	The four levels were equieffective at producing the changes in the r.w. of the muscles; The changes in the r.w. of the abdominal fat pad were equally produced by the three higher levels (0.05, 0.07, and 0.09%).	[4]
0.1	↑ Dressing percentage	-	-	↓ r.w. of liver ↓ r.w. of gizzard ↔ r.w. of proventriculus ↔ r.w. of cecum and colon	-	[50]
0.1–0.2	↑ Carcass percentage	↑ r.w. of edible parts	↓ r.w. of abdominal fat	↔ r.w. of liver ↔ r.w. of gizzard ↔ r.w. of heart ↔ r.w. of giblets	Only the highest level (0.2%) was effective.	[60]
0.1–0.15–0.2	↑ Dressing percentage	-	↔ r.w. of abdominal fat	-	Only the highest level (0.2%) was effective.	[45]
0.2	↑ Dressing percentage	-	↔ r.w. of abdominal fat	↑ r.w. of liver ↔ r.w. of gizzard ↔ r.w. of proventriculus ↔ r.w. of heart	-	[61]
0.1–0.3–0.5	↑ Dressing percentage	↑ r.w. of breast muscle ↔ r.w. of thigh muscle	-	↔ r.w. of liver ↔ r.w. of gizzard ↔ r.w. of heart	The lowest level (0.1%) was not effective; The effects of the two higher levels (0.3 and 0.5%) were dose-dependent.	[41]
0.25–0.5–0.75–1	-	↔ r.w. of breast muscle	↔ r.w. of abdominal fat	↔ r.w. of liver ↔ r.w. of gizzard	-	[63]
0.25–0.5–1	↔ Dressing percentage	-	-	↔ r.w. of liver ↔ r.w. of gizzard ↔ r.w. of proventriculus ↔ r.w. of heart	-	[5]
1	↔ Dressing percentage	-	-	-	-	[65]
1–1.5	-	-	↔ r.w. of abdominal fat	↓ r.w. of liver ↔ r.w. of gizzard ↓ r.w. of small intestine ↔ r.w. of small intestine ↔ r.w. of heart	Both levels were equieffective at producing changes in the r.w. of the liver; Only the highest level (1.5%) produced changes in the r.w. of abdominal fat.	[46]
3	-	↔ r.w. of breast muscle	-	↔ r.w. of liver ↔ r.w. of gizzard ↔ r.w. of proventriculus ↔ r.w. of intestine	This study also evaluated the effect of supplementing the Spirulina-containing diet with the carbohydrate active enzyme xylanase.	[69]
1–2–3–4	↑ Dressing percentage	↑ r.w. of breast muscle ↔ r.w. of thigh muscle	-	-	All levels were equieffective at producing the effect.	[71]
1–2.5–5	↑ Carcass percentage	-	-	-	Only the intermediate (2.5%) level was effective.	[72]

The “↑ ”, “↓ ”, or “↔” arrows indicate that an increase, a decrease, or no change, respectively, was observed in the parameter with the experimental diet (containing Spirulina) compared with the standard broiler diet (control). Where two or more levels of Spirulina supplementation were tested, and only some proved effective, the effective ones appear underlined. **Abbreviations**: r.w. = relative weight (% of the pre-slaughter weight).

**Table 5:** Overview of the studies evaluating the effects of dietary Spirulina supplementation on various aspects of the intestinal health of broiler chickens.

Spirulina Level (%)	Intestinal Microbiota	Intestinal Mucosal Layer	Other Aspects	Notes	Reference
0.03–0.05–0.07–0.09	↑ Count of Lactobacilli ↓ count of Coliforms	-	-	The lowest level (0.03%) was not effective; The other higher levels (0.05, 0.07, and 0.09%) were equieffective in producing changes in the gut microbiota composition.	[4]
0.1	↑ Count of Lactobacilli ↓ Count of Coliforms	-	-	-	[59]
0.1–0.15–0.2	-	Improved histomorphometry: ↑ villus height ↑ goblet cell number	-	The effect was particularly evident at the highest level (0.2%).	[45]
0.1–0.3–0.5	↑ Count of Lactobacilli ↑ Count of Coliforms	↑ FABP2 gene expression ↓ iNOS protein expression ↔ IL-1β gene expression ↑ SOD and GPx activity Improved histomorphology: ↓ enteritis severity	-	The effect on Lactobacilli count linearly increased as the inclusion level increased; The effect on Coliform count was produced by the two higher levels (0.3 and 0.5%); The three levels were equieffective at increasing intestinal SOD activity and reducing iNOS expression; The effects on GPx activity and FABP2 expression were dose-dependent; The improved histomorphology was observed only at 0.3%.	[41]
0.25–0.5–0.75–1	↑ Count of Lactobacilli ↔ Count of Coliforms	-	↑ Apparent total tract digestibility ↓ Excreta ammonia gas content	The effects linearly increased (microbiota and digestibility) or decreased (ammonia) as the inclusion level increased.	[63]
0.5–1	↑ Count of Lactobacilli ↓ Count of Coliforms	-	-	The two levels were equieffective at producing changes in the gut microbiota composition.	[48]
1	↑ Count of Lactobacilli ↓ Count of Coliforms	-	↓ Intestinal pH	-	[64]
0.5–1–1.5	↑ Count of Lactobacilli ↓ Count of Coliforms ↓ Count of *Salmonella* spp.	-	-	The effect on Lactobacilli count linearly increased as the inclusion level increased; The three levels were equieffective at producing changes in the Coliform count.	[66]
3	-	↑ ZO1 gene expression ↑ SLC7A7 gene expression ↑ CD56 gene expression Unchanged histomorphometry: ↔ villus height ↔ crypt depth	-	This study also evaluated the relative expression of various other genes related to barrier function, immunity, transport function, antioxidant capacity, and detecting no significant changes.	[69]

The “↑ ”, “↓ ”, or “↔” arrows indicate that an increase, a decrease, or no change, respectively, was observed in the parameter with the experimental diet (containing Spirulina) compared with the standard broiler diet (control). Where two or more levels of Spirulina supplementation were tested, and only some proved effective, the effective ones appear underlined. **Abbreviations**: FABP2 = fatty acid-binding protein 2; iNOS = inducible nitric oxide synthetase; IL-1β = Interleukin-1beta; SOD = superoxide dismutase; GPx = glutathione peroxidase; ZO1 = Zonula occludens 1; SLC7A7 = Solute carrier family 7, member 7; CD56 = Cluster of differentiation 56.

**Table 9:** Overview of the studies evaluating the effects of dietary Spirulina supplementation on the health of the immune system of broiler chickens as assessed by specific response to vaccination and post-vaccination response to pathogen challenge.

Spirulina Level (%)	Response to Vaccination				Post-Vaccination Response to Pathogen Challenge	Notes	Reference
	H5N1 AI	H9N2 AI	IB	ND			
0.02–0.04	-	↑ Ab titers ↑ Monocyte phagocytic activity ↑ Serum lysozyme levels	-	-	**Challenge → virulent H9N1 AI virus**: ↑ Post-challenge anti-H9N1 virus Ab titers ↓ Severity of histopathological lesions (in spleen and bursa too) ↑ Monocyte phagocytic activity ↑ Serum lysozyme levels ↑ Serum NO levels	In this study, Spirulina was used in the form of an extract. In this study, SPF chickens were used. The enhancing effects on Ab response, phagocytic activity, and lysozyme release were dose-dependent. The two levels were equieffective at mitigating histopathological alterations, as well as at reducing NO levels.	[109]
0.03–0.05–0.07–0.09	↔ Ab titers		-	↔ Ab titers	-	In this study, Cobb chickens were used. In this study, the type of AI virus against which the vaccine was directed was not specified.	[4]
0.1	↔ Ab titers		↑ Ab titers	↑ Ab titers	-	In this study, Ross 308 chickens were used. In this study, the type of AI virus against which the vaccine was directed was not specified.	[59]
0.1–0.15–0.2	-	-	-	↑ Ab titers	-	In this study, Arbor Acres chickens were used. The effect on Ab response was dose-dependent.	[45]
0.05–0.1–0.15–0.2	-	-	-	↑ Ab titers	**Challenge → virulent ND virus** **(genotype VII):** ↓ Mortality rate ↓ Viral shedding titers	In this study, Spirulina was used in the form of an extract. In this study, SPF chickens were used. The highest level (0.2%) was the most effective at producing the effects.	[108]
0.1–0.3–0.5	↑ Ab titers	-	↓ Ab titers	↔ Ab titers	**Challenge → virulent ND virus** **(genotype VII):** ↔ Post-challenge anti-ND virus Ab titers ↓ Morbidity rate ↓ Mortality rate ↓ Severity of pathological lesions ↓ Viral shedding titers	In this study, Cobb chickens were used. Only the 0.3% level was effective at increasing Ab response to the anti-H5N1 vaccine. All three levels decreased the Ab response to the anti-IB vaccine; the lowest level (0.1%) was the most effective at producing this decrease. The highest level (0.5%) produced a significant decrease in the Ab response to the anti-ND vaccine on day 20 of age. The three levels were equieffective at mitigating the response to the challenge in terms of morbidity, mortality, and pathology. The highest level (0.5%) was the most effective at reducing viral shedding post-challenge.	[107]
0.5–1–1.5	-	-	-	↑ Ab titers	-	In this study, Vencobb chickens were used. The lowest level (0.5%) was not effective. The two higher levels (1 and 1.5%) were equieffective at producing this effect.	[66]

The “↑ ”, “↓ ”, or “↔” arrows indicate that the parameter increased, decreased, or did not change, respectively, with the experimental diet (containing Spirulina) compared with the standard broiler diet (control). **Abbreviations**: Ab = antibodies; NO = nitric oxide; AI = avian influenza; IB = infectious bronchitis; ND = Newcastle disease.

**Table 10:** Overview of the studies evaluating the effects of dietary Spirulina supplementation on the productive performance and health status of broiler chickens exposed to heat stress (physical challenge).

Spirulina Level (%)	HS-Affected Parameters Showing Improvement Relative to the Positive Control ^(1)^			HS-Affected or HS-Unaffected Parameters Showing Improvement Relative to the Negative Control ^(2)^	Notes	Reference
	Partial Mitigation of	Substantial (=Almost Complete) Mitigation of	Complete Mitigation of			
0.1	Altered intestinal morphometry (↓ villus height) Systemic antioxidant/oxidant imbalance (↑ serum MDA levels, ↓ serum TAC) Altered immune cell populations (↓ WBC count, ↑ heterophil %, ↓ lymphocyte %, ↑ H/L ratio) Impaired immune organ development (↓ bursa r.w., ↓ spleen r.w.) Impaired humoral immune function (↓ serum IgM and IgY levels; ↓ anti-NDV and ant-IBDV Ab titers)	Impaired growth performance (↓ FBW, ↓ EPEI) Increased mortality rate	Impaired growth performance (↑ FCR) Altered intestinal morphometry (↑ crypt depth, ↓ villus height/crypt depth ratio) Altered cecal microbiota composition (↑ Coliform count) Liver damage (↑ serum ALT and AST activity) Altered lipid metabolism (↑ serum total cholesterol, ↑ LDL-cholesterol, ↑ triglycerides) Impaired immune organ development (↓ thymus r.w.) Impaired kidney function (↑ serum creatinine levels) Increased stress (↑ serum corticosterone levels)	Cecal Lactobacilli count (complete mitigation of the HS-induced reduction and further increase over the negative control) Serum uric acid levels (complete mitigation of the HS-induced increase and further decrease below the negative control)	In this study, the protective efficacy of Spirulina was compared to that of garlic and also tested in combination with it.	[117]
0.5–1–1.5	Systemic antioxidant/oxidant imbalance (↑ serum MDA levels)	Impaired growth performance (↓ FBW, ↓ BWG, ↑ FCR, ↓ EBI) Systemic antioxidant/oxidant imbalance (↓ serum GSH levels, ↓ serum TAC) Altered lipid metabolism (↑ serum total cholesterol)	Impaired carcass performance (↓ dressing %, ↓ breast r.w., ↓ fat r.w.) Liver damage (↑ serum AST activity) Altered lipid metabolism (↑ triglycerides) Impaired kidney function (↑ serum creatinine levels)	Intestine r.w. (complete mitigation of the HS-induced decrease, and further increase over the negative control) Serum SOD activity (complete mitigation of the HS-induced decrease and further increase over the negative control) Serum LDL-cholesterol levels (complete mitigation of the HS-induced increase and further decrease below the negative control) Serum urea levels (complete mitigation of the HS-induced increase and further decrease below the negative control)	The intermediate level (1%) was the most effective at counteracting the negative effects of HS on growth performance and some carcass-related parameters. The three levels were equieffective at counteracting the negative effects of HS on most of the other parameters.	[118]
3	Impaired growth performance (↓ BWG, ↓ ADFI) Altered intestinal morphometry (↑ crypt depth, ↓ villus height/crypt depth ratio) Altered cecal microbiota composition (↓ microbial richness and diversity)	-	Impaired growth performance (↓ FBW) Intestinal stress and inflammation (↑ *HSF2* gene expression, ↑ *IL12* gene expression)	↓ FCR (unaffected by HS) ↑ Intestinal villus height (unaffected by HS) ↑ Intestinal expression of *GPX3*, *IL4* and *CLDN2* genes (unaffected by HS)	In this study, HS did not affect, and dietary Spirulina did not improve the immune functions (as assessed by measurement of plasma IgA and IgY levels).	[70]
0.5–1–2	Systemic antioxidant/oxidant imbalance (↓ serum SOD activity)	-	Systemic antioxidant/oxidant imbalance (↑ serum MDA levels, ↓ serum GPx activity)	↓ Serum total cholesterol (unaffected by HS) ↓ Serum total lipid (complete mitigation of the HS-induced increase and further decrease below the negative control) ↓ Serum triglycerides (complete mitigation of the HS-induced increase and further decrease below the negative control) H/L ratio (complete mitigation of the HS-induced increase and further decrease below the negative control) ↑ Ab titers anti-SRBC antigens (unaffected by HS)	In this study, HS did not affect growth performance. Only the highest level (2%) proved effective at counteracting the negative effects of HS on the antioxidant/oxidant balance. The lowest level (0.5%) completely counteracted the negative effects of HS on lipid metabolism; the two higher levels (1 and 2%) improved lipid metabolism over the negative control. The intermediate level (1%) completely counteracted the negative effects of HS on the H/L ratio; the other two levels (0.5 and 2%) improved H/L ratio over the negative control. The two higher levels (1 and 2%) improved the humoral immune response over the negative control.	[58]
**Spirulina Level** **(%)**	**Parameters Showing Improvement** **Relative to the Positive Control ^(1)^**			**Notes**		**Reference**
0.5–1	Growth performance (↑ FBW and ↑ BWG) Carcass performance (↑ dressing %) Systemic antioxidant/oxidant balance (↓ serum MDA levels, ↑ serum SOD activity, ↑ serum GPx activity) Lipid metabolism (↓ serum total cholesterol, ↓ LDL-cholesterol, ↓ triglycerides) Humoral immune response (↑ IgG, ↑ IgM, ↑ IgA)			No negative control was included in the study design. The effect on most parameters was dose-related. In this study, the protective efficacy of Spirulina was compared to that of selenium nanoparticles and also tested in combination with them.		[91]
0.5–1	Growth performance (↑ BWG, ↑ EPEF) Ileal microbiota composition (↑ Lactobacilli count, ↓ Coliform count) Systemic antioxidant/oxidant balance (↓ serum TBARS levels, ↑ serum SOD activity, ↑ serum GPx activity) Humoral immune response (↑ IgG, ↑ IgM, ↑ IgA)			No negative control was included in the study design. The effect on most parameters was dose-related. Spirulina supplementation was found to not influence the humoral immune response to the anti-NDV, -AIV and -IBDV vaccines. In this study, the protective efficacy of Spirulina was compared to that of selenium nanoparticles and also tested in combination with them.		[116]
0.1–0.2	Growth performance (↑ FBW, ↑ BWG, ↓ FCR) - *powder more effective than extract* Carcass performance (↑ dressing %) - *only with powder* Systemic antioxidant/oxidant balance (↓ serum MDA levels, ↑ serum SOD and GPx activity) - *powder more effective than extract* Lipid metabolism (↓ serum total cholesterol, ↓ LDL-cholesterol, ↓ triglycerides) Humoral immune response (↑ IgG, ↑ IgM, ↑ anti-NDV Ab titers) - *only with powder (IgG, anti-NDV Ab)* - *powder more effective than extract (IgM)*			No negative control was included in the study design. In this study, the protective efficacy of two Spirulina forms via two different modes of administration was compared (dried powder in feed versus aqueous extract in drinking water). The effect of dried powder on growth parameters was dose-related. The two levels of aqueous extract were equieffective on most parameters. The highest level of aqueous extract (0.2%) was not effective on parameters related to lipid metabolism. Dried powder was more effective than aqueous extract in reducing serum triglycerides. Aqueous extract was more effective than dried powder in reducing serum LDL-cholesterol. Dried powder and aqueous extract were equieffective in reducing serum total cholesterol.		[54]
0.5–1–1.5–2	Growth performance (↑ FBW, ↑ BWG) Liver integrity (↓ serum AST activity, ↓ serum ALT activity) Liver function (↑ serum total proteins, ↓ serum total cholesterol, ↓ serum triglycerides) Immune function (↑ cellular response to PHA-P, ↑ humoral response to anti-NDV vaccine)			No negative control was included in the study design. In this study, dried powder of Spirulina was suspended in drinking water. The study was conducted under natural high temperature conditions. The lowest level (0.5%) was the most effective at improving FBW. Only the lower levels (0.5 and 1%) were effective at improving BWG. The three higher levels (1, 1.5 and 2%) were almost equieffective at improving serum total protein concentrations. The two highest levels (1.5 and 2%) were the most effective at improving all other health-related parameters.		[55]

The “↑ ”or “↓ ”arrows indicate that an increase or a decrease, respectively, was observed in the parameter with the experimental diet (Spirulina-containing) compared with the standard broiler diet (control). **^(1)^** Positive control: challenged (heat-stressed) chickens fed an unsupplemented diet; **^(2)^** negative control: non-challenged (non-heat-stressed) chickens fed an unsupplemented diet. **Abbreviations**: HS = heat stress; FBW = final body weight; BWG = body weight gain; ADFI = average daily feed intake; FCR = feed conversion ratio; EPEI = European production efficiency index; EBI = European broiler index; EPEF = European production efficiency factor; MDA = malondialdehyde; TAC = total antioxidant capacity; GSH = glutathione; SOD = superoxide dismutase; GPx = glutathione peroxidase; TBARS = thiobarbituric acid reactive substances; WBC = white blood cell; H/L = heterophil to lymphocyte ratio; Ab = antibodies; Ig = immunoglobulin; SRBC = sheep red blood cell; PHA-P = phytoheamagglutinin-P; r.w. = relative weight (% of the pre-slaughter weight); NDV = Newcastle disease virus; IBDV = infectious bursal disease virus; ALT = alanine aminotransferase; AST = aspartate aminotransferase; LDL = low-density lipoprotein; HSF2: heat shock factor 2; IL12: interleukin 12; GPX3: glutathione peroxidase 3; IL4: interleukin 4; CLDN2: Claudin 2.

**Table 12:** Levels of total dietary soybean replacement realized by the dietary inclusion of varying levels of Spirulina (total dietary soybean = soybean meal + other soy-based feed ingredients) in the studies evaluating the effect of feeding Spirulina-based diets on the productive performance of broiler chickens.

Soybean Replacement Level (%)	Spirulina Inclusion Level (%)	Feeding Phase	Reference
6.9	1.5	whole rearing cycle	[136]
8.6/9.6/11.2/12.3	2.5	whole rearing cycle (starter/grower/finisher 1/finisher 2)	[137]
13.8	3	whole rearing cycle	[136]
17.5	6	starter	[56]
16.7	3	grower + finisher	[53]
20.2	3	finisher	[53]
27.1	4	-	[138]
27.1/27.6	5	starter/grower	[139]
27.6	6	whole rearing cycle	[136]
33.3	6	grower + finisher	[53]
34.8	11	starter	[56]
40.4	6	finisher	[53]
49.6	15	-	[95,140]
50	11.8–9.7	starter/grower	[134]
52.2	16	starter	[56]
55.2	12	whole rearing cycle	[136]
55.4	8	-	[138]
59.9/56.0	10	starter/grower	[139]
60.9	15	-	[141]
69.8	21	starter	[56]
81.9/83.5	15	starter/grower	[139]

**Table 14:** Overview of the studies evaluating the effects of dietary Spirulina (used as either a feed supplement or a soybean replacer) on the physicochemical properties and sensory attributes of broiler chickens’ meat.

SP Inclusion Level (%)	Soybean Replacement Level (%)	Color	pH	WHC	Sensory Attributes	Notes	Reference
0.1	-	-	-	↓ Cooking loss ↔ Thawing loss	-	-	[47]
0.25–0.5–0.75–1	-	↔	↔	↔ Cooking loss ↓ 7-day drip loss	-	The effect on drip loss was dose-related.	[63]
1–2.5–5	3.1–9.4–18.8	Breast: ↑ b* ↔ L*, a*	↔	-	↔ Tenderness, juiciness, odor, flavor	The effect on meat yellowness was observed only with the two higher supplementation levels (2.5 and 5%) and did not reault in any visible change in color.	[72]
4–8	27.1–55.4	↑ a*, b*	-	-	-	The yellowness increased with increasing Spirulina inclusion levels and showed correlation with zeaxanthin meat content.	[138]
3 (f)–6 (f)	20.2–40.4	↑ a*, b*	↔	↔ Cooking loss ↓ Drip loss	-	The effect on meat color was recorded under all feeding regimens. The effect on meat WHC was recorded when 6% of Spirulina was used to replace soybean in both the grower and finisher diets.	[53]
3 (gf)–6 (gf)	16.7–33.3						
15	60.9	Breast and thigh ↑ b* Thigh: ↑ a* ↔ L*	↔	-	↔ Tenderness, flavor, off-flavors, overall acceptability ↓ Juiciness	In this study, the effects of dietary Spirulina were also evaluated in combination with carbohydrate-active enzymes (lysozyme or a mixture of xylanase and β-glucanase).	[141]
15	49.6	Breast and thigh: ↑ b* Thigh: ↑ a*	Breast: ↓ pH_24h_	-	-	In this study, the effects of dietary Spirulina were also evaluated after extrusion processing of Spirulina or in combination with a mixture of lysozyme and pancreatin.	[140]
11.8 (s)–9.7 (g)	50	↑ a*, b* ↓ L*	↑ pH_24h_	↓ Storage loss ↓ Cooking loss	↑ Tenderness ↓ Metallic off-flavor	This study was performed in Ross 308 broiler chickens. The feeding trial lasted 35 days and was arranged in two feeding phases (starter and grower). The meat showed dark reddish-yellowish color.	[144]
22.1 (s)	75	↑ a*, b*	-	-	↑ Umami and chicken flavor	This study was performed in Ross 308 broiler chickens. The feeding trial lasted 35 days and was arranged in two feeding phases (starter and grower); the soybean replacement level was 75% and 50% in the starter and grower diet, respectively. The dark reddish-yellowish color of the meat was still appreciable by the end of storage in a highly oxygenated modified atmosphere.	[11]
12.5 (g)	50						
14 (FM)–17 (GNC)	-	↑ a*, b*	-	-	-	In this study, dietary Spirulina was used to replace protein sources other than soybean.	[40]

The “↑ ”, “↓ ”, or “↔” arrows indicate that the parameter increased, decreased, or did not change, respectively, with the experimental diet (containing Spirulina) compared with the standard broiler diet (control). **Abbreviations**: L* = lightness; a* = redness; b* = yellowness; WHC = water-holding capacity measured as drip, storage and/or cooking loss; FM = fishmeal; GNC = groundnut cake.

## References

[1] Martins C.F., Ribeiro D.M., Costa M., Coelho D., Alfaia C.M., Lordelo M., Almeida A.M., Freire J.P.B., Prates J.A.M. (2021). Using Microalgae as a Sustainable Feed Resource to Enhance Quality and Nutritional Value of Pork and Poultry Meat. Foods.

[2] Bondar A., Horodincu L., Solcan G., Solcan C. (2023). Use of *Spirulina platensis* and Curcuma longa as Nutraceuticals in Poultry. Agriculture.

[3] El-Shall N.A., Jiang S., Farag M.R., Azzam M., Al-Abdullatif A.A., Alhotan R., Dhama K., Hassan F.U., Alagawany M. (2023). Potential of *Spirulina platensis* as a feed supplement for poultry to enhance growth performance and immune modulation. Front. Immunol..

[4] Fathi M.A., Namra M.M.M., Ragab M.S., Aly M.M.M. (2018). Effect of dietary supplementation of *algae* meal (*Spirulina platensis*) as growth promoter on performance of broiler chickens. Egypt. Poult. Sci. J..

[5] Hassan R.I.M., Refaie M.S., El-Shoukary R.D., Rehan I.F., Zigo F., Karaffova V., Amer H.Y. (2022). Effect of Dietary Microalgae (*Spirulina platensis*) on Growth Performance, Ingestive Behavior, Hemato-Biochemical Parameters, and Economic Efficiency of Fayoumi Broilers. Life.

[6] Sugiharto S., Yudiarti T., Isroli I., Widiastuti E. (2018). Effect of feeding duration of *Spirulina platensis* on growth performance, haematological parameters, intestinal microbial population and carcass traits of broiler chicks. South Afr. J. Anim. Sci..

[7] Rafiq K., Tofazzal Hossain M., Ahmed R., Hasan M.M., Islam R., Hossen M.I., Shaha S.N., Islam M.R. (2021). Role of Different Growth Enhancers as Alternative to In-feed Antibiotics in Poultry Industry. Front. Vet. Sci..

[8] Abd El-Hack M.E., El-Saadony M.T., Elbestawy A.R., El-Shall N.A., Saad A.M., Salem H.M., El-Tahan A.M., Khafaga A.F., Taha A.E., AbuQamar S.F. (2022). Necrotic enteritis in broiler chickens: Disease characteristics and prevention using organic antibiotic alternatives—A comprehensive review. Poult. Sci..

[9] Abd El-Hack M.E., El-Saadony M.T., Shafi M.E., Alshahrani O.A., Saghir S.A.M., Al-Wajeeh A.S., Al-Shargi O.Y.A., Taha A.E., Mesalam N.M., Abdel-Moneim A.E. (2022). Prebiotics can restrict Salmonella populations in poultry: A review. Anim. Biotechnol..

[10] Saleh A.A., Hafez A., Amber K., Abdelhady A.Y., Salem H.M., Fathy M., Kamal M.A., Alagawany M., Alzawqari M.H. (2023). Drug-independent control strategy of clostridial infection in broiler chickens using anti-toxin environmentally friendly multienzymes. Sci. Rep..

[11] Altmann B.A., Wigger R., Ciulu M., Mörlein D. (2020). The effect of insect or microalga alternative protein feeds on broiler meat quality. J. Sci. Food Agric..

[12] Abdel-Wareth A.A.A., Williams A.N., Salahuddin M., Gadekar S., Lohakare J. (2024). Algae as an alternative source of protein in poultry diets for sustainable production and disease resistance: Present status and future considerations. Front. Vet. Sci..

[13] Alagawany M., Lestingi A., Abdelzaher H.A., Elnesr S.S., Madkour M., El-Baz F.K., Alfassam H.E., Rudayni H.A., Allam A.A., Abd El Hack M.E. (2024). Dietary supplementation with *Dunaliella salina* microalga promotes quail growth by altering lipid profile and immunity. Poult. Sci..

[14] Saadaoui I., Rasheed R., Aguilar A., Cherif M., Al Jabri H., Sayadi S., Manning S.R. (2021). Microalgal-based feed: Promising alternative feedstocks for livestock and poultry production. J. Anim. Sci. Biotechnol..

[15] Vrenna M., Peruccio P.P., Liu X., Zhong F., Sun Y. (2021). Microalgae as Future Superfoods: Fostering Adoption through Practice-Based Design Research. Sustainability.

[16] Terezinha Schneider A., Costa Deprá M., Rodrigues Dias R., Queiroz Zepka L., Jacob-Lopes E., Arunkumar K., Arun A., Raja R., Palaniappan R. (2023). Chapter 5—Microalgae as superfood. Algae Materials.

[17] Enzing C., Ploeg M., Barbosa M.J., Sijtsma L. (2014). Microalgae-Based Products for Food and Feed Sector: An Outlook for Europe.

[18] Spruijt J., Krimpen M.V., Weide R.V. (2016). Opportunities for Micro Algae as Ingredient in Animal Diets.

[19] Costa M.M., Spinola M.P., Prates J.A.M. (2024). Microalgae as an Alternative Mineral Source in Poultry Nutrition. Vet. Sci..

[20] Bature A., Melville L., Rahman K.M., Aulak P. (2022). Microalgae as feed ingredients and a potential source of competitive advantage in livestock production: A review. Livest. Sci..

[21] Chen J., Wang Y., Benemann J.R., Zhang X., Hu H., Qin S. (2016). Microalgal industry in China: Challenges and prospects. J. Appl. Phycol..

[22] Lestingi A. (2024). Alternative and Sustainable Protein Sources in Pig Diet: A Review. Animals.

[23] Sugiharto S. (2020). Nutraceutical aspects of Microalgae *Spirulina* and *Chlorella* on broiler chickens. Livest. Res. Rural. Dev..

[24] Finamore A., Palmery M., Bensehaila S., Peluso I. (2017). Antioxidant, Immunomodulating, and Microbial-Modulating Activities of the Sustainable and Ecofriendly *Spirulina*. Oxid. Med. Cell Longev..

[25] Madeira M.S., Cardoso C., Lopes P.A., Coelho D., Afonso C., Bandarra N.M., Prates J.A.M. (2017). Microalgae as feed ingredients for livestock production and meat quality: A review. Livest. Sci..

[26] Grosshagauer S., Kraemer K., Somoza V. (2020). The True Value of *Spirulina*. J. Agric. Food Chem..

[27] Kerna N., Nwokorie U., Ann M., Ortigas M.A.C., Chawla S., Pruitt K., Flores J., Holets H., Carsrud V., Waugh S. (2021). *Spirulina* Consumption: Concerns Regarding Contaminants and Uncommon but Possible Adverse Reactions and Interactions. EC Pharmacol. Toxicol..

[28] Alvarenga R.R., Rodrigues P.B., Cantarelli V., Zangeronimo M.G., Da Silva Junior J.W., Da Silva L.R., Moreira dos Santos L. (2011). Energy values and chemical composition of *Spirulina* (*Spirulina platensis*) evaluated with broilers. Braz. J. Anim. Sci..

[29] Mullenix G.J., Maynard C.J., Owens C.M., Rochell S.J., Bottje W.G., Brister R.D., Kidd M.T. (2022). *Spirulina platensis* meal inclusion effects on broilers fed a reduced protein diet. J. Appl. Poult. Res..

[30] Gentscheva G., Nikolova K., Panayotova V., Peycheva K., Makedonski L., Slavov P., Radusheva P., Petrova P., Yotkovska I. (2023). Application of *Arthrospira platensis* for Medicinal Purposes and the Food Industry: A Review of the Literature. Life.

[31] Agustini T.W., Suzery M., Sutrisnanto D., Ma’ruf W.F., Hadiyanto (2015). Comparative Study of Bioactive Substances Extracted from Fresh and Dried *Spirulina* sp.. Procedia Environ. Sci..

[32] Mühling M., Belay A., Whitton B.A. (2005). Variation in fatty acid composition of *Arthrospira* (*Spirulina*) *strains*. J. Appl. Phycol..

[33] Altmann B.A., Rosenau S. (2022). *Spirulina* as Animal Feed: Opportunities and Challenges. Foods.

[34] Khan Z., Bhadouria P., Bisen P.S. (2005). Nutritional and therapeutic potential of *Spirulina*. Curr. Pharm. Biotechnol..

[35] Wu Q., Liu L., Miron A., Klimova B., Wan D., Kuca K. (2016). The antioxidant, immunomodulatory, and anti-inflammatory activities of *Spirulina*: An overview. Arch. Toxicol..

[36] Bortolini D.G., Maciel G.M., Fernandes I.d.A.A., Pedro A.C., Rubio F.T.V., Branco I.G., Haminiuk C.W.I. (2022). Functional properties of bioactive compounds from *Spirulina* spp.: Current status and future trends. Food Chem. Mol. Sci..

[37] Nuhu A.A. (2013). *Spirulina* (*Arthrospira*): An Important Source of Nutritional and Medicinal Compounds. J. Mar. Sci..

[38] Holman B.W.B., Malau-Aduli A.E.O. (2013). *Spirulina* as a livestock supplement and animal feed. J. Anim. Physiol. Anim. Nutrition.

[39] Mullenix G.J., Greene E.S., Emami N.K., Tellez-Isaias G., Bottje W.G., Erf G.F., Kidd M.T., Dridi S. (2021). *Spirulina platensis* Inclusion Reverses Circulating Pro-inflammatory (Chemo)cytokine Profiles in Broilers Fed Low-Protein Diets. Front. Vet. Sci..

[40] Venkataraman L.V., Somasekaran T., Becker E.W. (1994). Replacement value of blue-green alga (*Spirulina platensis*) for fishmeal and a vitamin-mineral premix for broiler chicks. Br. Poult. Sci..

[41] Abdelfatah S.H., Yassin A.M., Khattab M.S., Abdel-Razek A.S., Saad A.H. (2024). *Spirulina platensis* as a growth booster for broiler; Insights into their nutritional, molecular, immunohistopathological, and microbiota modulating effects. BMC Vet. Res..

[42] OECD/FAO (2023). OECD-FAO Agricultural Outlook 2023–2032.

[43] Moher D., Liberati A., Tetzlaff J., Altman D.G., Group P. (2009). Preferred reporting items for systematic reviews and meta-analyses: The PRISMA statement. PLoS Med..

[44] Tricco A.C., Lillie E., Zarin W., O’Brien K.K., Colquhoun H., Levac D., Moher D., Peters M.D.J., Horsley T., Weeks L. (2018). PRISMA Extension for Scoping Reviews (PRISMA-ScR): Checklist and Explanation. Ann. Intern. Med..

[45] Khan S., Mobashar M., Mahsood F.K., Javaid S., Abdel-Wareth A.A., Ammanullah H., Mahmood A. (2020). *Spirulina* inclusion levels in a broiler ration: Evaluation of growth performance, gut integrity, and immunity. Trop. Anim. Health Prod..

[46] Sharmin F., Sarker N., Sarker M. (2020). Effect of Using Moringa oleifera and *Spirulina platensis* as Feed Additives on Performance, Meat Composition and Oxidative Stability and Fatty Acid Profiles in Broiler Chicken. J. Nutr. Food Sci..

[47] El-Bahr S., Shousha S., Shehab A., Khattab W., Ahmed-Farid O., Sabike I., El-Garhy O., Albokhadaim I., Albosadah K. (2020). Effect of Dietary Microalgae on Growth Performance, Profiles of Amino and Fatty Acids, Antioxidant Status, and Meat Quality of Broiler Chickens. Animals.

[48] Alwaleed E.A., El-Sheekh M., Abdel-Daim M.M., Saber H. (2021). Effects of *Spirulina platensis* and Amphora coffeaeformis as dietary supplements on blood biochemical parameters, intestinal microbial population, and productive performance in broiler chickens. Environ. Sci. Pollut. Res. Int..

[49] Bonos E., Kasapidou E., Kargopoulos A., Karampampas A., Christaki E., Florou-Paneri P., Nikolakakis I. (2016). *Spirulina* as a functional ingredient in broiler chicken diets. South Afr. J. Anim. Sci..

[50] Kaoud H.A. (2015). Effect of *Spirulina platensis* as a dietary supplement on broiler performance in comparison with prebiotics. Spec. J. Biol. Sci..

[51] Spinola M.P., Costa M.M., Prates J.A.M. (2024). Effect of Cumulative *Spirulina* Intake on Broiler Meat Quality, Nutritional and Health-Related Attributes. Foods.

[52] Spinola M.P., Costa M.M., Prates J.A.M. (2024). Analysing the Impact of *Spirulina* Intake Levels on Performance Parameters, Blood Health Markers and Carcass Traits of Broiler Chickens. Animals.

[53] Zampiga M., Laghi L., Soglia F., Piscitelli R., Dayan J., Petracci M., Bonaldo A., Sirri F. (2024). Partial substitution of soybean meal with Microalgae meal (*Arthrospira* spp.—*Spirulina*) in grower and finisher diets for broiler chickens: Implications on performance parameters, footpad dermatitis occurrence, breast meat quality traits, amino acid digestibility and plasma metabolomics profile. Poult. Sci..

[54] Elbaz A.M., Ahmed A.M.H., Abdel-Maqsoud A., Badran A.M.M., Abdel-Moneim A.-M.E. (2022). Potential ameliorative role of *Spirulina platensis* in powdered or extract forms against cyclic heat stress in broiler chickens. Environ. Sci. Pollut. Res..

[55] Kolluri G., Marappan G., Yadav A.S., Kumar A., Mariappan A.K., Tyagi J.S., Rokade J.J., Govinthasamy P. (2022). Effects of *Spirulina* (*Arthrospira platensis*) as a drinking water supplement during cyclical chronic heat stress on broiler chickens: Assessing algal composition, production, stress, health and immune-biochemical indices. J. Therm. Biol..

[56] Evans A.M., Smith D.L., Moritz J.S. (2015). Effects of *algae* incorporation into broiler starter diet formulations on nutrient digestibility and 3 to 21 d bird performance. J. Appl. Poult. Res..

[57] Qureshi M.A., Garlich J.D., Kidd M.T. (1996). Dietary *Spirulina platensis* enhances humoral and cell-mediated immune functions in chickens. Immunopharmacol. Immunotoxicol..

[58] Mirzaie S., Zirak-Khattab F., Hosseini S.A., Donyaei-Darian H. (2018). Effects of dietary *Spirulina* on antioxidant status, lipid profile, immune response and performance characteristics of broiler chickens reared under high ambient temperature. Asian-Australas. J. Anim. Sci..

[59] Kasmani F.B., Javaremi A.N., Ghazaghi M. (2023). Biodetoxification of Aflatoxin B1 by *Arthrospira platensis* in Broilers. J. Appl. Phycol..

[60] Abou-Zeid A.E., El-Damarawy S.Z., Mariey Y.A., El-Mansy M.M. (2015). Effect of Using *Spirulina platensis* and/or *Chlorella vulgaris algae* as Feed Additives on Productive Performance of Broiler Chicks. J. Anim. Poult. Prod..

[61] El-Sharnobey R.R., Atallah S.T., Saad A.H., El-Ktany E. (2023). Comparative Appraisal of Relative Economic Efficiency of *Spirulina*, Cinnamon oil and Citric Acid Dietary Supplementations and Their Effect on Growth Performance and Carcass Traits in Broiler chicken. J. Adv. Vet. Res..

[62] Jamil A.B.M., Akanda M., Rahman M.M., Hossain M., Islam M. (2015). Prebiotic competence of *Spirulina* on the production performance of broiler chickens. J. Adv. Vet. Anim. Res..

[63] Park J.H., Lee S.I., Kim I.H. (2018). Effect of dietary *Spirulina* (*Arthrospira*) *platensis* on the growth performance, antioxidant enzyme activity, nutrient digestibility, cecal microflora, excreta noxious gas emission, and breast meat quality of broiler chickens. Poult. Sci..

[64] Alaqil A.A., Abbas A.O. (2023). The Effects of Dietary *Spirulina platensis* is on Physiological Responses of Broiler Chickens Exposed to Endotoxin Stress. Animals.

[65] Ismita J., Islam K.M.S., Al-Mamun M., Debi M.R. (2022). Comparative efficacy of citric acid, *Spirulina platensis*, and their combination as alternatives to an antibiotic growth promoter on the performances of broilers. J. Adv. Vet. Anim. Res..

[66] Khadanga A., Sethy K., Samantaray S.M., Ray P., Panda N., Mishra S.K., Naik M., Tripathy S. (2023). Effect of *Spirulina* on growth, immunity, gut bacterial load and histopathology of broiler birds. Indian J. Anim. Sci..

[67] Ibrahim S.S., Elsabagh R., Allam A., Youssef G., Fadl S.E., Abdelhiee E.Y., Alkafafy M., Soliman A., Aboubakr M. (2021). Bioremediation role of *Spirulina platensis* against deltamethrin-mediated toxicity and its chemical residues in chicken meat. Environ. Sci. Pollut. Res. Int..

[68] Atiyah W.R., Hamood M.F. (2021). Enhancing the Productive Performance of Broiler Chickens by Adding *Spirulina platensis* Compared with Probiotic, Prebiotics, and Oxytetracycline. Iraqi J. Vet. Med..

[69] Mishra P., Das R., Chaudhary A., Mishra B., Jha R. (2023). Effects of Microalgae, with or without xylanase supplementation, on growth performance, organs development, and gut health parameters of broiler chickens. Poult. Sci..

[70] Chaudhary A., Mishra P., Amaz S.A., Mahato P.L., Das R., Jha R., Mishra B. (2023). Dietary supplementation of Microalgae mitigates the negative effects of heat stress in broilers. Poult. Sci..

[71] Abbass M.S., Bandar L.K., Hussein F.M. (2020). Effect of using different levels of *Spirulina algae* (*Spirulina platensis*) in diet on productive performance and characteristics of the carcass of broiler. Plant Arch..

[72] Raach-Moujahed A., Hassani S., Zairi S., Mahdi B., Darej C., Haddad B., Damergi C. (2011). Effect of dehydrated *Spirulina platensis* on performances and meat quality of broilers. Res. Opin. Anim. Vet. Sci..

[73] Boskovic Cabrol M., Glisic M., Almeida A.M., M Z Baltic Ž M., Raymundo A., Lordelo M.M. (2021). The effect of *Spirulina* inclusion in broiler feed on meat quality: Recent trends in sustainable production. IOP Conf. Ser. Earth Environ. Sci..

[74] El-Sheekh M.M., Daboor S.M., Swelim M.A., Mohamed S. (2014). Production and characterization of antimicrobial active substance from *Spirulina platensis*. Iran. J. Microbiol..

[75] Abdel-Moneim A.E., El-Saadony M.T., Shehata A.M., Saad A.M., Aldhumri S.A., Ouda S.M., Mesalam N.M. (2022). Antioxidant and antimicrobial activities of *Spirulina platensis* extracts and biogenic selenium nanoparticles against selected pathogenic bacteria and fungi. Saudi J. Biol. Sci..

[76] El-Ghany W. (2020). Microalgae in Poultry Field: A Comprehensive Perspectives. Adv. Anim. Vet. Sci..

[77] Cornick S., Tawiah A., Chadee K. (2015). Roles and regulation of the mucus barrier in the gut. Tissue Barriers.

[78] Grondin J.A., Kwon Y.H., Far P.M., Haq S., Khan W.I. (2020). Mucins in Intestinal Mucosal Defense and Inflammation: Learning From Clinical and Experimental Studies. Front. Immunol..

[79] Šefcová M.A., Santacruz F., Larrea-Álvarez C.M., Vinueza-Burgos C., Ortega-Paredes D., Molina-Cuasapaz G., Rodríguez J., Calero-Cáceres W., Revajová V., Fernández-Moreira E. (2021). Administration of Dietary Microalgae Ameliorates Intestinal Parameters, Improves Body Weight, and Reduces Thawing Loss of Fillets in Broiler Chickens: A Pilot Study. Animals.

[80] Blaiotta G., Murru N., Di Cerbo A., Romano R., Aponte M. (2018). Production of probiotic bovine salami using Lactobacillus plantarum 299v as adjunct. J. Sci. Food Agric..

[81] Di Cerbo A., Palmieri B., Aponte M., Morales-Medina J.C., Iannitti T. (2016). Mechanisms and therapeutic effectiveness of lactobacilli. J. Clin. Pathol..

[82] Ricciardi A., Blaiotta G., Di Cerbo A., Succi M., Aponte M. (2014). Behaviour of lactic acid bacteria populations in Pecorino di Carmasciano cheese samples submitted to environmental conditions prevailing in the gastrointestinal tract: Evaluation by means of a polyphasic approach. Int. J. Food Microbiol..

[83] Romano A., Blaiotta G., Di Cerbo A., Coppola R., Masi P., Aponte M. (2014). Spray-dried chestnut extract containing *Lactobacillus rhamnosus* cells as novel ingredient for a probiotic chestnut mousse. J. Appl. Microbiol..

[84] Di Cerbo A., Palmieri B. (2015). Review: The market of probiotics. Pak. J. Pharm. Sci..

[85] Di Cerbo A., Palmieri B. (2013). Lactobacillus Paracasei subsp. Paracasei F19; a farmacogenomic and clinical update. Nutr. Hosp..

[86] Koh A., De Vadder F., Kovatcheva-Datchary P., Bäckhed F. (2016). From Dietary Fiber to Host Physiology: Short-Chain Fatty Acids as Key Bacterial Metabolites. Cell.

[87] Omar A.E., Al-Khalaifah H.S., Osman A., Gouda A., Shalaby S.I., Roushdy E.M., Abdo S.A., Ali S.A., Hassan A.M., Amer S.A. (2022). Modulating the Growth, Antioxidant Activity, and Immunoexpression of Proinflammatory Cytokines and Apoptotic Proteins in Broiler Chickens by Adding Dietary *Spirulina platensis* Phycocyanin. Antioxidants.

[88] Tsai T.Y.-C., Garner R.M., Megason S.G. (2022). Adhesion-Based Self-Organization in Tissue Patterning. Annu. Rev. Cell Dev. Biol..

[89] Wang Y., Tibbetts S.M., McGinn P.J. (2021). Microalgae as Sources of High-Quality Protein for Human Food and Protein Supplements. Foods.

[90] Estevez M. (2015). Oxidative damage to poultry: From farm to fork. Poult. Sci..

[91] Abdel-Moneim A.E., Shehata A.M., Mohamed N.G., Elbaz A.M., Ibrahim N.S. (2022). Synergistic effect of *Spirulina platensis* and selenium nanoparticles on growth performance, serum metabolites, immune responses, and antioxidant capacity of heat-stressed broiler chickens. Biol. Trace Elem. Res..

[92] Prasad A.S., Bao B. (2019). Molecular Mechanisms of Zinc as a Pro-Antioxidant Mediator: Clinical Therapeutic Implications. Antioxidants.

[93] Chasapis C.T., Ntoupa P.A., Spiliopoulou C.A., Stefanidou M.E. (2020). Recent aspects of the effects of zinc on human health. Arch. Toxicol..

[94] El-Kholy M.S., El-Mekkawy M.M., Madkour M., Abd El-Azeem N., Di Cerbo A., Mohamed L.A., Alagawany M., Selim D.A. (2023). The role of different dietary Zn sources in modulating heat stress-related effects on some thermoregulatory parameters of New Zealand white rabbit bucks. Anim. Biotechnol..

[95] Spínola M.P., Alfaia C.M., Costa M.M., Pinto R.M.A., Lopes P.A., Pestana J.M., Tavares J.C., Mendes A.R., Mourato M.P., Tavares B. (2024). Impact of high *Spirulina* diet, extruded or supplemented with enzymes, on blood cells, systemic metabolites, and hepatic lipid and mineral profiles of broiler chickens. Front. Vet. Sci..

[96] Deng R., Chow T.J. (2010). Hypolipidemic, antioxidant, and antiinflammatory activities of Microalgae *Spirulina*. Cardiovasc. Ther..

[97] DiNicolantonio J.J., Bhat A.G., Okeefe J. (2020). Effects of *Spirulina* on weight loss and blood lipids: A review. Open Heart.

[98] Abdel-Moneim A.-M.E., Shehata A.M., Alzahrani S.O., Shafi M.E., Mesalam N.M., Taha A.E., Swelum A.A., Arif M., Fayyaz M., Abd El-Hack M.E. (2020). The role of polyphenols in poultry nutrition. J. Anim. Physiol. Anim. Nutr..

[99] El-Abd N.M., Hamouds R.A., Saddiq A.A., Al-Shaikh T.M., Khusaifan T.J., Abou-El-Souod G. (2024). Effect of dietary *Arthrospira platensis* phycocyanin on broiler chicken growth performance, physiological status, fatty and amino acid profiles. Vet. World.

[100] Balachandran P., Pugh N.D., Ma G., Pasco D.S. (2006). Toll-like receptor 2-dependent activation of monocytes by *Spirulina* polysaccharide and its immune enhancing action in mice. Int. Immunopharmacol..

[101] Pugh N.D., Edwall D., Lindmark L., Kousoulas K.G., Iyer A.V., Haron M.H., Pasco D.S. (2015). Oral administration of a *Spirulina* extract enriched for Braun-type lipoproteins protects mice against influenza A (H1N1) virus infection. Phytomedicine.

[102] Li Y., Ji N., Wang M., Pugh N.D., Khan I.A., Tan C. (2023). Immulina as an Immunostimulatory Supplement: Formulation and Pharmacological Studies. Planta Med..

[103] Al-Batshan H.A., Al-Mufarrej S.I., Al-Homaidan A.A., Qureshi M.A. (2001). Enhancement of chicken macrophage phagocytic function and nitrite production by dietary *Spirulina platensis*. Immunopharmacol. Immunotoxicol..

[104] Katayama S., Kayahara Y., Watanabe T. (2016). Enhancement of Immunological Responses by Dietary *Arthrospira platensis* and Possibility of Field Applications as Alternative to Antibiotics in Broiler Chicken. Am. J. Anim. Vet. Sci..

[105] Lokapirnasari W.P., Yulianto A.B., Legowo D., Agustono (2016). The Effect of *Spirulina* as Feed Additive to Myocardial Necrosis and Leukocyte of Chicken with Avian Influenza (H5N1) Virus Infection. Procedia Chem..

[106] Kumari P., Kundu P., Kajal S., Narang G. (2019). Effect of *Spirulina* feeding on serum protein level in Infectious Bursal Disease vaccinated chickens. Pharma Innov. J..

[107] Awad A.M., Sedeik M.E., Salaheldin A.H., Goda R.I., El-Shall N.A. (2023). Evaluating the effect of *Spirulina platensis* on the immune response of broiler chickens to various vaccines and virulent Newcastle disease virus challenge. Res. Vet. Sci..

[108] Abotaleb M.M., Mourad A., Abousenna M.S., Helal A.M., Nassif S.A., Elsafty M.M. (2020). The effect of Spirulina algae on the immune response of SPF chickens to commercial inactivated Newcastle vaccine in poultry. VacciMonitor.

[109] Yehia N., Mohamed F.H., Al-Zaban M.I., Amer F., Baazaoui N., Khattab M.S., Abd Elhalem Mohamed A., Salem H.M., El-Saadony M.T., El-Tarabily K.A. (2024). The influence of *Spirulina* extract on pathogenicity, immune response, and vaccine efficacy against H9N2 avian influenza virus in specific pathogen free chickens. Poult. Sci..

[110] Beutler B. (2004). Innate immunity: An overview. Mol. Immunol..

[111] Kim D.H., Park M.H., Choi Y.J., Chung K.W., Park C.H., Jang E.J., An H.J., Yu B.P., Chung H.Y. (2013). Molecular study of dietary heptadecane for the anti-inflammatory modulation of NF-kB in the aged kidney. PLoS ONE.

[112] Ku C.S., Pham T.X., Park Y., Kim B., Shin M.S., Kang I., Lee J. (2013). Edible blue-green *algae* reduce the production of pro-inflammatory cytokines by inhibiting NF-kappaB pathway in macrophages and splenocytes. Biochim. Biophys. Acta.

[113] Morris K.R., Lutz R.D., Choi H.S., Kamitani T., Chmura K., Chan E.D. (2003). Role of the NF-kappaB signaling pathway and kappaB cis-regulatory elements on the IRF-1 and iNOS promoter regions in mycobacterial lipoarabinomannan induction of nitric oxide. Infect. Immun..

[114] Bi Y., Wu D., Wu X., Wang F., Yu H., Liu P., Cui G., Chen Z. (2022). Phycocyanin inhibits Helicobacter pylori-induced hyper-proliferation in AGS cells via activation of the ROS/MAPK signaling pathway. Ann. Transl. Med..

[115] Chen J.C., Liu K.S., Yang T.J., Hwang J.H., Chan Y.C., Lee I.T. (2012). *Spirulina* and C-phycocyanin reduce cytotoxicity and inflammation-related genes expression of microglial cells. Nutr. Neurosci..

[116] Abdel-Moneim A.E., Shehata A.M., Selim D.A., El-Saadony M.T., Mesalam N.M., Saleh A.A. (2022). *Spirulina platensis* and biosynthesized selenium nanoparticles improve performance, antioxidant status, humoral immunity and dietary and ileal microbial populations of heat-stressed broilers. J. Therm. Biol..

[117] Attia Y.A., Hassan R.A., Addeo N.F., Bovera F., Alhotan R.A., Al-qurashi A.D., Al-Baadani H.H., Al-Banoby M.A., Khafaga A.F., Eisenreich W. (2023). Effects of *Spirulina platensis* and/or Allium sativum on Antioxidant Status, Immune Response, Gut Morphology, and Intestinal Lactobacilli and Coliforms of Heat-Stressed Broiler Chicken. Vet. Sci..

[118] Moustafa E.S., Alsanie W.F., Gaber A., Kamel N.N., Alaqil A.A., Abbas A.O. (2021). Blue-Green Algae (*Spirulina platensis*) Alleviates the Negative Impact of Heat Stress on Broiler Production Performance and Redox Status. Animals.

[119] Hirakawa R., Nurjanah S., Furukawa K., Murai A., Kikusato M., Nochi T., Toyomizu M. (2020). Heat Stress Causes Immune Abnormalities via Massive Damage to Effect Proliferation and Differentiation of Lymphocytes in Broiler Chickens. Front. Vet. Sci..

[120] Quinteiro-Filho W.M., Calefi A.S., Cruz D.S.G., Aloia T.P.A., Zager A., Astolfi-Ferreira C.S., Piantino Ferreira J.A., Sharif S., Palermo-Neto J. (2017). Heat stress decreases expression of the cytokines, avian beta-defensins 4 and 6 and Toll-like receptor 2 in broiler chickens infected with Salmonella Enteritidis. Vet. Immunol. Immunopathol..

[121] Shehata A.M., Saadeldin I.M., Tukur H.A., Habashy W.S. (2020). Modulation of Heat-Shock Proteins Mediates Chicken Cell Survival against Thermal Stress. Animals.

[122] Bhowmik D., Dubey J., Mehra S. (2009). Probiotic Efficiency of *Spirulina platensis*—Stimulating Growth of Lactic Acid Bacteria. Am. Eurasian J. Agric. Environ. Sci..

[123] Raju M.V.L.N., Rao S.V.R., Radhika K., Chawak M.M. (2005). Dietary supplementation of *Spirulina* and its effects on broiler chicken exposed to aflatoxicosis. Indian J. Poult. Sci..

[124] Feshanghchi M., Baghban-Kanani P., Kashefi-Motlagh B., Adib F., Azimi-Youvalari S., Hosseintabar-Ghasemabad B., Slozhenkina M., Gorlov I., Zangeronimo M.G., Swelum A.A. (2022). Milk Thistle (*Silybum marianum*), Marine Algae (*Spirulina platensis*) and Toxin Binder Powders in the Diets of Broiler Chickens Exposed to Aflatoxin-B1: Growth Performance, Humoral Immune Response and Cecal Microbiota. Agriculture.

[125] Rashidi N., Khatibjoo A., Taherpour K., Akbari-Gharaei M., Shirzadi H. (2020). Effects of licorice extract, probiotic, toxin binder and poultry litter biochar on performance, immune function, blood indices and liver histopathology of broilers exposed to aflatoxin-B(1). Poult. Sci..

[126] Abdelnour S.A., Mahasneh Z.M.H., Barakat R.A., Alkahtani A.M., Madkour M. (2024). Microalgae: A promising strategy for aflatoxin control in poultry feeds. Toxicon.

[127] Alpsoy L., Yalvac M.E. (2011). Key roles of vitamins A, C, and E in aflatoxin B1-induced oxidative stress. Vitam. Horm..

[128] Simonich M.T., Egner P.A., Roebuck B.D., Orner G.A., Jubert C., Pereira C., Groopman J.D., Kensler T.W., Dashwood R.H., Williams D.E. (2007). Natural chlorophyll inhibits aflatoxin B1-induced multi-organ carcinogenesis in the rat. Carcinogenesis.

[129] Talha M.M.H., Hossain M.A., Aktaruzzaman M., Islam M.S., Khasnobish A., Akanda M.R. (2022). Effects of *Spirulina* as a functional ingredient in arsenic-induced broiler diet on growth performance and hematobiochemical parameters. J. Adv. Vet. Anim. Res..

[130] Bharavi K., Reddy A.G., Rao G.S., Kumar P.R., Kumar D.S., Prasadini P.P. (2011). Prevention of cadmium bioaccumulation by herbal adaptogens. Indian. J. Pharmacol..

[131] Bhattacharya S. (2020). The Role of *Spirulina* (*Arthrospira*) in the Mitigation of Heavy-Metal Toxicity: An Appraisal. J. Environ. Pathol. Toxicol. Oncol..

[132] Bermejo P., Piñero E., Villar Á.M. (2008). Iron-chelating ability and antioxidant properties of phycocyanin isolated from a protean extract of *Spirulina platensis*. Food Chem..

[133] Abdel-Daim M., El-Bialy B.E., Rahman H.G.A., Radi A.M., Hefny H.A., Hassan A.M. (2016). Antagonistic effects of *Spirulina platensis* against sub-acute deltamethrin toxicity in mice: Biochemical and histopathological studies. Biomed. Pharmacother..

[134] Velten S., Neumann C., Bleyer M., Gruber-Dujardin E., Hanuszewska M., Przybylska-Gornowicz B., Liebert F. (2018). Effects of 50 Percent Substitution of Soybean Meal by Alternative Proteins from Hermetia illucens or *Spirulina platensis* in Meat-Type Chicken Diets with Graded Amino Acid Supply. Open J. Anim. Sci..

[135] Manceron S., Ben-Ari T., Dumas P. (2014). Feeding proteins to livestock: Global land use and food vs. feed competition. Oilseeds Fats Crops Lipids.

[136] Ross E., Dominy W. (1990). The Nutritional Value of Dehydrated, Blue-Green Algae (*Spirulina plantensis*) for Poultry1. Poult. Sci..

[137] Meyer M.M., Johnson A.K., Bobeck E.A. (2021). Laser Environmental Enrichment and *Spirulina* Algae Improve Broiler Growth Performance and Alter Myogenic Gene Expression and pectoralis major Dimensions. Front. Anim. Sci..

[138] Toyomizu M., Sato K., Taroda H., Kato T., Akiba Y. (2001). Effects of dietary *Spirulina* on meat colour in muscle of broiler chickens. Br. Poult. Sci..

[139] Zampiga M., Brugaletta G., Ceccaroni F., Bonaldo A., Pignata S., Sirri F. (2023). Performance response of broiler chickens fed diets containing dehydrated *Microalgae* meal as partial replacement for soybean until 22 days of age. Anim. Feed. Sci. Technol..

[140] Costa M.M., Spinola M.P., Tavares B., Pestana J.M., Tavares J.C., Martins C.F., Alfaia C.M., Carvalho D.F.P., Mendes A.R., Ferreira J.I. (2024). Effects of high dietary inclusion of *Arthrospira platensis*, either extruded or supplemented with a super-dosing multi-enzyme mixture, on broiler growth performance and major meat quality parameters. BMC Vet. Res..

[141] Pestana J.M., Puerta B., Santos H., Madeira M.S., Alfaia C.M., Lopes P.A., Pinto R.M.A., Lemos J.P.C., Fontes C., Lordelo M.M. (2020). Impact of dietary incorporation of *Spirulina* (*Arthrospira platensis*) and exogenous enzymes on broiler performance, carcass traits, and meat quality. Poult. Sci..

[142] Velten S., Neumann C.J., Schäfer J., Liebert F. (2018). Effects of the Partial Replacement of Soybean Meal by Insect or Algae Meal in Chicken Diets with Graded Amino Acid Supply on Parameters of Gut Microbiology and Dietary Protein Quality. Open J. Anim. Sci..

[143] Wood J.D., Richardson R.I., Nute G.R., Fisher A.V., Campo M.M., Kasapidou E., Sheard P.R., Enser M. (2004). Effects of fatty acids on meat quality: A review. Meat Sci..

[144] Altmann B.A., Neumann C., Velten S., Liebert F., Morlein D. (2018). Meat Quality Derived from High Inclusion of a Micro-Alga or Insect Meal as an Alternative Protein Source in Poultry Diets: A Pilot Study. Foods.

[145] Gkarane V., Ciulu M., Altmann B., Morlein D. (2020). Effect of Alternative Protein Feeds on the Content of Selected Endogenous Bioactive and Flavour-Related Compounds in Chicken Breast Meat. Foods.

[146] Mendiola J.A., García-Martínez D., Rupérez F.J., Martín-Álvarez P.J., Reglero G., Cifuentes A., Barbas C., Ibañez E., Señoráns F.J. (2008). Enrichment of vitamin E from *Spirulina platensis* microalga by SFE. J. Supercrit. Fluids.

[147] Adhihetty P.J., Beal M.F. (2008). Creatine and its potential therapeutic value for targeting cellular energy impairment in neurodegenerative diseases. Neuromolecular Med..

[148] Volek J.S., Rawson E.S. (2004). Scientific basis and practical aspects of creatine supplementation for athletes. Nutrition.

[149] Ribas-Agusti A., Diaz I., Sarraga C., Garcia-Regueiro J.A., Castellari M. (2019). Nutritional properties of organic and conventional beef meat at retail. J. Sci. Food Agric..

[150] Kalia S., Lei X.G. (2022). Dietary Microalgae on poultry meat and eggs: Explained versus unexplained effects. Curr. Opin. Biotechnol..

[151] Bejaoui B., Sdiri C., Ben Souf I., Belhadj Slimen I., Ben Larbi M., Koumba S., Martin P., M’Hamdi N. (2023). Physicochemical Properties, Antioxidant Markers, and Meat Quality as Affected by Heat Stress: A Review. Molecules.

[152] Gkarane V., Ciulu M., Altmann B.A., Schmitt A.O., Mörlein D. (2020). The Effect of Algae or Insect Supplementation as Alternative Protein Sources on the Volatile Profile of Chicken Meat. Foods.

[153] Grunert K.G. (2005). Food quality and safety: Consumer perception and demand. Eur. Rev. Agric. Econ..

[154] Roy-Lachapelle A., Solliec M., Bouchard M.F., Sauve S. (2017). Detection of Cyanotoxins in Algae Dietary Supplements. Toxins.

[155] Herath H.M.U.L., Jayawardana B.C., Fernando P.D.S.M., Weththasinghe P. (2023). A meta-analysis of the effects of dietary *Spirulina* on growth performance of broiler chicken. World’s Poult. Sci. J..

